# Systematic review of the physiological and health-related effects of radiofrequency electromagnetic field exposure from wireless communication devices on children and adolescents in experimental and epidemiological human studies

**DOI:** 10.1371/journal.pone.0268641

**Published:** 2022-06-01

**Authors:** Lambert Bodewein, Dagmar Dechent, David Graefrath, Thomas Kraus, Tobias Krause, Sarah Driessen

**Affiliations:** Research Center for Bioelectromagnetic Interaction (femu)–Institute for Occupational, Social and Environmental Medicine, Medical Faculty, RWTH Aachen University, Aachen, Germany; Consiglio Nazionale delle Ricerche, ITALY

## Abstract

**Background:**

For more than 20 years, the potential health risks of radiofrequency electromagnetic field (RF EMF) exposure from mobile communication devices on children and adolescents have been examined because they are considered sensitive population groups; however, it remains unclear whether such exposure poses any particular risk to them.

**Objectives:**

The aim of this review was to systematically analyze and evaluate the physiological and health-related effects of RF EMF exposures from wireless communication devices (mobile phones, cordless phones, Bluetooth, etc.) on children and adolescents.

**Methods:**

This review was prepared according to the *Preferred Reporting Items for Systematic Reviews and Meta-Analyses* (PRISMA) guidelines. Methodological limitations in individual studies were assessed using the Office of Health Assessment and Translation (OHAT) Risk-of-Bias Rating Tool for Human and Animal Studies.

**Results:**

A total of 42 epidemiological and 11 experimental studies were eligible for this review. Most of the studies displayed several methodological weaknesses that limited the internal validity of the results. Due to a lack of consistency regarding the outcomes as well as the lack of scientific rigor in most reviewed studies, the body of evidence for the effects of RF EMF of mobile communication devices on subjective symptoms, cognition, and behavior in children and adolescents was low to inadequate. Evidence from the studies investigating early childhood development, brain activity, cancer, and physiological parameters was considered inadequate for drawing conclusions about possible effects.

**Discussion:**

Overall, the body of evidence allows no final conclusion on the question whether exposure to RF EMF from mobile communication devices poses a particular risk to children and adolescents. There has been rapid development in technologies generating RF EMF, which are extensively used by children and adolescents. Therefore, we strongly recommend high-quality systematic research on children and adolescents, since they are generally considered as sensitive age groups.

## Introduction

The use of mobile communication devices, such as mobile and cordless phones, has increased significantly since the beginning of the new millennium, especially among children and adolescents [1, 2]. Recent studies indicate that 83% of primary school students in the UK [3] and 95% of the adolescents in the US [4] own or have access to a mobile phone. In Germany, according to a survey conducted by the digital association Bitkom, 54% of children aged between 6 and 7 years use a smartphone from time to time, and 75% of the children aged 10 to 11 years own a mobile phone [5].

Concerns about potential health effects in the younger population caused by exposure to mobile communication devices first appeared in 2000 in the British “Stewart Report” [6]. This report detailed the sensitivity of children and adolescents to radiofrequency electromagnetic fields (RF EMF) with respect to their developing nervous system, the anatomy and physiology of their heads, and the increased exposure duration to EMF from mobile devices due to their longer lifespan compared to adults.

The assumptions of the Stewart Report have had much support [7–11] and the general consensus is that, due to their anatomy and physiology, children absorb higher amounts of energy from mobile phone devices compared to adults and are, therefore, more highly exposed. Some authors [10, 12] see the problem as not only due to the difference in energy absorption, but also because children are still developing and so must be considered more sensitive [13]. However, whether children absorb larger quantities of energy from mobile devices due to different anatomical and physiological characteristics compared to adults is debatable [10, 12, 14–19].

Although many authors [20–25] have reviewed the potential health risks of exposure to EMF from mobile communication devices on children and adolescents, it is still unclear whether such exposure poses any risk to these particular age groups [7, 19, 26–28]. Moreover, the evaluation of studies by Marino et al. (2011) [29] on the effects of EMF emitted by mobile communication devices on young animals could not confirm that prenatal or postnatal exposures were associated with adverse acute or long-term effects, or that young animals were more sensitive than adults. Bektas and Dasdag (2017) [30] also found that data on the effects of exposure to mobile phones on young animals were inconsistent, but they were able to identify many studies showing adverse effects. A current review by Ashrafinia et al. (2021) [31] found only inconsistent results regarding the effects of pre- and postnatal exposure to mobile phone-related EMF on mothers and their children. However, the review was not conducted systematically and only 6 articles were included in the analysis. To date, a systematic analysis and evaluation of the current state of scientific knowledge on the health-related effects of RF EMF on children and adolescents, or on young animals, is lacking.

The aim of this review was to systematically analyze and evaluate the physiological and health-related effects of RF EMF exposure from wireless communication devices (mobile phones, cordless phones, Wireless Local Area Network (WLAN), Bluetooth, base stations, etc.) on children and adolescents.

## Methods

### General information

This review conforms to the *Preferred Reporting Items for Systematic Reviews and Meta-Analyses* (PRISMA) statement [32]. The applied methods were similar to Petri et al. (2017) [33], Schmiedchen et al. (2018) [34], Bodewein et al. (2019) [35], and Driessen et al. (2020) [36]. The search strategy, inclusion and exclusion criteria, and data to be extracted from included articles were specified in a protocol at the beginning of the project.

### Eligibility criteria

The eligibility criteria were defined using the *Population*, *Exposure*, *Comparator*, *Outcome*, *Study design* (PECOS) concept [37]. Journal articles were included in this review when they reported investigations of children and adolescents (0 (after birth)–< 18 years at baseline examination; *Population*) with exposures to RF EMF emitted by mobile communication (e.g., Global System for Mobile Communications (GSM), Universal Mobile Telecommunications System (UMTS)) and other wireless communication devices (e.g., Bluetooth, WLAN) in the frequency range between 800 MHz and 3 GHz (*Exposure*). Studies were only eligible if they investigated participants in at least 2 groups with different quantifiable exposure levels (e.g., duration/number of calls, field strengths; *Comparator*). Additionally, the exposure level had to be determined for the individual. Studies on health-related endpoints, behavior, and other physiological endpoints were considered (*Outcome*). Peer-reviewed journal articles written in English or German were included in this review if they described experimental (single-blind or double-blind) or epidemiological (cohort, case-control, and cross-sectional) studies (*Study design*).

The year of publication was restricted to at least 1990, as it was assumed that there was no significant exposure from mobile communication devices prior to that time.

Excluded were reviews, comments, non-peer-reviewed studies, dosimetric studies, and studies on electromagnetic interference involving implants. Studies investigating the effects of screen time (i.e., when study authors recorded the time spend on the use of electronic equipment, such as mobile phones or tablets, but without the explicit intent of investigating EMF exposure) were excluded. The rationale for this exclusion criterion was that there are several EMF-unrelated factors (e.g., blue light exposure or sleep interruption due to mobile phone use) that may also influence children’s health and that it is impossible to detangle them from EMF-related exposure measures when the authors provide only the parameter “screen time”. Furthermore, studies lacking information on age of the investigated population or without separate analysis of relevant age groups were excluded. Similarly, studies lacking information on the source of EMF or studies investigating frequency spectra, only co-exposures to different EMF sources, or co-exposures with non-EMF exposure were excluded. Moreover, studies without exposure assessment of the individual (e.g., ecological studies or studies including only a single EMF measurement to determine the exposure level for a larger area or for several subjects, e.g., in a classroom) were also excluded from this review, since the individual exposure is highly uncertain in such studies. Because of their particular focus, studies on the effects of the fetus (e.g., length), pregnancy (e.g., miscarriage and preterm birth), birth and/or the newborn child (e.g., birth weight) merit a separate evaluation and were therefore excluded from our review.

### Information sources and search strategy

Relevant journal articles published through December 2021 were identified using electronic database searches in PubMed (U.S. National Library of Medicine, National Institutes of Health) and in the thematically specialized literature database EMF-Portal (www.emf-portal.org). The EMF-Portal is the world’s most comprehensive scientific literature database on biological and health-related effects of EMF. It has been publicly available for more than 15 years and contains currently (as of March 2022) about 35,400 records. In 2017, an evaluation of the EMF-Portal revealed that the database contained 97% of the relevant scientific literature [38]. The identification of studies to be included in the EMF-Portal is based on systematic search strategies in major literature databases, including PubMed and the Institute of Electrical and Electronics Engineers (IEEE) Xplore Digital Library. Scientific journals not listed in these databases, as well as reviews and reference lists of journal articles, are additionally screened to identify further relevant publications. All studies entering the EMF-Portal are categorized according to basic characteristics such as exposure specifications (frequency, type of field) or type of publication (e.g., original research article, review, dosimetric study). This *a priori* categorization enables highly specialized searches.

The search utilized in the EMF-Portal was based on the search terms, “adolescents OR newborn OR fetus OR child OR birth”. All articles containing variations of these terms (e.g., newborns), their synonyms (e.g., teenager), or German translations (e.g., Jugendlicher) were automatically retrieved. The terms were searched in the title, abstract, and Medical Subject Headings (MeSH) terms of the articles. Only the frequency range > 10 MHz was considered in the search.

The search utilized in PubMed was based on a search in title and abstract using the terms "birth" OR "fetus*" "foetus*" OR "newborn*" OR "youth*" OR "teen*" OR "child*" OR "adolescen*" OR "infant*" in combination with a list of several exposure sources like “cell phone” OR “base station”. The links to the search strings in the electronic databases are provided in S1 Links.

EndNote reference management software (www.endnote.com) was used to manage the bibliography and references throughout the manuscript.

### Study selection

Screening for eligibility of all potentially relevant articles was conducted in 2 stages. First, the titles and abstracts of the identified articles were screened by 2 authors (DD, LB, SD, or TK). In the second stage, the full text was retrieved for those publications that met the inclusion criteria, and the articles were independently reviewed by at least 2 authors. The authors jointly made a final decision about the inclusion or exclusion of the reviewed articles.

### Data extraction

Two authors (DD, LB, SD, or TK) independently extracted details regarding the design, methods, and analysis of results of each study. Extracted data included bibliographic data, fundings, Conflicts of Interest (CoI), study design, number and age of study participants (the terms “children” and/or “adolescents” were adopted from the respective study), source of exposure, study focus (endpoint), and results. In addition, for the epidemiological studies, the cohort used, the type and level of exposure (e.g., mobile phone, base station, WLAN, years of mobile phone use, daily number of calls), and the estimation method (e.g., measurement, questionnaire) were recorded. In experimental studies, the number and size of groups, and exposure parameters (e.g., frequency, field strength, exposure duration) were additionally recorded.

### Study appraisal

The internal validity of the included studies (i.e., the extent to which individual studies minimized biases in study design, methods, and analysis of results) and the quality of included studies were assessed using a modified version of the recommended approach by the Office of Health Assessment and Translation (OHAT) of the National Toxicology Program [37, 39]. The OHAT Risk-of-Bias Rating Tool for Human and Animal Studies was developed for studies with a focus on environmental health and toxicology. It consists of a set of questions and provides detailed instructions regarding how to evaluate the credibility of the results, that is, the risk of an over- or underestimation of the exposure effects.

For this review, the OHAT protocol was modified by redefining the rating for the criterion *Identical Experimental Conditions Across Study Groups*. In the original OHAT protocol, this criterion only applied to animal studies. However, in human studies with exposures to EMF, systematic differences between the experimental conditions during exposure versus sham exposure could substantially bias the outcomes as well. Therefore, this criterion is crucial for a well-controlled design in human studies. In contrast, the criterion *Allocation concealment* was not applied in any experimental study because only studies with a crossover design could be identified in the present review, that is, studies without a separate control group. Therefore, 7 criteria were utilized to rate the included epidemiological studies (*Cohort studies*, *Cross-sectional studies* and *Case-control studies*) (Fig 2), while 8 criteria were employed to rate the included experimental studies (*Human controlled trials)* for biases (Fig 3).

The OHAT criteria were independently assessed by 2 authors (DD, LB, SD, or TK) for all included studies according to the following ratings: “++” definitely low risk of bias, “+” probably low risk of bias, “-” probably high risk of bias, or “—” definitely high risk of bias. Disagreements in the assessment were discussed between the authors and resolved by consensus. For more details on the assessed risk of bias domains, questions according to OHAT, and the modifications and specifications done by us, see S1 Table.

Finally, the OHAT approach was utilized to place individual studies into quality categories. This approach outlines a 3-tier system to rate study quality (1^st^ tier: high confidence in the reported results, 2^nd^ tier: moderate confidence in the reported results, or 3^rd^ tier: low confidence in the reported results). The placement of a study into a quality tier was based on the risk-of-bias ratings. Three critical risk-of-bias criteria, called “key criteria,” were given the most weight in determining the study quality. In experimental studies, these key criteria were: (1) *Confidence in the exposure*, (2) *Confidence in the outcome assessment*, and (3) *Identical experimental conditions across study groups*. In epidemiological studies, the key criteria were: (1) *Confidence in the exposure*, (2) *Confidence in the outcome assessment*, and (3*) Confounding and modifying variables*. The ratings for the remaining criteria were given less weight in determining study quality (see S1 Fig for further details regarding study quality ratings).

### Evidence synthesis

We assessed the confidence in the body of evidence according to OHAT guidelines [37], which are based on the GRADE approach. Only studies assigned to be 1^st^ and 2^nd^ tier in the OHAT study quality approach were included in the evidence synthesis. According to OHAT, available studies on a particular outcome were initially grouped by key study design features and each grouping of studies was given an initial confidence rating by those features. This initial rating was downgraded for factors that decrease confidence in the results (e.g., risk of bias and unexplained inconsistency). Typical upgrading factors, such as large magnitude of effect, dose response, and consistency across study designs/populations could not be identified in the body of evidence of the current review. The evidence appraisal was conducted at endpoint level. Four descriptors were used to indicate the level of confidence in the body of evidence: “high”, “moderate”, “low”, and “very low” confidence.

- High Confidence: The true effect is highly likely to be reflected in the apparent relationship.
- Moderate Confidence: The true effect may be reflected in the apparent relationship.
- Low Confidence: The true effect may be different from the apparent relationship.
- Very Low Confidence: The true effect is highly likely to be different from the apparent relationship.

Finally, according to OHAT, the confidence in the body of evidence was translated into 5 descriptors of the evidence for health effects using the confidence ratings and direction of the effect (“health effect” or “no health effect”): “high”, “moderate”, “low”, “evidence of no health effect”, and “inadequate evidence”. “High,” “moderate,” and “low” level of evidence directly translate from the ratings of confidence in the evidence (see above) if the exposure is associated with a health effect. If only a “very low” or “no confidence” in the body of evidence was identified, then the level of evidence was characterized as “inadequate evidence of health effect”. According to OHAT, the descriptor “evidence of no health effect” is used to indicate confidence that the exposure is not associated with a health effect. Because of the inherent difficulty in proving a negative effect, the conclusion “evidence of no health effect” is only reached when there is high confidence in the body of evidence.

## Results

### Study selection

**Fig 1:**
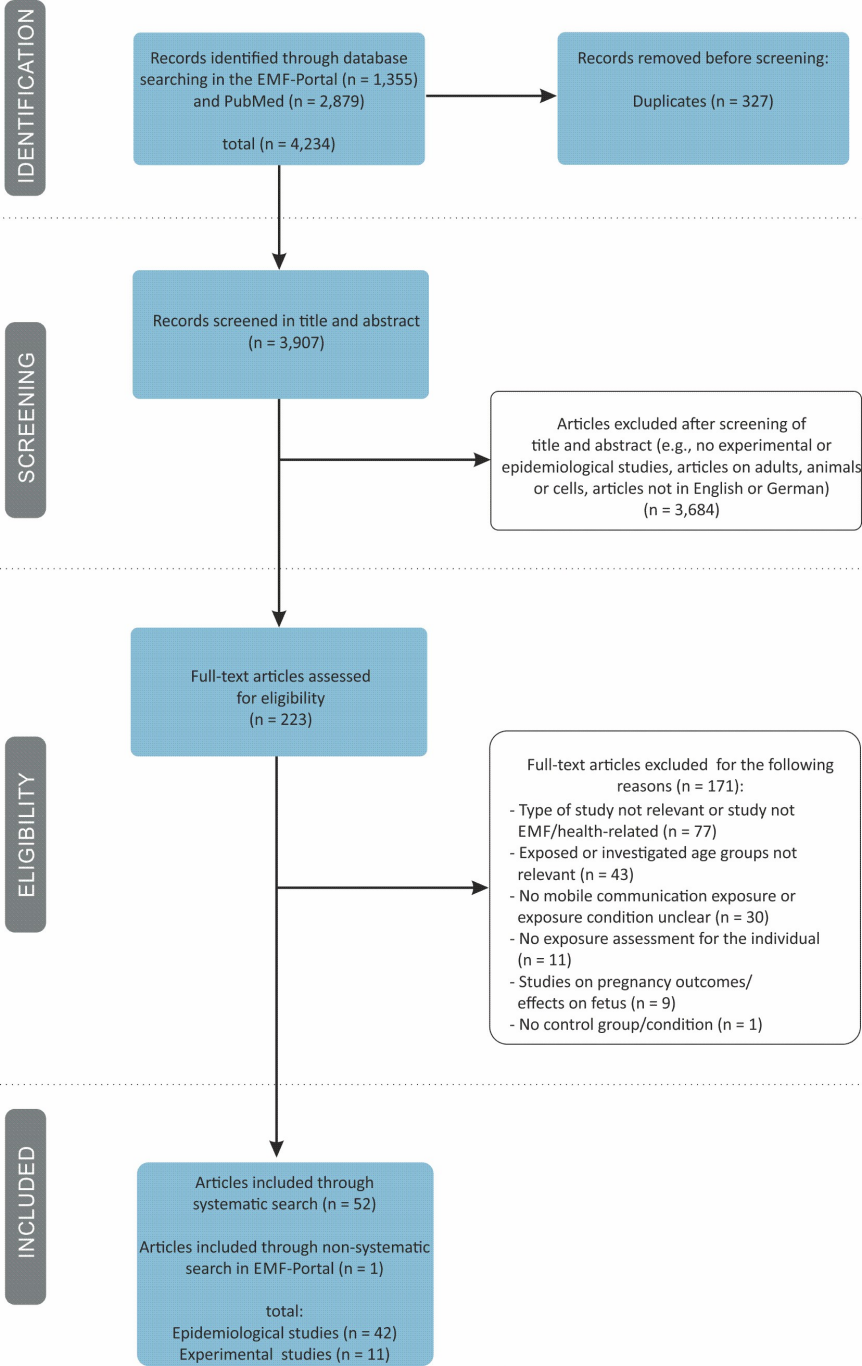
Flow diagram of literature search, eligibility and inclusion process according to PRISMA statement.

The systematic search returned a total of 4,234 articles (1,355 articles form the search in EMF-Portal and 2,879 articles from the search in Pubmed) (Fig 1). After removal of duplicates, 3,907 articles were screened in title and abstract, whereof 3,684 studies were excluded because they did not match the eligibility criteria (e.g., no experimental or epidemiological study). The full texts of the remaining 223 articles were obtained in order to check for their eligibility for inclusion in the current analysis. Of these, 171 articles were excluded for the following reasons: the type of study was not relevant or the study was not EMF/health-related (n = 77), the exposed or investigated age groups were not relevant (n = 43), there was no mobile communication exposure or the exposure was unclear (n = 30), no exposure assessment for the individual was provided (n = 11), the studies concerned pregnancy outcomes or effects on the fetus (n = 9), or there was no control group/condition (n = 1). Some of these articles were excluded because they met more than one exclusion criterion. For reasons of clarity, only the most noteworthy reason for exclusion was documented in Fig 1. A list of all excluded articles, including bibliographic data and the reasons for their exclusion, is provided in the S2 Table. Due to additional non-systematic screening in the EMF‐Portal after December 2021, we identified one additional relevant article [40]. Finally, 53 studies fulfilled the eligibility criteria and were included in this systematic review. Of these, 42 studies were epidemiological studies (with the following endpoints: subjective symptoms, cognitive functions, behavior, infant development, and others) and 11 were experimental studies (with the following endpoints: brain activity, cognitive functions, and physiological parameters).

### Study appraisal

Using the risk-of-bias tool recommended by OHAT [37, 39], the internal validity and the quality of all studies that met the inclusion criteria were assessed.

#### Risk of bias in epidemiological studies

**Fig 2:**
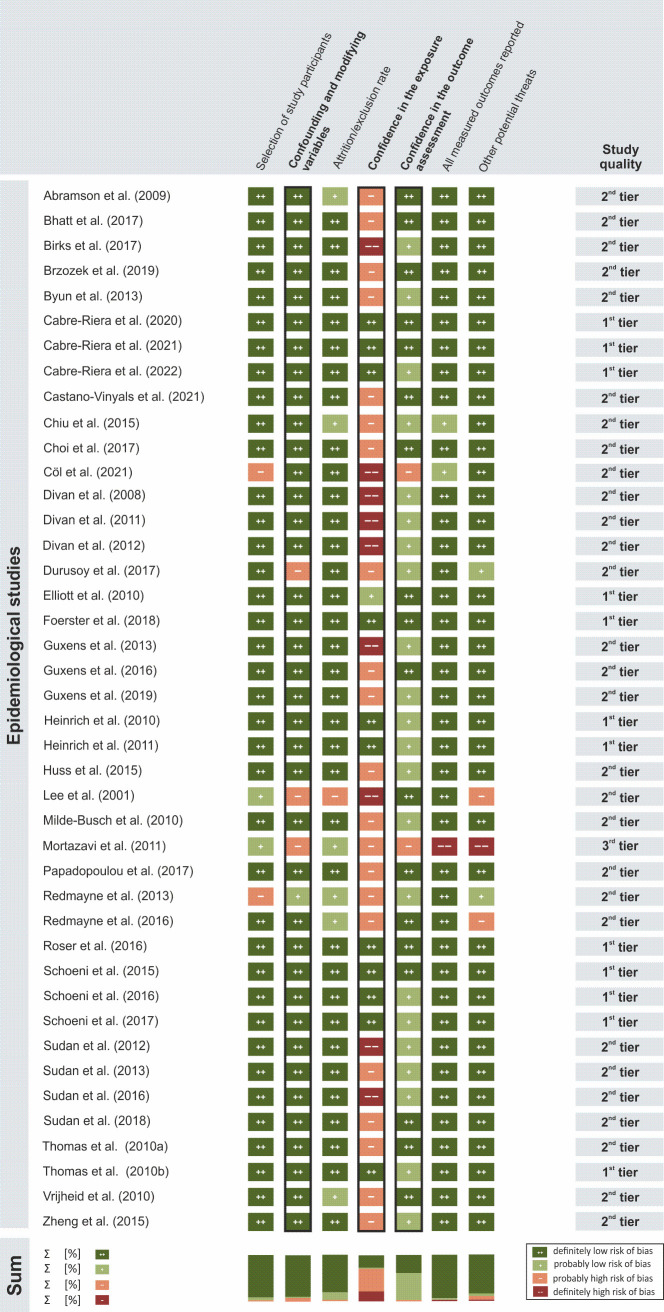
Risk-of-bias ratings for epidemiological studies (n = 42). Criteria ratings served as the basis for the assignment of individual studies to one out of 3 study quality categories (1^st^ tier, 2^nd^ tier, 3^rd^ tier; see S1 Fig). Black frames indicate key risk-of-bias criteria.

Of the 42 included epidemiological studies, 12 (29%) were assigned to the 1^st^ tier, 29 (69%) to the 2^nd^ tier, and 1 (2%) to the 3^rd^ tier (Fig 2).

Methodological flaws were identified in particular regarding the criterion *Confidence in the exposure*. A total of 30 studies (71%) were classified as having a “probably high risk of bias” or “definitely high risk of bias” using this criterion because the exposure was determined by means of a questionnaire filled in either by the children or adolescents themselves, or by a parent. If the questionnaire on mobile phone use was filled in retrospectively (e.g., 5 years after exposure), this criterion was classified as having a “definitely high risk of bias.”

#### Risk of bias in experimental studies

**Fig 3:**
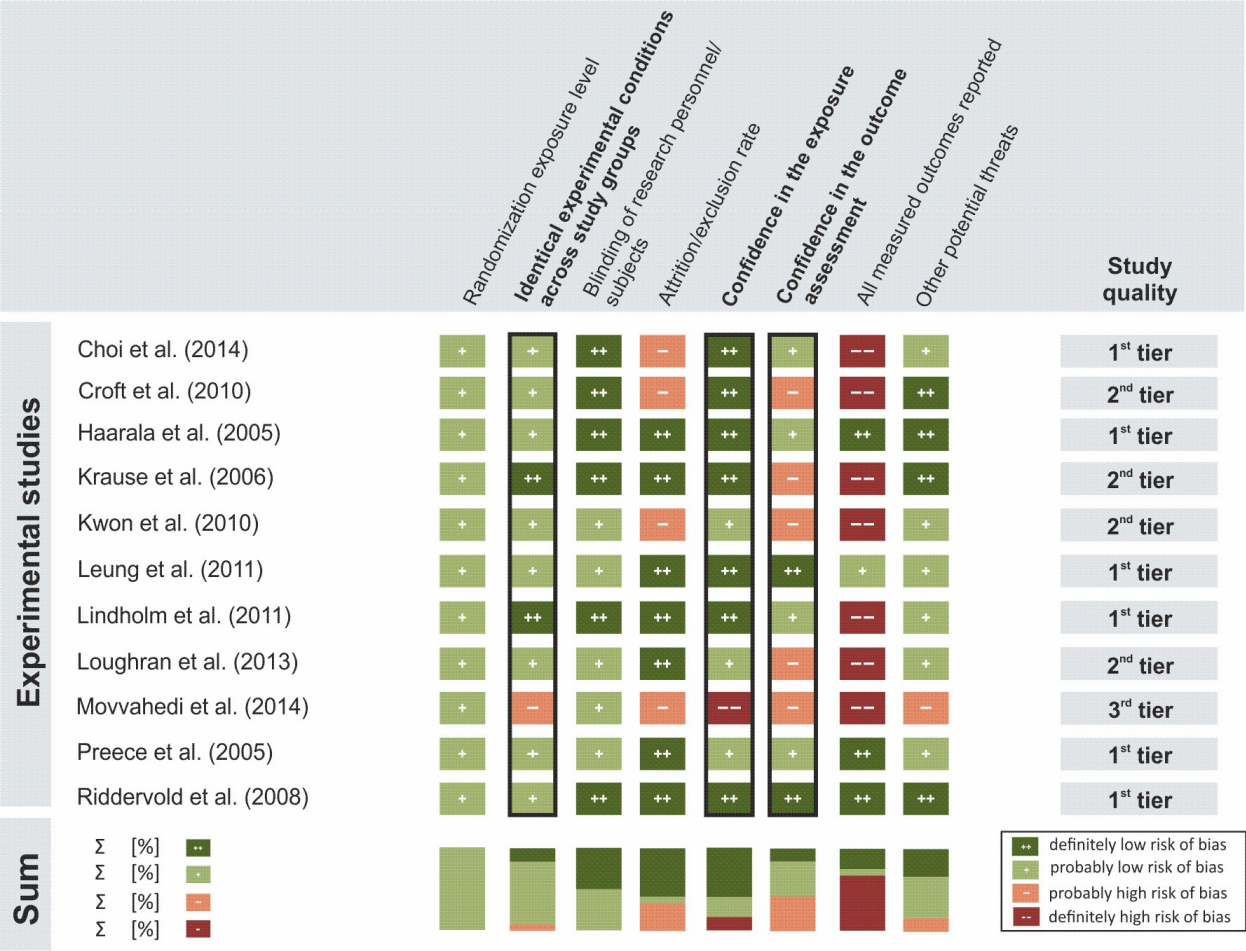
Risk-of-bias ratings for experimental studies (n = 11). Criteria ratings served as the basis for the assignment of individual studies to one out of 3 study quality categories (1^st^ tier, 2^nd^ tier, 3^rd^ tier; see S1 Fig). Black frames indicate key risk-of-bias criteria.

Six of the 11 reviewed experimental studies were placed into the 1^st^ tier, while 4 studies were assigned to the 2^nd^ tier, and one to the 3^rd^ tier (Fig 3).

Methodological flaws were especially common regarding the criterion *All measured outcomes reported*. Seven studies were found to have a “definitely high risk of bias” as the results were not presented completely or in sufficient detail. For example, in several studies, only the mean values for a whole group were provided and no results for the individual. Five studies had a “probably high risk of bias” using the criterion *Confidence in the outcome assessment* because either the study did not state if the research personnel conducting the assessment were blinded or information on the procedure of the outcome assessment was lacking. In 4 studies, the reason why participants from the study were excluded from the analyses (*Attrition/exclusion rate)* was unclear and can thus be considered another “probably high risk of bias”.

### Epidemiological studies

A total of 42 epidemiological studies on mobile communication exposure and health-related effects in children and adolescents between the ages of 6 months and 18 years were identified. The studies were based on cohorts and study populations from 22 different countries, with the Danish National Birth Cohort (DNBC) being the most frequently investigated study population (n = 8). The investigated time period in the 42 studies ranged from 1996 to 2016. Most studies were cohort studies (n = 22) and cross-sectional studies (n = 18). Only 2 case-control studies were identified [40, 41]. The sizes of the study populations varied largely, ranging from 72 adolescents in a Chinese cross-sectional study [42] to 83,884 mother-child pairs in a multinational analysis of 5 birth cohorts [43]. Most publications (n = 40) investigated exposure to mobile phones. A total of 19 studies additionally examined the use of cordless phones (e.g., Digital Enhanced Cordless Telecommunications (DECT)) and 11 studies examined exposure to mobile phone base stations. Two studies [41, 44] investigated solely the exposure to mobile phone base stations, and TV and radio broadcasting transmitters.

Various methods were used for exposure assessment (questionnaires, measurements, or calculations). In most publications (n = 36), questionnaires or interviews were used and answered by the child/adolescent or a parent. In 19 studies, the exposure was assessed (partly in addition to questionnaires) via measurements and/or calculations; 11 of the studies used body-worn personal dosimeters.

The identified studies were divided into 5 categories according to their investigated endpoints: subjective symptoms (n = 14), cognitive functions (n = 12), behavior (n = 9), infant development (n = 4), and others (n = 4). One publication [45] investigated both cognitive functions and behavior and was, therefore, considered in both categories separately.

#### Subjective symptoms

A total of 14 studies on subjective symptoms (e.g., headaches, dizziness, concentration problems, sleeping problems) in children and adolescents were found (S4 Table). Most of these studies (n = 12) examined mobile phone use in children and adolescents. Mobile phone use in pregnant women was investigated by Sudan et al. (2012) [46] and Çöl et al. (2021) [47]. In 5 studies, residential exposure to mobile phone base stations was also assessed; in one study [48], it was the only investigated source of exposure. A total of 5 studies also investigated exposure to cordless phones.

Five studies [47, 49–52] of 2^nd^- and 3^rd^-tier quality reported an association between exposure to mobile communication and different subjective symptoms. In a 3^rd^-tier cross-sectional study from Iran [51], the authors found various subjective symptoms (e.g., headaches, palpitations) following exposure to mobile phones for > 11 minutes/day. There was a high risk of bias in this study due to, for example, insufficient information on the acquisition of data (concerning the endpoints) and exposure estimation via the questionnaire. A cross-sectional study from Taiwan [49] found that children using mobile phones had more headaches, migraines, and skin irritations. In the Chinese cross-sectional study by Zheng et al. (2015) [52], an association was found between fatigue in children and exposure to mobile phones that were used for more than one year. In a cross-sectional study conducted in Turkey [50], an increased risk of headaches, fatigue, and sleep disturbances was found in adolescents using mobile phones. Çöl et al. (2021) [47] found in a cohort study that exposure to electromagnetic fields caused by electronic media devices, e.g., mobile phone or WiFi, during pregnancy was associated with sleep problems in childhood.

In 6 studies [44, 46, 53–56] of 1^st^- and 2^nd^-tier quality, few statistically significant results were found. However, according to the authors, these results should be interpreted with caution because they may, for example, have been based on uncontrolled confounding or caused by chance. Thus, the causality of the associations was not clear. There could also have been a reverse causality, that is, e.g., children could have used their mobile phones more often because they had headaches. In other cases, the results were not consistent across study groups or time points.

Heinrich et al. (2011) [57], Huss et al. (2015) [58], and Milde-Busch et al. (2010) [59] found no or only sporadic associations between mobile phone use or exposure to mobile phone base stations and the occurrence of headaches and other subjective symptoms in children and adolescents in their 2^nd^-tier and 1^st^-tier studies.

#### Cognitive functions

A total of 12 studies on the potential effects of exposure to RF EMF from mobile communication devices on cognitive function in children and adolescents were found (S5 Table). The cognitive functions of interest consisted of, for example, learning, memory, and attention. All studies examined the use of mobile phones, 7 studies additionally investigated cordless phone use, and 3 studies examined exposure to mobile phone base stations.

Two 1^st^-tier studies [48, 60] found consistent evidence for reduced figural memory performance in relation to RF EMF exposure. Schoeni et al. (2015) [48] found reduced figural memory performance in 425 adolescents from the Swiss Health Effects Related to Mobile Phone Use in Adolescents (HERMES) cohort. In a later publication from the HERMES cohort, Foerster et al. (2018) [60] confirmed these results in an enlarged study population of 843 adolescents. The authors concluded that there are preliminary indications for an association between RF EMF exposure and changes in brain functions, like figural memory. However, according to the authors, the results should be interpreted with caution and confirmed in future studies. Lee et al. (2001) [42] found an association between mobile phone use and a mild facilitating effect on attention in their 2^nd^-tier study. However, the authors discuss the possibility that mobile phone users may be naturally better at multi-tasking.

The remaining 9 studies of 1^st^-tier (n = 2) and 2^nd^-tier (n = 7) quality found no consistent evidence for an association between exposure to mobile communication EMF and cognitive function in children and adolescents. Some of these studies found both impairments and improvements in single parameters [61–65] while others found only a few inconsistent [45, 66] or no [67] significant results. Some authors attributed their significant results to the study design or to chance and interpreted them with caution.

#### Behavior

Nine epidemiological studies investigated the effects of exposure to mobile communication devices on behavior (e.g., emotional difficulties, hyperactivity, peer relationship problems; S6 Table). Three studies considered both the strengths (prosocial behavior) and weaknesses (behavioral problems) of the children. In all 9 studies, exposure to mobile phones was investigated. Six studies additionally evaluated exposure to cordless phones, while 3 studies examined exposure to WLAN, and mobile and cordless phone base stations. Prenatal and postnatal exposure data were analyzed in 5 and 7 studies, respectively. Three studies investigated both prenatal and postnatal exposure effects.

Thomas et al. (2010b) [68] and Sudan et al. (2016) [69] found a consistent association between mobile phone use and behavioral difficulties in children and adolescents in their 1^st^- and 2^nd^-tier studies. In their DNBC cohort study, Sudan et al. (2016) [69] found an association between mobile phone use of mothers during pregnancy (prenatal) as well as their 7-year-old children (postnatal) and behavioral problems in the children. This association was already suggested in previous studies of the DNBC cohort [70, 71] but with limited confidence in the validity. An increased risk of behavioral difficulties was also found in children and adolescents exposed to high RF EMF in the German Mobilfunk: Exposition und Befinden (MobilEe) study [68].

The 2^nd^-tier study of Byun et al. (2013) [72] revealed an increased risk of attention deficit hyperactivity disorder (ADHD) and mobile phone use in children with a high blood lead level. A similar effect of combined lead and mobile phone exposures was also discovered by Choi et al. (2017) [73] in association with delayed psychomotor development in infants (see chapter “Infant development”). Birks et al. (2017) [43] and Guxens et al. (2019) [74] both found indications for an association between mobile communication EMF and behavioral problems in their 2^nd^-tier studies. However, the authors stated that these results should be interpreted with caution as they could not rule out residual confounding or reverse causality.

The remaining 2 studies of 1^st^- and 2^nd^-tier quality [45, 75] did not identify any associations between exposure to mobile phone communication devices and behavioral difficulties.

#### Infant development

Four studies concerning the effects of mobile phone use on infant development were identified (S7 Table). Important endpoints of infant development that were examined included, for example, mental development and motor skills.

All 4 studies investigated mobile phone use of mothers during pregnancy. Additionally, Choi et al. (2017) [73] determined the general RF EMF exposure of the mothers (e.g., by radio and TV transmitters) using personal exposimeters. The 4 epidemiological studies on infant development found few [76, 77] or no associations [73, 78] of mobile phone exposure of mothers during pregnancy and the development of their children up to the age of 5 years. However, Choi et al. (2017) [73] suggested a potential synergistic effect of prenatal exposure to lead and mobile phone use, i.e., children of mothers with an increased blood lead level who used a mobile phone during pregnancy had a higher risk of delayed psychomotor development. A similar combined effect of lead and mobile phone use was also revealed by Byun et al. (2013) [72] in association with ADHD (see chapter “Behavior”). All 4 of these studies were assigned to the 2^nd^ tier.

#### Other endpoints

In addition to the studies on subjective symptoms, cognition, behavior and infant development, 2 studies on childhood cancer [40, 41], one study [79] on hearing loss and a further study on brain volume [80] were identified (S8 Table). In a 1^st^-tier study, Elliott et al. (2010) [41] did not find an association between maternal exposure to mobile phone base stations during pregnancy and the risk of cancer in their children. Likewise, Castano-Vinyals et al. (2021) [40] did not find any association between brain tumors and the use of mobile communication devices in children and adolescents in their 2^nd^-tier study. Sudan et al. (2013) [79] used 3 different statistical analyses and, in one of the analyses, found a statistically significant association between mobile phone use of 7-year-old children and hearing loss. Cabré-Riera et al. (2020) [80] found a single statistically significant association between screen activities and a smaller caudate volume in children among several other non-significant associations. The authors of these 2 studies with limited associations concluded, however, that these associations may not have been causal and should be interpreted with caution. The quality of these studies was evaluated as 2^nd^ tier and 1^st^ tier, respectively.

### Experimental studies: Effects on children

A total of 11 experimental studies investigated the effects of exposure to mobile communication EMF on children and adolescents. The majority of the studies investigated the effects of exposure on brain activity and cognitive function (n = 9) (S9 Table), while the remaining 2 studies investigated different physiological parameters (e.g., heart rate and respiratory rate; S10 Table).

#### Brain activity (EEG) and cognitive function

Nine studies investigated the effects of mobile communication RF EMF (894–2,140 MHz) on brain activity via electroencephalography (EEG) and cognitive functions in children and adolescents (15 to 60 individuals aged 7 to 16 years) (S9 Table). Eight studies investigated exposure to mobile phones (near field) and one study investigated far-field conditions (exposure to mobile phone base stations [81]).

Four studies [81–84] investigated the effects of mobile communication RF EMF on cognitive functions, such as attention, memory performance, and reaction time. Three studies [85–87] examined brain activity (via EEG) at rest versus during a cognitive task, while 2 studies [88, 89] investigated both cognitive function and brain activity. Riddervold et al. (2008) [81] additionally recorded subjective symptoms like vertigo and nausea. Of the 9 studies, 3 found an effect of mobile communication exposure. Krause et al. (2006) [86] found an effect of exposure to a GSM 902 MHz mobile phone on brain activity in the EEG bands 4–8 Hz and around 15 Hz during a memory performance task. The authors of this 2^nd^-tier study concluded that the results provided no evidence for possible health-related effects on cognition. In their 1^st^-tier study, Leung et al. (2011) [88] found a significantly increased N1 amplitude of the event-related potential (ERP) after exposure to a 2^nd^-generation (2G: GSM) mobile phone, a decrease in performance on the N-back test after exposure to a 3^rd^-generation (3G: Wideband Code Division Multiple Access (WCDMA)) mobile phone, and delayed event-related desynchronization/synchronization responses of the alpha power after 2G and 3G exposure. The authors concluded that exposure to mobile phones, especially 3G ones, could affect the working memory and brain function in adolescents. However, the study could not provide any data on potential underlying mechanisms of action. In a 3^rd^-tier study, Movvahedi et al. (2014) [83] observed significant improvement in short-term memory after exposure to a GSM 900 MHz mobile phone, but the statistical significance of this result remained unclear. The remaining 6 studies did not find any effects of mobile communication devices on brain activity or cognitive function.

#### Physiological parameters

Two studies [90, 91] investigated the effects of mobile communication RF EMF on several physiological parameters in adolescents (26 participants each, aged 14 to 17 years; S10 Table). They did not find any effects of exposure to GSM (902 MHz) or WCDMA (1,950 MHz) on local cerebral blood flow, electrocardiogram results, or blood pressure. Both studies were assigned to the 1^st^ tier.

### Study overview

**Fig 4:**
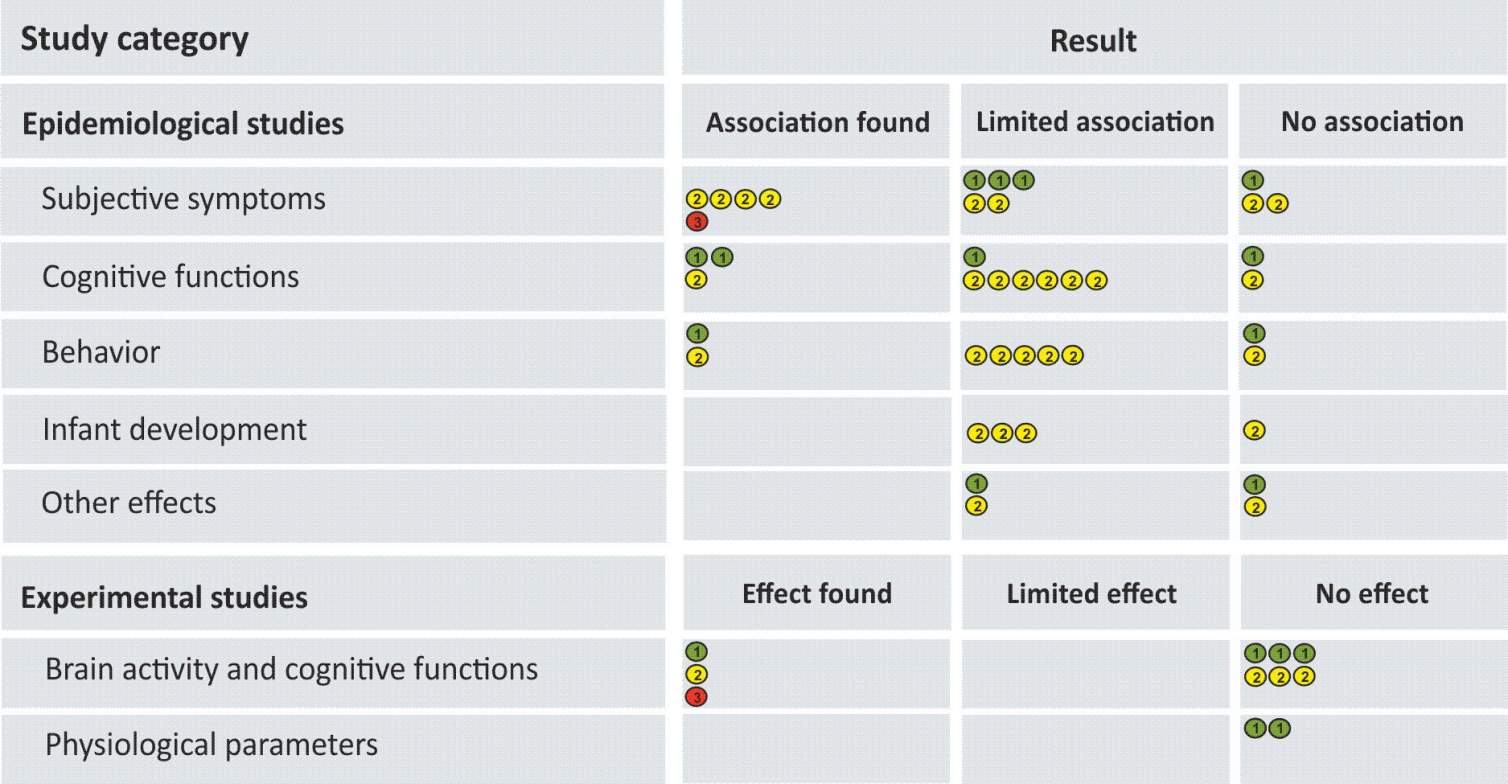
Overview of study quality and effects/associations found in the included studies. Each study is represented by a numbered and colored point, indicating the study quality assessed with the risk-of-bias tool recommended by OHAT [37, 39] (1/green = 1^st^ tier, 2/yellow = 2^nd^ tier, 3/red = 3^rd^ tier). Each study is allocated to an effect category (columns) according to the conclusive result stated by the authors (cf. S4–S10 Tables). Please note that Roser et al. (2016) [45] investigated both cognitive functions and behavior and was therefore considered in both lines separately.

Out of 53 studies, 13 studies (25%) described an effect of wireless communication exposure on children and adolescents or an association between wireless communication exposure and children and adolescents, respectively. 23 studies (43%) found limited associations and 17 studies (32%) found no consistent effect/association. Moreover, only 18 studies were assessed as 1^st^ tier (34%), resulting in a major proportion of studies with a significant risk of bias (Fig 4). Studies were assigned to the categories “effect/association”, “limited association” and “no effect/association” according to the conclusions of the study authors. The category “limited associations” was used in epidemiological studies which described only few and/or inconsistent associations that were interpreted with caution by the study authors and which may have been caused by factors other than RF EMF (Fig 4).

Majority of the studies (66%) received exclusively public funds (e.g., from governmental authorities or universities) and 23% of the studies were supported by different institutions (i.e., public, industry, and/or non-governmental organizations (NGO)). None of the included studies solely received industry funds and only one study was exclusively funded by a non-governmental organization. As the funding sources were relatively balanced and exclusive industry funding was absent across the included studies, we considered the risk of bias due to funding in the current review to be negligible [92–94]. Regarding CoI of study authors, only 6 studies (11%) reported a CoI, the majority of the studies reported no CoI (51%), or CoI statement was lacking (38%). Thus, no significant bias from CoI on the study results could be identified or rather, it could not be estimated, since a significant part of these studies did not provide any details on potential CoI. See S3 Table for details.

## Discussion

### Summary of evidence in epidemiological studies

From a total of 42 epidemiological studies, 10 studies found consistent associations between mobile phone exposure and health effects on children and adolescents, 23 studies found limited associations, and 9 studies did not find any consistent associations (Fig 4).

Nine of the 14 studies on subjective symptoms were cross-sectional studies with a low initial confidence rating [37]. The remaining 5 studies were cohort studies with a moderate initial confidence rating. Due to the risk of bias in the majority of studies (8 at the 2^nd^ tier) and the inconsistent results (4 studies with associations, 3 studies with no associations, and 6 studies with limited associations), the initial confidence rating for the evidence was downgraded to low to very low. Hence, the evidence from these studies regarding an association between mobile communication exposure and subjective symptoms in children and adolescents is low to inadequate (S11 Table).

Seven out of the 12 studies on cognitive function in children and adolescents were cohort studies with a moderate initial confidence rating. Five studies were cross-sectional studies with a low initial confidence rating. Due to the risk of bias in both types of studies (8 at the 2^nd^ tier) and the heterogeneous results (largely inconsistent outcomes, improvements, and impairments of individual parameters), we decided to downgrade the initial confidence rating. Thus, the confidence rating for the body of evidence was low to very low, and the evidence for a health effect of mobile communication device exposure on cognitive function in children and adolescents was rated as low to inadequate (S11 Table).

Seven of the 9 epidemiological studies on behavior in children and adolescents were cohort studies, which received an initial confidence rating of moderate. Overall, the results of these studies were inconsistent, and the study quality was moderate, reducing the final confidence rating to very low. Two additional cross-sectional studies also only found partially consistent associations [68, 74]. In conclusion, the confidence rating for the body of evidence from the 9 studies was low to very low, and there was only low or inadequate evidence for an association between mobile communication exposure and changes in behavior in children and adolescents (S11 Table).

The results of the 4 cohort studies of 2^nd^-tier quality on infant development provided only inconsistent data for an association between maternal mobile phone usage during pregnancy and changes in early development of their children up to age 5. Therefore, the moderate initial confidence rating of the evidence was reduced to very low. Finally, the evidence from these studies was inadequate to draw a conclusion regarding any health effects of mobile communication device exposure on infant development (S11 Table).

Neither Castano-Vinyals et al. (2021) [40] nor Elliott et al. (2010) [41] found an association between exposure to mobile communication EMF and cancer incidence in children. We evaluated these 2 case-control studies with an initial moderate confidence rating as inadequate to provide evidence that there were no effects of mobile phone base station exposure on childhood cancer. The evidence for effects on hearing loss [79] and brain volume [80] was also inadequate as the confidence rating for the body of evidence was very low because it was based on single cross-sectional studies with weak associations.

In summary, the evidence for physiological and health-related effects of mobile communication RF EMF (mobile phones, wireless phones, WLAN, Bluetooth, etc.) on children and adolescents was rated as low to inadequate overall, based on the included epidemiological studies in this review.

### Summary of evidence in experimental studies

Out of a total of 11 experimental studies, 3 of them including a total of 116 children and adolescents found an effect of mobile phone exposure on brain activity and cognition, whereas the remaining 8 studies, with a total of 222 children and adolescents, could not identify any effects (Fig 4).

In general, experimental studies (Human controlled trials) receive a high initial confidence rating according to OHAT [37]. Although one 1^st^-tier study [88] found a significant change in a cognitive task due to mobile phone-related exposure, 3 other 1^st^-tier studies and one 2^nd^-tier study did not find any effect of mobile communication exposure on cognitive function. Due to the inconsistent results across the studies and due to the small study populations (18 to 40 participants) in the studies without effects (allowing for the detection of strong effects only), the evidence was inadequate to draw a conclusion (S12 Table).

There were three 2^nd^-tier studies that did not find effects on brain activity. In contrast, one 1^st^-tier and one 2^nd^-tier study revealed significant modifications in brain activity due to mobile phone-related exposure. However, similar to the studies on cognitive functions, the evidence was considered inadequate due to inconsistent results across the studies, unclear relevance to human health, the risk of bias in the studies, and the small study populations (17 to 41 participants) in the studies without any effects (S12 Table).

Although the two 1^st^-tier studies by Choi et al. (2014) [90] and Lindholm et al. (2011) [91] did not reveal any mobile phone exposure effects on heart rate, respiratory rate, or blood flow, the body of evidence for no effects was considered inadequate due to the small number of studies and the small study populations investigated (n = 26, respectively) (S12 Table).

Four of the 11 included experimental studies also examined adults [81, 85, 88, 90]. Leung et al. (2011) [88] measured reduced performance during a cognitive task (N-back task) in adolescents only. All other parameters did not show any differences between the 2 age groups. The remaining 3 studies did not find different results for adolescents versus adults.

Overall, due to the inconsistent evidence in the reviewed experimental studies, and especially in those studies including different age groups, it remains unclear whether children and adolescents are more sensitive to mobile phone exposure compared to adults.

As the experimental studies included in this review only investigated acute and short-term effects, no conclusion can be drawn regarding potential long-term effects. In summary, the evidence from the included experimental studies is inadequate to draw a conclusion regarding mobile phone-related exposure and its effects on cognition, brain activity, and physiological changes in children and adolescents.

### Summary of other lines of research

In the current review, the most investigated endpoints were subjective symptoms, cognitive function, behavior, infant development, and brain activity. Subjective symptoms or idiopathic environmental intolerance attributed to electromagnetic fields is a controversial condition. In general, the evidence of data in adults points toward no effect of exposure or, at least, a potential effect seems to be very weak or affect only few individuals [34]. Studies in human adults and animals revealed both favorable, unfavorable, and no effects on cognition and behavior [21, 95, 96]. The studies on children included in our systematic review also showed improvements and impairments of individual parameters of cognitive function or inconsistent data regarding behavior. It is more or less accepted that RF EMF exposure can affect brain activity in adults; however, the implications for human health remain unclear [21, 97]. In addition, inconsistencies between studies need to be elucidated in future investigations [98]. In previous animal studies focusing on developmental issues, mostly congenital disorders and some teratogenic effects were reported; however, this was only the case for studies investigating exposure levels far above the limits [99]. Thus, the data from these studies are not comparable to those of the included studies of our systematic review, neither concerning the endpoints nor the exposure condition.

Although there were only 2 studies included in our review investigating the potential effects of RF EMF on cancer, we want to mention that the International Agency for Research on Cancer [100] classified RF EMF as possibly carcinogenic to humans. In addition, the potential carcinogenic effect has attracted further attention due to 2 recent animal studies underpinning a potential harmful effect [101, 102].

However, the implications of RF EMF exposure on human health are controversially discussed in the scientific community. Therefore, in 2019, the WHO commissioned 6 major topics that need systematic analysis and synthesis of the available evidence on potential health-related effects of exposure to RF EMF [103].

### Research needs

The current systematic review returned only 2 epidemiological studies on the association of mobile communication-related exposure and the risk of cancer in children and adolescents. Therefore, we recommend further epidemiological studies on the risk of cancer, especially because the IARC (2013) [100] classified RF EMF as possibly carcinogenic to humans. However, the realization of future case-control studies may be difficult as the majority of children and adolescents today already use mobile phones (see chapter “Introduction”), reducing the number of appropriate control subjects without exposure. We recommend that children and adolescents should be considered separately in future trend studies on the incidence of brain tumors. As the results of the epidemiological studies included in this review on behavior, cognitive functions, and subjective symptoms provided only inadequate or low evidence for effects, further cohort studies with an improved exposure assessment, that is, with objective prospective exposure data, should be performed to elucidate any potential health-related effects. Cross-sectional studies with subjective exposure data should be avoided as confidence in the evidence provided is low.

Based on the conclusion of our systematic review, the call from WHO [104, 105] for high-quality provocation studies investigating the effects of RF EMF on the nervous system (by examining EEG and effects on cognition) remains relevant. Future studies should include larger study populations and different age classes, specifically minimize bias risks regarding the key factors of blinding and exposure assessment, and include a full description of the results.

### Limitations

A limitation of the present systematic review is that the search terms used in identifying relevant journal articles may not have been found in the title, abstract, or MeSH terms of certain articles, such that searches using the EMF-Portal and PubMed did not return all potentially relevant articles. Moreover, we only considered peer-reviewed articles written in English or German. Therefore, potentially relevant data from articles published in other languages, or data from gray literature (data that are not published in scientific journals), were not included.

Several studies (n = 171; see S2 Table), which had previously been classified as potentially relevant during the screening step (see “Study selection”), were ultimately excluded from the review. The main reasons for this were that the studies: (1) investigated age groups that included subjects exceeding the predefined maximum age of 18 years (e.g., [106–109]); (2) investigated RF EMF exposure sources other than mobile communication devices, such as TV and radio broadcasts (e.g., [110]) or therapeutic devices [111]; (3) provided no exposure assessment for the individual but only a comparison of groups in ecological studies (e.g., [112, 113]) or trend studies [114, 115]; (4) examined the effects on the fetus, pregnancy (e.g., miscarriage), birth and/or the newborn child (e.g., [116, 117]). Moreover, animal studies with young animals were not considered. These studies were not included in the review due to the exclusion criteria in our study protocol (see chapter “Eligibility criteria”). Nevertheless, these studies might also provide information about biological and health-related effects of RF EMF exposure on children and adolescents, albeit in a less direct manner or broader sense.

As the OHAT risk-of-bias tool was used to assess the risk of bias in each study as a whole, the assessment utilizing the “confidence in the exposure” criterion might not represent the true risk of bias in every individual exposure assessment method in epidemiological studies with different exposure sources and assessment methods (e.g., mobile phone use assessed by questionnaire, mobile phone base station exposure assessed by measurements). This may have led to an over- or underestimation in the risk of bias in single epidemiological studies.

Finally, we did not conduct any further qualitative (e.g., mobile phone vs. mobile phone base station) or quantitative analyses (e.g., meta-analyses) because we did not expect that further analyses of studies with low or inadequate evidence would aid in answering our key question. However, the included studies might provide sufficient homogeneous data regarding, for example, exposure, exposure metrics, or endpoints to conduct meta-analyses. These may provide further valuable insights into the influence of single parameters (e.g., study design, methods of exposure assessment, age groups) on the outcome.

## Conclusion

In this review, 42 epidemiological and 11 experimental studies on children and adolescents were systematically researched, analyzed, and assessed in view of the health-related effects of RF EMF from wireless communication devices (mobile phones, cordless phones, WLAN, Bluetooth, etc.). A total of 50 studies investigated mobile phone usage, 3 studies examined the exposure to mobile phone base stations, and 22 studies investigated both mobile phone usage and exposure to cordless phones, mobile phone base stations, etc.

Of a total of 53 included studies, 35 studies had several methodological weaknesses, which limited the internal validity of the results. Overall, evidence for the effects of RF EMF of mobile communication devices on subjective symptoms, cognition, and behavior in children and adolescents was considered to be low to inadequate. Furthermore, the studies investigating early childhood development, brain activity, cancer, and physiological parameters were considered inadequate to draw conclusions concerning possible effects. Based on the studies included in this review, it remains unclear whether children and adolescents are particularly sensitive to mobile communication exposure.

In summary, we could not identify a high evidence for any significant detrimental health effects of RF EMF of mobile communications on children and adolescents. Nevertheless, we do not conclude that such exposure would be safe for this particular age group, since the evidence base for this conclusion is too weak.

There has been rapid development in technologies generating RF EMF, which are extensively used by children and adolescents. Therefore, we strongly recommend high-quality systematic research on children and adolescents, since they are generally considered as sensitive age groups [13]. For example, cohort studies with improved exposure assessments and experimental studies investigating the nervous systems, including larger study populations and different age groups, should be conducted. Moreover, children and adolescents should be considered separately in future trend studies.

The conclusions of this review are largely in line with the evaluation of the Scientific Committee on Emerging and Newly Identified Health Risks (SCENIHR) [28] and the conclusions of other authors of earlier studies [7, 26, 27, 118].

## Supporting information

S1 Links Links to search strings for search in the EMF-Portal and PubMed. (DOCX)

S1 Checklist PRISMA 2009 checklist. (DOCX)

S1 Fig Placement of individual studies into one of 3 quality categories (1^st^ tier, 2^nd^ tier, 3^rd^ tier) at the example of human controlled trials. Based on risk-of-bias ratings for the applied criteria: “++” definitely low risk of bias, “+” probably low risk of bias, “-” probably high risk of bias, or “—” definitely high risk of bias. Adapted from the approach recommended by the National Toxicology Program’s Office of Health Assessment and Translation (NTP 2015; 2019). To be placed into the 1^st^ tier, a study had to be rated as “definitely low risk of bias” or “probably low risk of bias” for all key criteria. Additionally, ≥ 50% of the remaining criteria had to be rated as “definitely low risk of bias” or “probably low risk of bias”. To be placed into the 3^rd^ tier, a study had to be rated as “definitely high risk of bias” or “probably high risk of bias” for all key criteria. Moreover, ≥ 50% of the remaining criteria had to be rated as “definitely high risk of bias” or “probably high risk of bias”. Studies which could neither be assigned to the 1^st^ tier nor 3^rd^ tier were placed into the 2^nd^ tier. Please note that the above figure shows the process at the example of Human controlled trials. However, it is applied to epidemiological studies in the same way except for the partly changed criteria and key criteria mentioned in chapter 2.6 Study appraisal.
(TIF)

S1 Table Risk of Bias (RoB) domains and questions according to OHAT. The OHAT Risk of Bias Rating (RoB) Tool for Human and Animal Studies (NTP 2015) was followed as precisely as possible for every assessed RoB criterion. However, especially with a view on the peculiarities of EMF exposure, some specifications and amendments had to be applied on the tool. In the following, these modifications are presented for each RoB criterion. Background information and further elucidations on each RoB domains can be found in OHAT RoB tool (NTP 2015).
(DOCX)

S2 Table All excluded articles including bibliographic data and the reasons for their exclusion after checking for eligibility. (DOCX)

S3 Table Extracted funds and conflicts of interests. (DOCX)

S4 Table Epidemiological studies on subjective symptoms in children and adolescents (n = 14). (DOCX)

S5 Table Epidemiological studies on cognitive function in children and adolescents (n = 12). (DOCX)

S6 Table Epidemiological studies on behavior in children and adolescents (n = 9). (DOCX)

S7 Table Epidemiological studies on infant development (n = 4). (DOCX)

S8 Table Epidemiological studies on other endpoints of children and adolescents (n = 4). (DOCX)

S9 Table Experimental studies on brain activity and cognition in children and adolescents (n = 9). (DOCX)

S10 Table Experimental studies on physiological parameters in children and adolescents (n = 2). (DOCX)

S11 Table Assessing confidence in the body of evidence in epidemiological studies (cohort studies, cross-sectional studies), according to OHAT handbook. (DOCX)

S12 Table Assessing confidence in the body of evidence in experimental studies (human controlled trials), according to OHAT handbook. (DOCX)
