# Supplementary material for: Systematic review of the physiological and health-related effects of radiofrequency electromagnetic field exposure from wireless communication devices on children and adolescents in experimental and epidemiological human studies
Source: PLoS One. 2022 Jun 1;17(6):e0268641. doi: 10.1371/journal.pone.0268641 (PMC9159629; doi:10.1371/journal.pone.0268641)
Supplement: S1 Links — (DOCX) [file pone.0268641.s001.docx]

**S1 Links. Links to search strings for search in the EMF-Portal and PubMed.**

The electronic database searches can be repeated using the following links:

**PubMed**

https://pubmed.ncbi.nlm.nih.gov/?term=%28+%09%22birth*%22%5BTIAB%5D+%09OR+%22fetus*%22%5BTIAB%5D+%09OR+%22foetus*%22%5BTIAB%5D+%09OR+%22newborn*%22%5BTIAB%5D+%09OR+%22youth*%22%5BTIAB%5D+%09OR+%22teen*%22%5BTIAB%5D+%09OR+%22child*%22%5BTIAB%5D+%09OR+%22adolescen*%22%5BTIAB%5D+%09OR+%22infant*%22%5BTIAB%5D+%29+AND+%28+%09%22Base+Station*%22%5BTIAB%5D+%09OR+%22Cell+Phone*%22%5BTIAB%5D+%09OR+Cellphone*%5BTIAB%5D+%09OR+%22Cellular+Phone*%22%5BTIAB%5D+%09OR+%22Cellular+Telephone*%22%5BTIAB%5D+%09OR+%22Mobile+Phone*%22%5BTIAB%5D+%09OR+%22Mobile+Telephone*%22%5BTIAB%5D+%09OR+%22Cordless+Phone*%22%5BTIAB%5D+%09OR+%22Car+Phone*%22%5BTIAB%5D+%09OR+Smartphone*%5BTIAB%5D+%09OR+%22Smart+Phone*%22%5BTIAB%5D+%09OR+Wi-Fi%5BTIAB%5D+%09OR+Wifi%5BTIAB%5D+%09OR+%22Global+System+for+Mobile+Communication*%22%5BTIAB%5D+%09OR+GSM%5BTIAB%5D+%09OR+%22Digital+Cellular+System*%22%5BTIAB%5D+%09OR+%22Universal+Mobile+Telecommunication+System*%22%5BTIAB%5D+%09OR+UMTS%5BTIAB%5D+%09OR+WiMAX%5BTIAB%5D+%09OR+Bluetooth%5BTIAB%5D+%09OR+%22Digital+Enhanced+Cordless+Telecommunication*%22%5BTIAB%5D+%09OR+%225G%22%5BTIAB%5D+%29+NOT+%28+%09app%5BTIAB%5D+%09OR+apps%5BTIAB%5D+%09OR+%22mobile+application*%22%5BMesh%5D+%09OR+%22Telemedicine%22%5BMesh%5D+%09OR+adherence*%5BTIAB%5D+%09OR+mHealth%5BTIAB%5D+%09OR+nomophobia%5BTIAB%5D+%09OR+cyberbull*%5BTIAB%5D+%09OR+%22Genitourinary+Syndrome+of+Menopause%22%5BTIAB%5D+%09OR+%22problematic+smartphone+use%22%5BTIAB%5D+%09OR+%22problematic+cell+phone+use%22%5BTIAB%5D+%09OR+phubbing%5BTIAB%5D+%09OR+addiction%5BTIAB%5D+%09OR+addictive%5BTIAB%5D+%09OR+covid%5BTIAB%5D+%29+AND+%28english%5BFilter%5D+OR+german%5BFilter%5D%29+AND+1990%3A2022%5Bpdat%5D

**EMF-Portal**

https://www.emf-portal.org/de/article/search/results?keywords=adolescents+newborn+foetus+child+birth&logicalOperator=1&authors=&authorMatchingMode=0&journals=&journalMatchingMode=0&years=1990-2020&topics%5B%5D=0&topics%5B%5D=1&topics%5B%5D=2&topics%5B%5D=3&topics%5B%5D=4&topics%5B%5D=5&topics%5B%5D=6&topics%5B%5D=7&topics%5B%5D=8&topics%5B%5D=9&frequencyRanges%5B%5D=0&frequencyRanges%5B%5D=1&timeSpan=0
