# Supplementary figures and images for: Systematic review of the physiological and health-related effects of radiofrequency electromagnetic field exposure from wireless communication devices on children and adolescents in experimental and epidemiological human studies
Source: PLoS One. 2022 Jun 1;17(6):e0268641. doi: 10.1371/journal.pone.0268641 (PMC9159629; doi:10.1371/journal.pone.0268641)

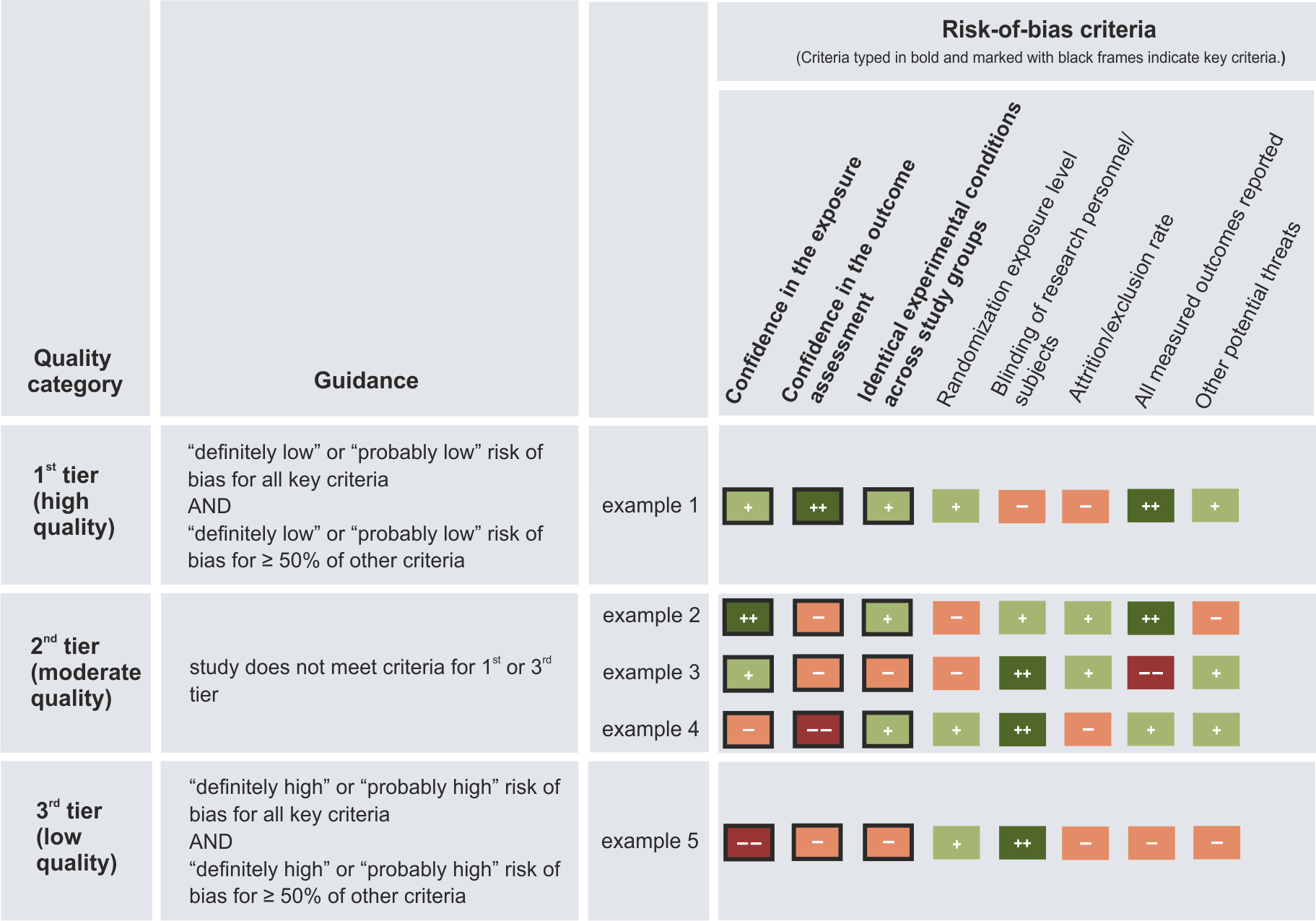

Supplement: S1 Fig — Based on risk-of-bias ratings for the applied criteria: “++” definitely low risk of bias, “+” probably low risk of bias, “-” probably high risk of bias, or “—” definitely high risk of bias. Adapted from the approach recommended by the National Toxicology Program’s Office of Health Assessment and Translation (NTP 2015; 2019). To be placed into the 1st tier, a study had to be rated as “definitely low risk of bias” or “probably low risk of bias” for all key criteria. Additionally, ≥ 50% of the remaining criteria had to be rated as “definitely low risk of bias” or “probably low risk of bias”. To be placed into the 3rd tier, a study had to be rated as “definitely high risk of bias” or “probably high risk of bias” for all key criteria. Moreover, ≥ 50% of the remaining criteria had to be rated as “definitely high risk of bias” or “probably high risk of bias”. Studies which could neither be assigned to the 1st tier nor 3rd tier were placed into the 2nd tier. Please note that the above figure shows the process at the example of Human controlled trials. However, it is applied to epidemiological studies in the same way except for the partly changed criteria and key criteria mentioned in chapter 2.6 Study appraisal. (TIF) [file pone.0268641.s003.tif]
