# Supplementary material for: Systematic review of the physiological and health-related effects of radiofrequency electromagnetic field exposure from wireless communication devices on children and adolescents in experimental and epidemiological human studies
Source: PLoS One. 2022 Jun 1;17(6):e0268641. doi: 10.1371/journal.pone.0268641 (PMC9159629; doi:10.1371/journal.pone.0268641)
Supplement: S1 Table — The OHAT Risk of Bias Rating (RoB) Tool for Human and Animal Studies (NTP 2015) was followed as precisely as possible for every assessed RoB criterion. However, especially with a view on the peculiarities of EMF exposure, some specifications and amendments had to be applied on the tool. In the following, these modifications are presented for each RoB criterion. Background information and further elucidations on each RoB domains can be found in OHAT RoB tool (NTP 2015). (DOCX) [file pone.0268641.s004.docx]

**S1 Table. Risk of Bias (RoB) domains and questions according to OHAT.**

The OHAT Risk of Bias Rating (RoB) Tool for Human and Animal Studies (NTP 2015) was followed as precisely as possible for every assessed RoB criterion. However, especially with a view on the peculiarities of EMF exposure, some specifications and amendments had to be applied on the tool. In the following, these modifications are presented for each RoB criterion. Background information and further elucidations on each RoB domains can be found in OHAT RoB tool (NTP 2015).

| **Selection Bias** | |
| --- | --- |
| **Question 1: Was administered dose or exposure level adequately randomized?** | |
| Cohort (Co), Case-Control (CaCo), Cross-sectional (CrSe) | Human Controlled Trial (HCT) |
| Definitely Low Risk of Bias (++) | |
| n.a. | - direct evidence that subjects were allocated to any study group including controls using a method with a random component   **OR**   - direct evidence that sequences of exposure were allocated using a method with a random component (in case of crossover studies, according to Cochrane protocol (Ding et al. 2015)^[[1]](#footnote-1)^ |
| Probably Low Risk of Bias (+) | |
| n.a. | - indirect evidence that subjects were allocated to any study group, including controls, using a method with a random component   **OR**   - it is deemed that allocation without a clearly random component during the study would not appreciably bias results   **OR**   - indirect evidence that sequences of exposure were allocated using a method with a random component (in case of crossover studies) |
| Probably High Risk of Bias (-) | |
| n.a. | - indirect evidence that subjects were allocated to study groups using a method with a non-random component   **OR**   - there is insufficient information provided about how subjects were allocated to study groups (record “NR” as basis for answer)   **OR**   - indirect evidence that sequences of exposure were allocated using a method with a non-random component (in case of crossover studies |
| Definitely High Risk of Bias (--) | |
| n.a. | - direct evidence that subjects were allocated to study groups using a non-random method (e.g., according to investigator’s judgment)   **OR**   - direct evidence that sequences of exposure were allocated using a method with a non-random component (in case of crossover studies) |
| **Question 2: Was allocation to study groups adequately concealed?** | |
| Co, CaCo, CrSe | HCT |
| n.a. | *n.a. because only studies with a crossover design could be identified in the present review (i.e., studies without a separate control group)* |
| **Question 3: Did selection of study participants result in appropriate comparison groups?** | |
| Co, CaCo, CrSe | HCT |
| Definitely Low Risk of Bias (++) | |
| - **Co, CrSe:** direct evidence that subjects (both exposed and non-exposed) were similar (e.g., recruited from the same eligible population, recruited with the same method of ascertainment using the same inclusion and exclusion criteria, and had similar age and health status), recruited within the same time frame, and had the similar participation/response rates   **OR**   - **CaCo:** direct evidence that cases and controls were similar (e.g., recruited from the same eligible population, including being of similar age, gender, ethnicity, and eligibility criteria other than outcome of interest as appropriate), recruited within the same time frame, and controls are described as having no history of the outcome | n.a. |
| Probably Low Risk of Bias (+) | |
| - **Co, CrSe:** indirect evidence that subjects (both exposed and non-exposed) were similar (e.g., recruited from the same eligible population, recruited with the same method of ascertainment using the same inclusion and exclusion criteria, and were of similar age and health status), recruited within the same time frame, and had similar participation/response rates   **OR**   - differences between groups would not appreciably bias results - **CaCo:** indirect evidence that cases and controls were similar (e.g., recruited from the same eligible population, recruited with the same method of ascertainment using the same inclusion and exclusion criteria, and were of similar age), recruited within the same time frame, and controls are described as having no history of the outcome   **OR**   - differences between cases and controls would not appreciably bias results | n.a. |
| Probably High Risk of Bias (-) | |
| - **Co, CrSe:** indirect evidence that subjects (both exposed and non-exposed) were not similar, recruited within very different time frames, or had very different participation/response rates   **OR**   - there is insufficient information provided about the comparison group, including a different rate of non-response without an explanation (record “NR” as basis for answer) - **CaCo:** indirect evidence that controls were drawn from a very dissimilar population than cases or recruited within very different time frames,   **OR**   - there is insufficient information provided about the appropriateness of controls, including rate of response reported for cases only (record “NR” as basis for answer) | n.a. |
| Definitely High Risk of Bias (--) | |
| - **Co, CrSe:** direct evidence that subjects (both exposed and non-exposed) were not similar, recruited within very different time frames, or had very different participation/response rates - **CaCo:** direct evidence that controls were drawn from a very dissimilar population than cases or recruited within very different time frames | n.a. |

| **Confounding Bias** | |
| --- | --- |
| **Question 4: Did the study design or analysis account for important confounding and modifying variables?**  *Endpoints were not restricted in the present review, i.e., studies with all physiological and health-related outcomes were included. Due to this comprehensive approach, confounders were not predefined for each endpoint, as proposed by the OHAT tool. Therefore, only two RoB categories were used in this criterion.* | |
| Co, CaCo, CrSe | HCT |
| Definitely Low Risk of Bias (++) | |
| - **Co, CaCo, CrSe**: Age, sex, and additional confounders were considered   **AND**   - appropriate adjustment in the analysis | n.a. |
| Probably Low Risk of Bias (+) | |
| n.a. | n.a. |
| Probably High Risk of Bias (-) | |
| - **Co, CaCo, CrSe:** Only age and sex were considered as confounders   **OR**  no adjustment in the analysis | n.a. |
| Definitely High Risk of Bias (--) | |
| n.a. | n.a. |
| **Performance Bias** | |
| **Question 5: Were experimental conditions identical across study groups?**  *In the original OHAT protocol, this criterion only applies to animal studies. However, in human studies with exposures to EMF, systematic differences between the experimental conditions during exposure versus sham exposure could substantially bias the outcomes. Therefore, we deemed this criterion as crucial for a well-controlled design in human studies. For HCT investigating EMF, we specifically assessed the presence and quality of sham exposure in the control group(s) or conditions in this criterion.* | |
| Co, CaCo, CrSe | HCT |
| Definitely Low Risk of Bias (++) | |
| n.a. | - direct evidence that exposure-associated side effects and any other conditions were identical across study groups, i.e., a sham exposure is explicitly mentioned   **AND**   - the sham exposure apparatus was identical to the exposure apparatus   **AND**   - all potential confounders (e.g., noise, vibrations, and temperature) of the exposure were considered and, if necessary, were emulated in the sham exposure or potential confounders are counteracted   **AND**   - sham-exposed subjects are treated as exposed subjects   **AND**   - sham exposure and exposure occurred at the same time and conditions   **AND**   - any differences are small, well documented, and justified |
| Probably Low Risk of Bias (+) | |
| n.a. | - indirect evidence that exposure-associated side effects and any other conditions were identical across study groups, i.e., a sham exposure is explicitly mentioned   **AND**   - sham exposure apparatus is at least very similar to the exposure apparatus   **AND**   - exposure confounders (e.g., noise, vibrations, and temperature) were not fully considered and emulated or were not counteracted   **AND**   - sham-exposed subjects were treated as exposed subjects   **AND**   - sham exposure and exposure took place, at least, at comparable times and conditions   **AND**   - any differences are documented and justified   **OR**   - it is deemed that exposure-associated side effects did not exist or would not appreciably bias results |
| Probably High Risk of Bias (-) | |
| n.a. | - indirect evidence that exposure-associated side effects or some other conditions differed between study groups   **OR**   - a sham exposure is explicitly mentioned or there is indirect evidence for the existence of a sham exposure, but no or insufficient details are provided to assume identical conditions across study groups/conditions   **OR**   - insufficient information is provided (e.g., not reported or “NR”) |
| Definitely High Risk of Bias (--) | |
| n.a. | - direct evidence that exposure-associated side effects or some other conditions differed between study groups   **OR**   - direct evidence that there was no sham exposure   **OR**   - it is very likely that there was no sham exposure because only terms, such as “control group” or “control condition” without further explanations, are used |
| **Question 6: Were the research personnel and human subjects blinded to the study group during the study?** | |
| Co, CaCo, CrSe | HCT |
| Definitely Low Risk of Bias (++) | |
| n.a. | - direct evidence that subjects and research personnel were adequately blinded to study groups and it is unlikely that they could have broken the blinding until the full completion of the experiment performance |
| Probably Low Risk of Bias (+) | |
| n.a. | - indirect evidence that subjects and research personnel were adequately blinded to study groups and it is unlikely that they could have broken the blinding during the study   **OR**   - it is deemed that lack of adequate blinding during the study would not appreciably bias results |
| Probably High Risk of Bias (-) | |
| n.a. | - indirect evidence that it was possible for research personnel or subjects to infer the study groups   **OR**   - there is insufficient information provided about blinding to study groups during the study (record “NR” as basis for answer) |
| Definitely High Risk of Bias (--) | |
| n.a. | - direct evidence for lack of adequate blinding of the study groups, including no blinding or incomplete blinding of research personnel and subjects |

| **Attrition/Exclusion Bias** | |
| --- | --- |
| **Question 7: Were outcome data complete without attrition or exclusion from analysis?** | |
| Co, CaCo, CrSe | HCT |
| Definitely Low Risk of Bias (++) | |
| - **Co:** direct evidence that loss of subjects (i.e., incomplete outcome data) was adequately addressed and reasons were documented when subjects were removed from a study - **CaCo, CrSe:** direct evidence that exclusion of subjects from analyses was adequately addressed and reasons were documented when subjects were removed from a study | - direct evidence that there was no loss of subjects during the study and outcome data were complete   **OR**   - loss of subjects (i.e., incomplete outcome data) was adequately addressed and reasons were documented when human subjects were removed from a study |
| Probably Low Risk of Bias (+) | |
| - **Co:** There is indirect evidence that loss of subjects (i.e., incomplete outcome data) was adequately addressed and reasons were documented when human subjects were removed from a study   **OR**   - it is deemed that the proportion lost to follow-up would not appreciably bias results - **CaCo, CrSe:** indirect evidence that exclusion of subjects from analyses was adequately addressed and reasons were documented when subjects were removed from a study | - indirect evidence that loss of subjects (i.e., incomplete outcome data) was adequately addressed and reasons were documented when subjects were removed from a study   **OR**   - it is deemed that the proportion lost to follow-up would not appreciably bias results |
| Probably High Risk of Bias (-) | |
| - **Co:** indirect evidence that loss of subjects (i.e., incomplete outcome data) was unacceptably large and not adequately addressed   **OR**   - there is insufficient information provided about numbers of subjects lost to follow-up (record “NR” as basis for answer) - **CaCo, CrSe:** indirect evidence that exclusion of subjects from analyses was not adequately addressed   **OR**   - there is insufficient information provided about why subjects were removed from the study (record “NR” as basis for answer) | - indirect evidence that loss of subjects (i.e., incomplete outcome data) was unacceptably large (greater than 20% in each group) and not adequately addressed   **OR**   - there is insufficient information provided about numbers of subjects lost to follow-up (record “NR” as basis for answer). |
| Definitely High Risk of Bias (--) | |
| - **Co:** direct evidence that loss of subjects (i.e., incomplete outcome data) was unacceptably large and not adequately addressed - **CaCo, CrSe:** direct evidence that exclusion of subjects from analyses was not adequately addressed | - direct evidence that loss of subjects (i.e., incomplete outcome data) was unacceptably large and not adequately addressed |
| **Detection Bias** | |
| **Question 8: Can we be confident in the exposure characterization?**  *In this criterion, we specifically assessed the quality of the RF EMF exposure characterization. The following basic pieces of information were required for HCT: frequency, modulation scheme, description of the signal source and exposure set-up, description of measurement devices and probes, method of dosimetry (measurement, calculation, simulation), field strength, and exposure duration.* | |
| Co, CaCo, CrSe | HCT |
| Definitely Low Risk of Bias (++) | |
| - direct evidence that exposure was consistently assessed (i.e., under the same method and time frame) using well-established methods that directly measure exposure (e.g., comprehensive measurements or use of personal dosimeters and/or operator data) | - direct evidence that the exposure was adequately characterized   **AND**   - consistently administered (i.e., with the same method and time frame) across study groups |
| Probably Low Risk of Bias (+) | |
| - indirect evidence that the exposure was consistently assessed using well-established methods that directly measure exposure   **OR**   - exposure was assessed using indirect measures (e.g., questionnaire that have been validated)   **OR**   - exposure modeling was performed | - indirect evidence that the exposure was adequately characterized   **AND**   - consistently administered (i.e., with the same method and time frame) across study groups |
| Probably High Risk of Bias (-) | |
| - indirect evidence that the exposure was assessed using poorly validated methods that directly measure exposure   **OR**   - direct evidence that the exposure was assessed using indirect measures that have not been validated or empirically shown to be consistent with methods that directly measure exposure (e.g., self-reported questionnaires without validation)   **OR**   - there is insufficient information provided about the exposure assessment, including validity and reliability, but no evidence for concern about the method used (record “NR” as basis for answer) | - indirect evidence that the exposure was assessed using poorly validated methods   **OR**   - many important parameters lacking   **OR**   - there is insufficient information provided about the validity of the exposure assessment method, but no evidence for concern (record “NR” as basis for answer) |
| Definitely High Risk of Bias (--) | |
| - direct evidence that the exposure was assessed using methods with poor validity   **OR**   - evidence of exposure misclassification | - direct evidence that the exposure was assessed using poorly validated methods   **OR**   - most important parameters are lacking |
| **Question 9: Can we be confident in the outcome assessment?** | |
| CaCo, CrSe | HCT, Co |
| Definitely Low Risk of Bias (++) | |
| - **CaCo:** direct evidence that the outcome was assessed in cases (i.e., case definition) and controls using acceptable methods   **AND**   - subjects had been followed-up for the same length of time in all study groups   **AND**   - direct evidence that the outcome assessors (including study subjects, if outcomes were self-reported) were adequately blinded to the exposure level when outcome was assessed in cases (i.e., case definition) and controls - **CrSe:** direct evidence that the outcome was assessed using well-established methods   **AND**   - outcome assessors (including study subjects, if outcomes were self-reported) were adequately blinded to the exposure level and it is unlikely that they could have broken the blinding prior to reporting outcomes | - direct evidence that the outcome was assessed using well-established methods   **AND**   - subjects had been followed-up for the same length of time in all study groups **AND** - the outcome assessors (including study subjects, if outcomes were self-reported) were adequately blinded to the study groups, and it is unlikely that they could have broken the blinding prior to assessing outcomes |
| Probably Low Risk of Bias (+) | |
| - **CaCo:** indirect evidence that the outcome was assessed in cases (i.e., case definition) and controls using acceptable methods   **AND**   - subjects had been followed for the same length of time in all study groups   **OR**   - it is deemed that the outcome assessment methods used would not appreciably bias results   **AND**   - direct evidence that the outcome assessors were adequately blinded to the exposure level when reporting outcomes   **OR**   - it is deemed that lack of adequate blinding of outcome assessors would not appreciably bias results (including that subjects self-reporting outcomes were likely not aware of reported links between the exposure and that outcome or lack of blinding is unlikely to bias a particular outcome). - **CrSe:** indirect evidence that the outcome was assessed using acceptable methods   **OR**   - it is deemed that the outcome assessment methods used would not appreciably bias results   **AND**   - indirect evidence that the outcome assessors were adequately blinded to the exposure level and it is unlikely that they could have broken the blinding prior to reporting outcomes,   **OR**   - it is deemed that lack of adequate blinding of outcome assessors would not appreciably bias results (including that subjects self-reporting outcomes were likely not aware of reported links between the exposure and that outcome lack of blinding is unlikely to bias a particular outcome) | - indirect evidence that the outcome was assessed using acceptable methods   **AND**   - subjects had been followed for the same length of time in all study groups   **OR**   - it is deemed that the outcome assessment methods used would not appreciably bias results   **AND**  there is indirect evidence that the outcome assessors, including study subjects, if outcomes were self-reported) were adequately blinded to the study group, and it is unlikely that they could have broken the blinding prior to assessing outcomes  **OR**  it is deemed that lack of adequate blinding of outcome assessors would not appreciably bias results, which is more likely to apply to objective outcome measures |
| Probably High Risk of Bias (-) | |
| - **CaCo**: indirect evidence that the outcome was assessed in cases (i.e., case definition) using an insensitive instrument   **OR**   - there is insufficient information provided about how cases were identified (record “NR” as basis for answer)   **OR**   - indirect evidence that it was possible for outcome assessors to infer the exposure level prior to reporting outcomes (including that subjects self-reporting outcomes were likely aware of reported links between the exposure and outcome)   **OR**   - there is insufficient information provided about blinding of outcome assessors (record “NR” as basis for answer) - **CrSe:** indirect evidence that the outcome assessment method is an insensitive instrument   **OR**   - indirect evidence that it was possible for outcome assessors to infer the exposure level prior to reporting outcomes (including that subjects self-reporting outcomes were likely aware of reported links between the exposure and outcome)   **OR**   - there is insufficient information provided about blinding of outcome assessors (record “NR” as basis for answer) | - indirect evidence that the outcome assessment method is an insensitive instrument   **OR**   - the length of follow-up differed by study group   **OR**   - it was possible for outcome assessors (including study subjects if outcomes were self-reported) to infer the study group prior to reporting outcomes   **OR**   - there is insufficient information provided about blinding of outcome assessors (record “NR” as basis for answer). |
| Definitely High Risk of Bias (--) | |
| - **CaCo:** direct evidence that the outcome was assessed in cases (i.e., case definition) using an insensitive instrument   **OR**   - direct evidence that outcome assessors were aware of the exposure level prior to reporting outcomes (including that subjects self-reporting outcomes were aware of reported links between the exposure and outcome) - **CrSe:** direct evidence that the outcome assessment method is an insensitive instrument   **OR**   - direct evidence that outcome assessors were aware of the exposure level prior to reporting outcomes (including that subjects self-reporting outcomes were aware of reported links between the exposure and outcome) | - direct evidence that the outcome assessment method is an insensitive instrument   **OR**   - the length of follow-up differed by study group   **OR**   - direct evidence for lack of adequate blinding of outcome assessors (including study subjects if outcomes were self-reported), including no blinding or incomplete blinding |
| **Selective Reporting Bias** | |
| **Question 10: Were all measured outcomes reported?** | |
| Co, CaCo, CrSe, HCT | |
| Definitely Low Risk of Bias (++) | |
| - direct evidence that all of the study’s measured endpoints (primary and secondary) outlined in the protocol, methods, abstract, and/or introduction (that are relevant for the evaluation) have been reported | |
| Probably Low Risk of Bias (+) | |
| - indirect evidence that all of the study’s measured endpoints (primary and secondary) outlined in the protocol, methods, abstract, and/or introduction (that are relevant for the evaluation) have been reported   **OR**   - analyses that had not been planned in advance are clearly indicated and it is deemed that the unplanned analyses were appropriate and that selective reporting would not appreciably bias results. This would include outcomes reported with insufficient detail, such as reporting only results that were statistically significant (or not) | |
| Probably High Risk of Bias (-) | |
| - indirect evidence that not all of the study’s measured endpoints (primary and secondary) outlined in the protocol, methods, abstract, and/or introduction (that are relevant for the evaluation) have been reported   **OR**   - indirect evidence that unplanned analyses were included, which may appreciably bias results   **OR**   - there is insufficient information provided about selective outcome reporting (record “NR” as basis for answer) | |
| Definitely High Risk of Bias (--) | |
| - direct evidence that not all of the study’s measured endpoints (primary and secondary) outlined in the protocol, methods, abstract, and/or introduction (that are relevant for the evaluation) have been reported. In addition to not reporting outcomes, this would include reporting outcomes based on composite score without individual outcome components or outcomes reported using measurements, analysis methods or subsets of the data (e.g., subscales) that were not pre-specified or reporting outcomes not pre-specified, or that unplanned analyses were included, which would appreciably bias results | |
| **Other Bias** | |
| **Question 11: Were there any other potential threats to internal validity?** | |
| Co, CaCo, CrSe, HCT | |
| Definitely Low Risk of Bias (++) | |
| - direct evidence that appropriate statistics were performed   **AND**   - direct evidence that researchers adhered to the study protocol   **AND**   - direct evidence that the study design accounts for important confounding variables in HCT (if applicable) | |
| Probably Low Risk of Bias (+) | |
| - indirect evidence that appropriate statistics were performed   **AND**   - indirect evidence that researchers adhered to the study protocol   **AND**   - indirect evidence that the study design accounts for important confounding variables in HCT (if applicable) | |
| Probably High Risk of Bias (-) | |
| - indirect evidence that inappropriate statistics were performed   **OR**   - there is insufficient information provided about appropriateness of statistical methods (record “NR” as basis for answer)   **OR**   - indirect evidence that researchers did not adhere to the study protocol   **OR**   - indirect evidence that there were any other potential threats to internal validity (e.g., important confounding variables in HCT) | |
| Definitely High Risk of Bias (--) | |
| - direct evidence that inappropriate statistics were performed OR no statistical analysis was performed   **OR**   - direct evidence that researchers did not adhere to the study protocol   **OR**   - direct evidence that there were any other potential threats to internal validity (e.g., important confounding variables in HCT) | |

1. Ding H, Hu GL, Zheng XY, Chen Q, Threapleton DE, et al. (2015) The Method Quality of Cross-Over Studies Involved in Cochrane Systematic Reviews. PLOS ONE 10(4): e0120519. https://doi.org/10.1371/journal.pone.0120519 [↑](#footnote-ref-1)
