# Supplementary material for: Systematic review of the physiological and health-related effects of radiofrequency electromagnetic field exposure from wireless communication devices on children and adolescents in experimental and epidemiological human studies
Source: PLoS One. 2022 Jun 1;17(6):e0268641. doi: 10.1371/journal.pone.0268641 (PMC9159629; doi:10.1371/journal.pone.0268641)
Supplement: S2 Table — (DOCX) [file pone.0268641.s005.docx]

**S2 Table. All excluded articles including bibliographic data and the reasons for their exclusion after checking for eligibility.**

| **Number** | **Reference** | **Summary comment for exclusion** |
| --- | --- | --- |
|  | Abi-Jaoude E, Naylor KT, Pignatiello A (2020): Smartphones, social media use and youth mental health. CMAJ 192 (6): E136-E141; doi: 10.1503/cmaj.190434 | Not EMF/health-related |
|  | Al-Mohtaseb Z, Schachter S, Shen Lee B, Garlich J, Trattler W (2021): The Relationship Between Dry Eye Disease and Digital Screen Use. Clin Ophthalmol 15: 3811-3820; doi: 10.2147/OPTH.S321591 | Not EMF/health-related |
|  | Almuaigel D, Alanazi A, Almuaigel M, Alshamrani F, AlSheikh M, Almuhana N, Zeeshan M, Alshurem M, Alshammari A, Mansi K (2021): Impact of Technology Use on Behavior and Sleep Scores in Preschool Children in Saudi Arabia. Front Psychiatry 12: 649095; doi: 10.3389/fpsyt.2021.649095 | Not EMF/health-related |
|  | Alvarez-Peregrina C, Sánchez-Tena MÁ, Martinez-Perez C, Villa-Collar C (2020): The Relationship Between Screen and Outdoor Time With Rates of Myopia in Spanish Children. Front Public Health 8: 560378; doi: 10.3389/fpubh.2020.560378 | Not EMF/health-related |
|  | Ashrafinia F, Moeindarbari S, Razmjouei P, Ghazanfarpour M, Najafi MN, Ghalibaf AAM, Abdi F (2021): Can Prenatal and Postnatal Cell Phone Exposure Increase Adverse Maternal, Infant and Child Outcomes? Rev Bras Ginecol Obstet.43 (11): 870-877; doi: 10.1055/s-0041-1736173 | Type of study not relevant (review) |
|  | Aydin D, Feychting M, Schüz J, Tynes T, Andersen TV, Schmidt LS, Poulsen AH, Johansen C, Prochazka M, Lannering B, Klaeboe L, Eggen T, Jenni D, Grotzer M, Von der Weid N, Kuehni CE, Röösli M (2011): Mobile phone use and brain tumors in children and adolescents: a multicenter case-control study. J Natl Cancer Inst 103 (16): 1264-1276; doi:10.1093/jnci/djr244 | Exposed or investigated age groups not relevant or unclear |
|  | Bamdad K, Adel Z, Esmaeili M (2019): Complications of nonionizing radiofrequency on divided attention. J Cell Biochem 120 (6): 10572-10575; doi:10.1002/jcb.28343 | Exposure condition unclear |
|  | Barchana M, Margaliot M, Liphshitz I (2012): Changes in brain glioma incidence and laterality correlates with use of mobile phones - a nationwide population based study in Israel. Asian Pac J Cancer Prev 13 (11): 5857-5863; doi:10.7314/apjcp.2012.13.11.5857 | Exposed or investigated age groups not relevant or unclear |
|  | Baste V, Oftedal G, Mollerlokken OJ, Mild KH, Moen BE (2015): Prospective study of pregnancy outcomes after parental cell phone exposure: the Norwegian Mother and Child Cohort Study. Epidemiology 26 (4): 613-621; doi:10.1097/EDE.0000000000000293 | Study on pregnancy outcomes/effects on fetus |
|  | Bektas H, Bektas MS, Dasdag S (2018): Effects of mobile phone exposure on biochemical parameters of cord blood: A preliminary study. Electromagn Biol Med 37 (4): 184-191; doi:10.1080/15368378.2018.1499033 | Study on pregnancy outcomes/effects on fetus |
|  | Bellagamba F, Presaghi F, Di Marco M, D'Abundo E, Blanchfield O, Barr R (2021): How Infant and Toddlers' Media Use Is Related to Sleeping Habits in Everyday Life in Italy. Front Psychol 12: 589664; doi: 10.3389/fpsyg.2021.589664 | Not EMF/health-related |
|  | Berg-Beckhoff G, Blettner M, Kowall B, Breckenkamp J, Schlehofer B, Schmiedel S, Bornkessel C, Reis U, Potthoff P, Schüz J (2009): Mobile phone base stations and adverse health effects: phase 2 of a cross-sectional study with measured radio frequency electromagnetic fields. Occup Environ Med 66 (2): 124-130; doi:10.1136/oem.2008.039834 | Exposed or investigated age groups not relevant or unclear |
|  | Besset D, Selmaoui B, Delanaud S, de Seze R, Leke A, Stéphan-Blanchard E (2021): Individual Exposure to Environmental Radiofrequency Electromagnetic Fields in Hospitalized Preterm Neonates. Bioelectromagnetics 2021; 42 (5): 432-434 | Not EMF/health-related |
|  | Bhargav H, Manjunath NK, Varambally S, Mooventhan A, Bista S, Singh D, Chhabra H, Venkatasubramanian G, Srinivasan TM, Nagendra HR (2016): Acute effects of 3G mobile phone radiations on frontal haemodynamics during a cognitive task in teenagers and possible protective value of Om chanting. Int Rev Psychiatry 28 (3): 288-298; doi:10.1080/09540261.2016.1188784 | Exposed or investigated age groups not relevant or unclear |
|  | Bhargav H, Srinivasan TM, Bista S, Mooventhan A, Suresh V, Hankey A, Nagendra HR (2017): Acute effects of mobile phone radiations on subtle energy levels of teenagers using electrophotonic imaging technique: A randomized controlled study. Int J Yoga 10 (1): 16-23; doi:10.4103/0973-6131.186163 | Exposed or investigated age groups not relevant or unclear |
|  | Blettner M, Schlehofer B, Breckenkamp J, Kowall B, Schmiedel S, Reis U, Potthoff P, Schüz J, Berg G (2009): Mobile phone base stations and adverse health effects: phase 1 of a population-based, cross-sectional study in Germany. Occup Environ Med 66 (2): 118-123; doi:10.1136/oem.2007.037721 | Exposed or investigated age groups not relevant or unclear |
|  | Böhler E, Schüz J (2004): Cellular telephone use among primary school children in Germany. Eur J Epidemiol 19 (11): 1043-1050; doi:10.1007/s10654-004-2174-x | Not EMF/health-related |
|  | Boileau N, Margueritte F, Gauthier T, Boukeffa N, Preux PM, Labrunie A, Aubard Y (2020): Mobile phone use during pregnancy: which association with fetal growth? J Gynecol Obstet Hum Reprod 2020; 49: 101852 | Study on pregnancy outcomes/effects on fetus |
|  | Bolte JFB, Clahsen S, Vercruijsse W, Houtveen JH, Schipper CMA, van Kamp I, Bogers R (2019): Ecological momentary assessment study of exposure to radiofrequency electromagnetic fields and non-specific physical symptoms with self-declared electrosensitives. Environ Int 131: 104948; doi:10.1016/j.envint.2019.104948 | Exposed or investigated age groups not relevant or unclear |
|  | Buchner K, Eger H (2011): Veränderung klinisch bedeutsamer Neurotransmitter unter dem Einfluss modulierter hochfrequenter Felder - Eine Langzeiterhebung unter lebensnahen Bedingungen. Umwelt-Medizin-Gesellschaft 24 (1): 44-57 | Exposed or investigated age groups not relevant or unclear |
|  | Bunin GR, Ward E, Kramer S, Rhee CA, Meadows AT (1990): Neuroblastoma and parental occupation. Am J Epidemiol 131 (5): 776-780; doi:10.1023/A:1008998925889 | No exposure to mobile communications |
|  | Burnell K, George MJ, Jensen M, Hoyle RH, Odgers CL (2021): Associations Between Adolescents' Daily Digital Technology Use and Sleep. J Adolesc Health 29: S1054-139X(21)00492-4; doi: 10.1016/j.jadohealth.2021.09.033 | Not EMF/health-related |
|  | Cabré-Riera A, Torrent M, Donaire-Gonzalez D, Vrijheid M, Cardis E, Guxens M (2019): Telecommunication devices use, screen time and sleep in adolescents. Environ Res 171: 341-347; doi:10.1016/j.envres.2018.10.036 | Exposed or investigated age groups not relevant or unclear |
|  | Calvente I, Perez-Lobato R, Nunez MI, Ramos R, Guxens M, Villalba J, Olea N, Fernandez MF (2016): Does exposure to environmental radiofrequency electromagnetic fields cause cognitive and behavioral effects in 10-year-old boys? Bioelectromagnetics 37 (1): 25-36; doi:10.1002/bem.21951 | No exposure to mobile communications |
|  | Carlberg M, Hedendahl L, Ahonen M, Koppel T, Hardell L (2016): Increasing incidence of thyroid cancer in the Nordic countries with main focus on Swedish data. BMC Cancer 16: 426; doi:10.1186/s12885-016-2429-4 | No exposure assessment for the individual |
|  | Caumo GH, Spritzer D, Carissimi A, Tonon AC (2020): Exposure to electronic devices and sleep quality in adolescents: A matter of type, duration, and timing. Sleep Health 6 (2): 172-178; doi:10.1016/j.sleh.2019.12.004 | Not EMF/health-related |
|  | Celik O, Hascalik S (2004): Effect of electromagnetic field emitted by cellular phones on fetal heart rate patterns. Eur J Obstet Gynecol Reprod Biol 112 (1): 55-56; doi: 10.1016/s0301-2115(03)00288-4 | Study on pregnancy outcomes/effects on fetus |
|  | Chahine R, Farah R, Chahoud M, Harb A, Tarabay R, Sauleau E, Godbout R (2018): Assessing sleep quality of Lebanese high school students in relation to lifestyle: pilot study in Beirut. East Mediterr Health J 24 (8): 722-728; doi: 10.26719/2018.24.8.722 | Not EMF/health-related |
|  | Chang HY, Park EJ, Yoo HJ, Lee JW, Shin Y (2018): Electronic Media Exposure and Use among Toddlers. Psychiatry Investig 15 (6): 568-573; doi: 10.30773/pi.2017.11.30.2 | Not EMF/health-related |
|  | Charlton A, Bates C (2000): Decline in teenage smoking with rise in mobile phone ownership: hypothesis. BMJ 321 (7269): 1155 | Exposed or investigated age groups not relevant or unclear |
|  | Chen R, Liu J, Cao X, Duan S, Wen S, Zhang S, Xu J, Lin L, Xue Z, Lu J (2020): The relationship between mobile phone use and suicide-related behaviors among adolescents: The mediating role of depression and interpersonal problems. J Affect Disord 269: 101-107; doi: 10.1016/j.jad.2020.01.128 | Not EMF/health-related |
|  | Chetty-Mhlanga S, Fuhrimann S, Eeftens M, Basera W, Hartinger S, Dalvie MA, Röösli M (2020): Different aspects of electronic media use, symptoms and neurocognitive outcomes of children and adolescents in the rural Western Cape region of South Africa. Environ Res 184: 109315; doi: 10.1016/j.envres.2020.109315 | Not EMF/health-related |
|  | Chia SE, Chia HP, Tan JS (2000): Prevalence of headache among handheld cellular telephone users in Singapore: a community study. Environ Health Perspect 108 (11): 1059-1062; doi:10.1289/ehp.001081059 | Exposed or investigated age groups not relevant or unclear |
|  | Chindamo S, Buja A, DeBattisti E, Terraneo A, Marini E, Gomez Perez LJ, Marconi L, Baldo V, Chiamenti G, Doria M, Ceschin F, Malorgio E, Tommasi M, Sperotto M, Buzzetti R, Gallimberti L (2019): Sleep and new media usage in toddlers. Eur J Pediatr 178 (4): 483-490; doi: 10.1007/s00431-019-03318-7 | Not EMF/health-related |
|  | Chongchitpaisan W, Wiwatanadate P, Tanprawate S, Narkpongphan A, Siripon N (2021): Trigger of a migraine headache among Thai adolescents smartphone users: a time series study. Environ Anal Health Toxicol 36 (1): e2021006-0; doi: 10.5620/eaht.2021006 | Exposed or investigated age groups not relevant or unclear |
|  | Cinquetti M, Biasin M, Ventimiglia M, Balanzoni L, Signorelli D, Pietrobelli A (2021): Functional gastrointestinal disorders and smartphone use in adolescents. Clin Exp Pediatr 64 (9): 494-496; doi: 10.3345/cep.2020.01326 | Not EMF/health-related |
|  | Col-Araz N (2013): Evaluation of factors affecting birth weight and preterm birth in southern Turkey. J Pak Med Assoc 63 (4): 459-462 | Study on pregnancy outcomes/effects on fetus |
|  | de Vocht F, Burstyn I, Cherrie JW (2011): Time trends (1998-2007) in brain cancer incidence rates in relation to mobile phone use in England. Bioelectromagnetics 32 (5): 334-339; doi:10.1002/bem.20648 | No exposure assessment for the individual |
|  | De-Hita-Cantalejo C, García-Pérez Á, Sánchez-González JM, Capote-Puente R, Sánchez-González MC (2021): Accommodative and binocular disorders in preteens with computer vision syndrome: a cross-sectional study. Ann N Y Acad Sci 1492 (1): 73-81; doi: 10.1111/nyas.14553 | Not EMF/health-related |
|  | Do CW, Chan LYL, Tse ACY, Cheung T, So BCL, Tang WC, Yu WY, Chu GCH, Szeto GPY, Lee RLT, Lee PH. Association between Time Spent on Smart Devices and Change in Refractive Error: A 1-Year Prospective Observational Study among Hong Kong Children and Adolescents. Int J Environ Res Public Health. 2020 Nov 30;17(23):8923. doi: 10.3390/ijerph17238923 | Not EMF/health-related |
|  | Dode AC, Leao MM, Tejo FD, Gomes AC, Dode DC, Dode MC, Moreira CW, Condessa VA, Albinatti C, Caiaffa WT (2011): Mortality by neoplasia and cellular telephone base stations in the Belo Horizonte municipality, Minas Gerais state, Brazil. Sci Total Environ 409 (19): 3649-3665; doi:10.1016/j.scitotenv.2011.05.051 | Exposed or investigated age groups not relevant or unclear |
|  | Dolk H, Elliott P, Shaddick G, Walls P, Thakrar B (1997): Cancer incidence near radio and television transmitters in Great Britain. II. All high power transmitters. Am J Epidemiol 145 (1): 10-17; doi:10.1093/oxfordjournals.aje.a009026 | No exposure to mobile communications |
|  | Dolk H, Shaddick G, Walls P, Grundy C, Thakrar B, Kleinschmidt I, Elliott P (1997): Cancer incidence near radio and television transmitters in Great Britain. I. Sutton Coldfield transmitter. Am J Epidemiol 145 (1): 1-9 | No exposure to mobile communications |
|  | Dreyer NA, Loughlin JE, Rothman KJ (1999): Cause-specific mortality in cellular telephone users. JAMA 282 (19): 1814-1816; doi:10.1001/jama.282.19.1814-a | Exposed or investigated age groups not relevant or unclear |
|  | Duan Y, Zhang HZ, Bu RF (2011): Correlation between cellular phone use and epithelial parotid gland malignancies. Int J Oral Maxillofac Surg 40 (9): 966-972; doi:10.1016/j.ijom.2011.03.007 | Exposed or investigated age groups not relevant or unclear |
|  | Dube N, Khan K, Loehr S, Chu Y, Veugelers P (2017): The use of entertainment and communication technologies before sleep could affect sleep and weight status: a population-based study among children. Int J Behav Nutr Phys Act 14 (1): 97; doi: 10.1186/s12966-017-0547-2 | Not EMF/health-related |
|  | Eger H, Neppe F (2009): Krebsinzidenz von Anwohnern im Umkreis einer Mobilfunksendeanlage in Westfalen - Interview-basierte Piloterhebung und Risikoschätzung Umwelt-Medizin-Gesellschaft 22 (1): 55-60 | Exposed or investigated age groups not relevant or unclear |
|  | Fang WT, Ng E, Liu SM, Chiang YT, Chang MC (2021): Determinants of pro-environmental behavior among excessive smartphone usage children and moderate smartphone usage children in Taiwan. PeerJ 9: e11635; doi: 10.7717/peerj.11635 | Not EMF/health-related |
|  | Feltbower RG, Fleming SJ, Picton SV, Alston RD, Morgan D, Achilles J, McKinney PA, Birch JM (2014): UK case control study of brain tumours in children, teenagers and young adults: a pilot study. BMC Res Notes 7: 14; doi:10.1186/1756-0500-7-14 | Exposed or investigated age groups not relevant or unclear |
|  | Fuller C, Lehman E, Hicks S, Novick MB (2017): Bedtime Use of Technology and Associated Sleep Problems in Children. Glob Pediatr Health 4: 2333794X17736972; doi: 10.1177/2333794X17736972 | Not EMF/health-related |
|  | Geronikolou SA, Chamakou A, Mantzou A, Chrousos G, Kanaka-Gantenbein C (2015): Frequent cellular phone use modifies hypothalamic-pituitary-adrenal axis response to a cellular phone call after mental stress in healthy children and adolescents: A pilot study. Sci Total Environ 536: 182-188; doi:10.1016/j.scitotenv.2015.07.052 | No control group or condition |
|  | Golshevsky DM, Magnussen C, Juonala M, Kao KT, Harcourt BE, Sabin MA (2020): Time spent watching television impacts on body mass index in youth with obesity, but only in those with shortest sleep duration. J Paediatr Child Health 56 (5): 721-726; doi: 10.1111/jpc.14711 | Not EMF/health-related |
|  | Guyon PW Jr, Corroon J, Ferran K, Hollenbach K, Nguyen M (2020): Hold the Phone! Cell Phone-Related Injuries in Children, Teens, and Young Adults Are On the Rise. Glob Pediatr Health 7: 2333794X20968459; doi: 10.1177/2333794X20968459 | Not EMF/health-related |
|  | Ha M, Im H, Lee M, Kim HJ, Kim BC, Gimm YM, Pack JK (2007): Radio-frequency radiation exposure from AM radio transmitters and childhood leukemia and brain cancer. Am J Epidemiol 166 (3): 270-279; doi:10.1093/aje/kwm083 | No exposure to mobile communications |
|  | Hardell L, Carlberg M, Hansson Mild K (2010): Mobile Phone Use and the Risk for Malignant Brain Tumors: A Case-Control Study on Deceased Cases and Controls. Neuroepidemiology 35 (2): 109-114; doi:10.1159/000311044 | Exposed or investigated age groups not relevant or unclear |
|  | Hardell L, Carlberg M, Hansson Mild K, Eriksson M (2011): Case-control study on the use of mobile and cordless phones and the risk for malignant melanoma in the head and neck region. Pathophysiology 18 (4): 325-333; doi:10.1016/j.pathophys.2011.06.001 | Exposed or investigated age groups not relevant or unclear |
|  | Hocking B, Gordon I (2003): Decreased survival for childhood leukemia in proximity to television towers. Arch Environ Health 58 (9): 560-564; doi:10.3200/AEOH.58.9.560-564 | No exposure to mobile communications |
|  | Hocking B, Gordon IR, Grain HL, Hatfield GE (1996): Cancer incidence and mortality and proximity to TV towers. Med J Aust 165 (11-12): 601-605; doi:10.5694/j.1326-5377.1996.tb138661.x | No exposure to mobile communications |
|  | Hofferth SL, Moon UJ (2012): Cell Phone Use and Child and Adolescent Reading Proficiency. Psychol Pop Media Cult 1 (2): 108-122; doi: 10.1037/a0027880 | Not EMF/health-related |
|  | Hoffmann W, Terschueren C, Heimpel H, Feller A, Butte W, Hostrup O, Richardson D, Greiser E (2008): Population-based research on occupational and environmental factors for leukemia and non-Hodgkin's lymphoma: the Northern Germany Leukemia and Lymphoma Study (NLL). Am J Ind Med 51 (4): 246-257; doi:10.1002/ajim.20551 | Exposed or investigated age groups not relevant or unclear |
|  | Hosokawa R, Katsura T (2018): Association between mobile technology use and child adjustment in early elementary school age. PLoS One 13 (7): e0199959; doi: 10.1371/journal.pone.0199959 | Not EMF/health-related |
|  | Hwang Y, Kang S, Kim M, Kim TS, Seo J, Ahn H, Yoon S, Yun JP, Lee YL, Ham H, Yu HG, Park SK (2016): Association between Exposure to Smartphones and Ocular Health in Adolescents. Ophthalmic Epidemiol 23 (4): 269-276; doi:10.3109/09286586.2015.1136652 | Exposed or investigated age groups not relevant or unclear |
|  | Ikeda K, Nakamura K (2014): Association between mobile phone use and depressed mood in Japanese adolescents: a cross-sectional study. Environ Health Prev Med 19 (3): 187-93. doi: 10.1007/s12199-013-0373-3 | Not EMF/health-related |
|  | Inskip PD, Hoover RN, Devesa SS (2010): Brain cancer incidence trends in relation to cellular telephone use in the United States. Neuro Oncol 12 (11): 1147-1151; doi:10.1093/neuonc/noq077 | No exposure assessment for the individual |
|  | Johansson AE, Petrisko MA, Chasens ER (2016): Adolescent Sleep and the Impact of Technology Use Before Sleep on Daytime Function. J Pediatr Nurs 31 (5): 498-504; doi: 10.1016/j.pedn.2016.04.004 | Not EMF/health-related |
|  | Kanal E, Gillen J, Evans JA, Savitz DA, Shellock FG (1993): Survey of reproductive health among female MR workers. Radiology 187 (2): 395-399; doi:10.1148/radiology.187.2.8475280 | No exposure to mobile communications |
|  | Kashif K, Tariq R, Ayesha, Hussain A, Shahid M (2019): Effects of smartphone usage on psychological wellbeing of school going children in Lahore, Pakistan. J Pak Med Assoc 69 (7): 955-958 | Not EMF/health-related |
|  | Kazancı B, Eroglu FC (2021): The Effects of Daily Digital Device Use on the Ocular Surface in Healthy Children. Optom Vis Sci; doi: 10.1097/OPX.0000000000001840. | Not EMF/health-related |
|  | Khaki-Khatibi F, Nourazarian A, Ahmadi F, Farhoudi M, Savadi-Oskouei D, Pourostadi M, Asgharzadeh M (2019): Relationship between the use of electronic devices and susceptibility to multiple sclerosis. Cogn Neurodyn 13 (3): 287-292; doi: 10.1007/s11571-019-09524-1 | Exposed or investigated age groups not relevant or unclear |
|  | Kim H, Cho MK, Ko H, Yoo JE, Song YM (2020): Association between Smartphone Usage and Mental Health in South Korean Adolescents: The 2017 Korea Youth Risk Behavior Web-Based Survey. Korean J Fam Med 41 (2): 98-104; doi: 10.4082/kjfm.18.0108 | Not EMF/health-related |
|  | Kim HN, Kwon YB, Byon MJ, Kim JB (2020): Injury Prevention, Safety Education and Violence in Relation to the Risk of Tooth Fracture among Korean Adolescents. Int J Environ Res Public Health 17 (22): 8556; doi: 10.3390/ijerph17228556 | Not EMF/health-related |
|  | Kim MH, Min S, Ahn JS, An C, Lee J (2019): Association between high adolescent smartphone use and academic impairment, conflicts with family members or friends, and suicide attempts. PLoS One 14 (7): e0219831; doi: 10.1371/journal.pone.0219831 | Not EMF/health-related |
|  | Kim SJH, Ioannides SJ, Elwood JM (2015): Trends in incidence of primary brain cancer in New Zealand, 1995 to 2010. Aust N Z J Public Health 39 (2): 148-152; doi:10.1111/1753-6405.12338 | No exposure assessment for the individual |
|  | Kolodynski AA, Kolodynska VV (1996): Motor and psychological functions of school children living in the area of the Skrunda Radio Location Station in Latvia. Sci Total Environ 180 (1): 87-93; doi:10.1016/0048-9697(95)04924-X | No exposure to mobile communications |
|  | Kramarenko AV, Tan U (2003): Effects of high-frequency electromagnetic fields on human EEG: a brain mapping study. Int J Neurosci 113 (7): 1007-1019; doi:10.1080/00207450390220330 | Exposure condition unclear |
|  | Kücer N, Pamukcu T (2014): Self-reported symptoms associated with exposure to electromagnetic fields: a questionnaire study. Electromagn Biol Med 33 (1): 15-17; doi:10.3109/15368378.2013.783847 | Exposed or investigated age groups not relevant or unclear |
|  | Lajunen HR, Keski-Rahkonen A, Pulkkinen L, Rose RJ, Rissanen A, Kaprio J (2007): Are computer and cell phone use associated with body mass index and overweight? A population study among twin adolescents. BMC Public Health 7: 24; doi: 10.1186/1471-2458-7-24 | Not EMF/health-related |
|  | Larsen AI, Olsen J, Svane O (1991): Gender-specific reproductive outcome and exposure to high-frequency electromagnetic radiation among physiotherapists. Scand J Work Environ Health 17 (5): 324-329; doi:10.5271/sjweh.1695 | No exposure to mobile communications |
|  | Larsen AI (1991): Congenital malformations and exposure to high-frequency electromagnetic radiation among Danish physiotherapists. Scand J Work Environ Health 17 (5): 318-323; doi:10.5271/sjweh.1696 | No exposure to mobile communications |
|  | Leitgeb N, Tropper K (1993): Augen-Erwärmung, verursacht durch Mikrowellen-Öfen. Biomed Tech (Berl) 38 (1-2): 17-20 | No exposure to mobile communications |
|  | Lerman Y, Jacubovich R, Green MS (2001): Pregnancy outcome following exposure to shortwaves among female physiotherapists in Israel. Am J Ind Med 39 (5): 499-504; doi:10.1002/ajim.1043 | No exposure to mobile communications |
|  | Li CY, Liu CC, Chang YH, Chou LP, Ko MC (2012): A population-based case-control study of radiofrequency exposure in relation to childhood neoplasm. Sci Total Environ 435: 472-478; doi:10.1016/j.scitotenv.2012.06.078 | No exposure assessment for the individual |
|  | Liu J, Liu CX, Wu T, Liu BP, Jia CX, Liu X (2019): Prolonged mobile phone use is associated with depressive symptoms in Chinese adolescents. J Affect Disord 259: 128-134; doi:10.1016/j.jad.2019.08.017 | Not EMF/health-related |
|  | Liu X, Luo Y, Liu ZZ, Yang Y, Liu J, Jia CX (2020): Prolonged Mobile Phone Use Is Associated with Poor Academic Performance in Adolescents. Cyberpsychol Behav Soc Netw 23 (5): 303-311; doi: 10.1089/cyber.2019.0591 | Not EMF/health-related |
|  | Liu Y, Chen W, Yang Y, Chen Y, Tang S (2021): Exposure to electronic screen before nocturnal sleep increases the risk of hypertensive disorders of pregnancy: A case-control study. J Obstet Gynaecol Res 47 (2): 698-704; doi: 10.1111/jog.14588 | Not EMF/health-related |
|  | Loughran SP, Verrender A, Dalecki A, Burdon CA, Tagami K, Park J, Taylor NAS, Croft RJ (2019): Radiofrequency Electromagnetic Field Exposure and the Resting EEG: Exploring the Thermal Mechanism Hypothesis. Int J Environ Res Public Health 16 (9): e1505; doi:10.3390/ijerph16091505 | Exposed or investigated age groups not relevant or unclear |
|  | Lu X, Oda M, Ohba T, Mitsubuchi H, Masuda S, Katoh T (2017): Association of excessive mobile phone use during pregnancy with birth weight: an adjunct study in Kumamoto of Japan Environment and Children's Study. Environ Health Prev Med 22 (1): 52; doi: 10.1186/s12199-017-0656-1 | Not EMF/health-related |
|  | Luo J, Deziel NC, Huang H, Chen Y, Ni X, Ma S, Udelsman R, Zhang Y (2019): Cell phone use and risk of thyroid cancer: a population-based case-control study in Connecticut. Ann Epidemiol 29: 39-45; doi:10.1016/j.annepidem.2018.10.004 | Exposed or investigated age groups not relevant or unclear |
|  | Lyngdoh M, Akoijam BS, Agui RS, Sonarjit Singh K (2019): Diet, Physical Activity, and Screen Time among School Students in Manipur. Indian J Community Med 44 (2): 134-137; doi: 10.4103/ijcm.IJCM_282_18 | Not EMF/health-related |
|  | Maes A, Collier M, Verschaeve L (2001): Cytogenetic effects of 900 MHz (GSM) microwaves on human lymphocytes. Bioelectromagnetics 22 (2): 91-96; doi:10.1002/1521-186X(200102)22:2< 91::AID-BEM1011> 3.0.CO;2-W | Exposed or investigated age groups not relevant or unclear |
|  | Mahmoudabadi FS, Ziaei S, Firoozabadi M, Kazemnejad A (2015): Use of mobile phone during pregnancy and the risk of spontaneous abortion. J Environ Health Sci Eng 13: 34; doi:10.1186/s40201-015-0193-z | Study on pregnancy outcomes/effects on fetus |
|  | Manago AM, Pacheco P (2019): Globalization and the Transition to Adulthood in a Maya Community in Mexico: Communication Technologies, Social Networks, and Views on Gender. New Dir Child Adolesc Dev 2019 (164): 11-25; doi: 10.1002/cad.20273 | Not EMF/health-related |
|  | Maskarinec G, Cooper J, Swygert L (1994): Investigation of increased incidence in childhood leukemia near radio towers in Hawaii: preliminary observations. J Environ Pathol Toxicol Oncol 13 (1): 33-37 | No exposure to mobile communications |
|  | McKenzie DR, Yin Y, Morrell S (1998): Childhood incidence of acute lymphoblastic leukaemia and exposure to broadcast radiation in Sydney - a second look. Aust N Z J Public Health 22 (3) Suppl: 360-367; doi:10.1111/j.1467-842x.1998.tb01392.x | No exposure to mobile communications |
|  | Meo SA, Almahmoud M, Alsultan Q, Alotaibi N, Alnajashi I, Hajjar WM (2019): Mobile Phone Base Station Tower Settings Adjacent to School Buildings: Impact on Students' Cognitive Health. Am J Mens Health 13 (1): 1557988318816914; doi:10.1177/1557988318816914 | No exposure assessment for the individual |
|  | Meo SA, Alsubaie Y, Almubarak Z, Almutawa H, AlQasem Y, Hasanato RM (2015): Association of Exposure to Radio-Frequency Electromagnetic Field Radiation (RF EMFR) Generated by Mobile Phone Base Stations with Glycated Hemoglobin (HbA1c) and Risk of Type 2 Diabetes Mellitus. Int J Environ Res Public Health 12 (11): 14519-14528; doi:10.3390/ijerph121114519 | No exposure assessment for the individual |
|  | Merrie YA, Tegegne MM, Munaw MB, Alemu HW (2019): Prevalence And Associated Factors Of Visual Impairment Among School-Age Children In Bahir Dar City, Northwest Ethiopia. Clin Optom (Auckl) 11: 135-143; doi: 10.2147/OPTO.S213047 | Not EMF/health-related |
|  | Merzenich H, Schmiedel S, Bennack S, Brüggemeyer H, Philipp J, Blettner M, Schüz J (2008): Childhood leukemia in relation to radio frequency electromagnetic fields in the vicinity of TV and radio broadcast transmitters. Am J Epidemiol 168 (10): 1169-1178; doi:10.1093/aje/kwn230 | No exposure to mobile communications |
|  | Michelozzi P, Capon A, Kirchmayer U, Forastiere F, Biggeri A, Barca A, Perucci CA (2002): Adult and childhood leukemia near a high-power radio station in Rome, Italy. Am J Epidemiol 155 (12): 1096-1103; doi:10.1093/aje/155.12.1096 | No exposure to mobile communications |
|  | Mireku MO, Barker MM, Mutz J, Dumontheil I, Thomas MSC, Röösli M, Elliott P, Toledano MB (2019): Night-time screen-based media device use and adolescents' sleep and health-related quality of life. Environ Int 124: 66-78; doi: 10.1016/j.envint.2018.11.069 | Not EMF/health-related |
|  | Misek J, Belyaev I, Jakusova V, Tonhajzerova I, Barabas J, Jakus J (2018): Heart rate variability affected by radiofrequency electromagnetic field in adolescent students. Bioelectromagnetics 39 (4): 277-288; doi:10.1002/bem.22115 | Exposed or investigated age groups not relevant or unclear |
|  | Moon JH, Cho SY, Lim SM, Roh JH, Koh MS, Kim YJ, Nam E (2019): Smart device usage in early childhood is differentially associated with fine motor and language development. Acta Paediatr 108 (5): 903-910; doi: 10.1111/apa.14623 | Not EMF/health-related |
|  | Moore SM, McIntosh RL, Iskra S, Wood AW (2015): Modeling the effect of adverse environmental conditions and clothing on temperature rise in a human body exposed to radio frequency electromagnetic fields. IEEE Trans Biomed Eng 62 (2): 627-637; doi:10.1109/TBME.2014.2362517 | Other experimental study (dosimetry), exposed or investigated age groups not relevant or unclear |
|  | Mortazavi SM, Shirazi KR, Mortazavi G (2013): The study of the effects of ionizing and non-ionizing radiations on birth weight of newborns to exposed mothers. J Nat Sci Biol Med 4 (1): 213-217; doi:10.4103/0976-9668.107293 | No exposure assessment for the individual, study on pregnancy outcomes/effects on fetus |
|  | Munezawa T, Kaneita Y, Osaki Y, Kanda H, Minowa M, Suzuki K, Higuchi S, Mori J, Yamamoto R, Ohida T (2011): The association between use of mobile phones after lights out and sleep disturbances among Japanese adolescents: a nationwide cross-sectional survey. Sleep 34 (8): 1013-1020; doi:10.5665/SLEEP.1152 | Not EMF/health-related |
|  | Murugesan G, Karthigeyan L, Selvagandhi PK, Gopichandran V (2018): Sleep patterns, hygiene and daytime sleepiness among adolescent school-goers in three districts of Tamil Nadu: A descriptive study. Natl Med J India 31 (4): 196-200; doi: 10.4103/0970-258X.258216 | Not EMF/health-related |
|  | Nagarjunakonda S, Amalakanti S, Uppala V, Gajula RK, Tata RS, Bolla HB, Rajanala L, Athina S, Daggumati R, Lavu H, Devanaboina AK (2017): Mobile phones and seizures: drug-resistant epilepsy is less common in mobile-phone-using patients. Postgrad Med J 93 (1095): 25-28; doi:10.1136/postgradmedj-2016-134140 | Exposed or investigated age groups not relevant or unclear |
|  | Nagata JM, Iyer P, Chu J, Baker FC, Pettee Gabriel K, Garber AK, Murray SB, Bibbins-Domingo K, Ganson KT (2021): Contemporary screen time modalities among children 9-10 years old and binge-eating disorder at one-year follow-up: A prospective cohort study. Int J Eat Disord 54 (5): 887-892; doi: 10.1002/eat.23489 | Not EMF/health-related |
|  | Nam KC, Kim SW, Kim SC, Kim DW (2006): Effects of RF exposure of teenagers and adults by CDMA cellular phones. Bioelectromagnetics 2006; 27 (7): 509-514 | Exposed or investigated age groups not relevant or unclear |
|  | Nathan N, Zeitzer J (2013): A survey study of the association between mobile phone use and daytime sleepiness in California high school students. BMC Public Health 13: 840; doi:10.1186/1471-2458-13-840 | Not EMF/health-related |
|  | Nishida T, Tamura H, Sakakibara H (2019): The association of smartphone use and depression in Japanese adolescents. Psychiatry Res 273: 523-527; doi: 10.1016/j.psychres.2019.01.074 | Not EMF/health-related |
|  | Nosetti L, Lonati I, Marelli S, Salsone M, Sforza M, Castelnuovo A, Mombelli S, Masso G, Ferini-Strambi L, Agosti M, Castronovo V (2021): Impact of pre-sleep habits on adolescent sleep: an Italian population-based study. Sleep Med 81: 300-306; doi: 10.1016/j.sleep.2021.02.054 | Not EMF/health-related |
|  | Odgers C (2018): Smartphones are bad for some teens, not all. Nature 554 (7693): 432-434; doi: 10.1038/d41586-018-02109-8 | Not EMF/health-related |
|  | Okada S, Doi S, Isumi A, Fujiwara T (2021): The association between mobile devices use and behavior problems among fourth grade children in Japan. Psychiatry Clin Neurosci 75 (9): 286-293; doi: 10.1111/pcn.13283 | Not EMF/health-related |
|  | Oshima N, Nishida A, Shimodera S, Tochigi M, Ando S, Yamasaki S, Okazaki Y, Sasaki T (2012): The suicidal feelings, self-injury, and mobile phone use after lights out in adolescents. J Pediatr Psychol 37 (9): 1023-1030; doi:10.1093/jpepsy/jss072 | Not EMF/health-related |
|  | Ouellet-Hellstrom R, Stewart WF (1993): Miscarriages among female physical therapists who report using radio- and microwave-frequency electromagnetic radiation. Am J Epidemiol 138 (10): 775-786; doi:10.1093/oxfordjournals.aje.a116781 | No exposure to mobile communications |
|  | Park SK, Ha M, Im HJ (2004): Ecological study on residences in the vicinity of AM radio broadcasting towers and cancer death: preliminary observations in Korea. Int Arch Occup Environ Health 77 (6): 387-394; doi:10.1007/s00420-004-0512-7 | No exposure to mobile communications |
|  | Pedrouzo SB, Peskins V, Garbocci AM, Sastre SG, Wasserman J (2020): Screen use among young children and parental concern. Arch Argent Pediatr 118 (6): 393-398; doi: 10.5546/aap.2020.eng.393 | Not EMF/health-related |
|  | Poulain T, Vogel M, Neef M, Abicht F, Hilbert A, Genuneit J, Körner A, Kiess W (2018): Reciprocal Associations between Electronic Media Use and Behavioral Difficulties in Preschoolers. Int J Environ Res Public Health 15 (4): 814; doi: 10.3390/ijerph15040814 | Not EMF/health-related |
|  | Poulain T, Ludwig J, Hiemisch A, Hilbert A, Kiess W (2019): Media Use of Mothers, Media Use of Children, and Parent-Child Interaction Are Related to Behavioral Difficulties and Strengths of Children. Int J Environ Res Public Health 16 (23): 4651; doi: 10.3390/ijerph16234651 | Not EMF/health-related |
|  | Preece AW, Georgiou AG, Dunn EJ, Farrow SC (2007): Health response of two communities to military antennae in Cyprus. Occup Environ Med 64 (6): 402-408; doi:10.1136/oem.2006.028894 | No exposure to mobile communications |
|  | Restrepo C, Santamaría A, Manrique R (2021): Sleep bruxism in children: relationship with screen-time and sugar consumption. Sleep Med X 3: 100035; doi: 10.1016/j.sleepx.2021.100035 | Not EMF/health-related |
|  | Rezk AY, Abdulqawi K, Mustafa RM, Abo El-Azm TM, Al-Inany H (2008): Fetal and neonatal responses following maternal exposure to mobile phones. Saudi Med J 29 (2): 218-223 | Exposure condition unclear, no exposure assessment for the individual, study on pregnancy outcomes/effects on fetus |
|  | Röösli M, Michel G, Kuehni CE, Spoerri A (2007): Cellular telephone use and time trends in brain tumour mortality in Switzerland from 1969 to 2002. Eur J Cancer Prev 16 (1): 77-82; doi:10.1097/01.cej.0000203618.61936.cd | No exposure assessment for the individual |
|  | Rothman KJ, Loughlin JE, Funch DP, Dreyer NA (1996): Overall mortality of cellular telephone customers. Epidemiology 7 (3): 303-305 | Exposed or investigated age groups not relevant or unclear |
|  | Rutter LA, Thompson HM, Howard J, Riley TN, De Jesús-Romero R, Lorenzo-Luaces L (2021): Social Media Use, Physical Activity, and Internalizing Symptoms in Adolescence: Cross-sectional Analysis. JMIR Ment Health 8 (9): e26134; doi: 10.2196/26134 | Not EMF/health-related |
|  | Sarı BA, Taner HA, Kaya ZT. Screen media exposure in pre-school children in Turkey: the relation with temperament and the role of parental attitudes (2021): Turk J Pediatr 63 (5): 818-831; doi: 10.24953/turkjped.2021.05.010 | Not EMF/health-related |
|  | Sato Y, Kiyohara K, Kojimahara N, Yamaguchi N (2016): Time trend in incidence of malignant neoplasms of the central nervous system in relation to mobile phone use among young people in Japan. Bioelectromagnetics 37 (5): 282-289; doi:10.1002/bem.21982 | Exposed or investigated age groups not relevant or unclear |
|  | Sato Y, Kojimahara N, Yamaguchi N (2017): Analysis of mobile phone use among young patients with brain tumors in Japan. Bioelectromagnetics 38 (5): 349-355; doi:10.1002/bem.22047 | No exposure assessment for the individual |
|  | Sato Y, Kojimahara N, Yamaguchi N (2019): Simulation of the incidence of malignant brain tumors in birth cohorts that started using mobile phones when they first became popular in Japan. Bioelectromagnetics 40 (3): 143-149; doi:10.1002/bem.22176 | No exposure assessment for the individual |
|  | Schmiedel S, Merzenich H, Bennack S, Brüggemeyer H, Philipp J, Blettner M, Schüz J (2009): Leukämien im Kindesalter und elektromagnetische Felder in der Umgebung von Rundfunkstationen - Ergebnisse einer Fall-Kontroll-Studie. Umweltmed Forsch Prax 14 (2): 79-90 | No exposure to mobile communications |
|  | Schreier N, Huss A, Röösli M (2006): The prevalence of symptoms attributed to electromagnetic field exposure: a cross-sectional representative survey in Switzerland. Soz Praventivmed 51 (4): 202-209; doi:10.1007/s00038-006-5061-2 | Exposed or investigated age groups not relevant or unclear |
|  | Schuster AK, Krause L, Kuchenbäcker C, Prütz F, Elflein HM, Pfeiffer N, Urschitz MS (2020): Prevalence and Time Trends in Myopia Among Children and Adolescents. Dtsch Arztebl Int 117 (50): 855-860; doi: 10.3238/arztebl.2020.0855 | Not EMF/health-related |
|  | Schüz J, Petters C, Egle UT, Jansen B, Kimbel R, Letzel S, Nix W, Schmidt LG, Vollrath L (2006): The "Mainzer EMF-Wachhund": results from a watchdog project on self-reported health complaints attributed to exposure to electromagnetic fields. Bioelectromagnetics 27 (4): 280-287; doi:10.1002/bem.20212 | Exposed or investigated age groups not relevant or unclear |
|  | Schweizer A, Berchtold A, Barrense-Dias Y, Akre C, Suris JC (2017): Adolescents with a smartphone sleep less than their peers. Eur J Pediatr 176 (1): 131-136; doi: 10.1007/s00431-016-2823-6 | Not EMF/health-related |
|  | Shen SY, Wang WH, Liang R, Pan GQ, Qian YM (2018): Clinicopathologic analysis of 2736 salivary gland cases over a 11-year period in Southwest China. Acta Otolaryngol 138 (8): 746-749; doi:10.1080/00016489.2018.1455108 | Exposed or investigated age groups not relevant or unclear |
|  | Smeds H, Wales J, Mathiesen T, Talbäck M, Feychting M (2018): Occurrence of primary brain tumors in cochlear implant patients in Sweden between 1989 and 2014. Clin Epidemiol 10: 1401-1405; doi:10.2147/CLEP.S164556 | No exposure to mobile communications |
|  | Smulevich VB, Solionova LG, Belyakova SV (1999): Parental occupation and other factors and cancer risk in children: II. Occupational factors. Int J Cancer 83 (6): 718-722; doi:10.1002/(SICI)1097-0215(19991210)83:6< 718::AID-IJC3> 3.0.CO;2-T | Exposed or investigated age groups not relevant or unclear |
|  | Söderqvist F, Carlberg M, Hardell L (2008): Use of wireless telephones and self-reported health symptoms: a population-based study among Swedish adolescents aged 15-19 years. Environ Health 7 (1): 18-1-18-10; doi:10.1186/1476-069X-7-18 | Exposed or investigated age groups not relevant or unclear |
|  | Sorahan T, Hamilton L, Gardiner K, Hodgson JT, Harrington JM (1999): Maternal occupational exposure to electromagnetic fields before, during, and after pregnancy in relation to risks of childhood cancers: findings from the Oxford Survey of Childhood Cancers, 1953-1981 deaths. Am J Ind Med 35 (4): 348-357; doi:10.1002/(sici)1097-0274(199904)35:4< 348::aid-ajim5> 3.0.co;2-x | No exposure to mobile communications |
|  | Stjernholm AD, Thysen SM, Borges IDS, Fisker AB (2021): Factors associated with birthweight and adverse pregnancy outcomes among children in rural Guinea-Bissau - a prospective observational study. BMC Public Health 21 (1): 1164; doi: 10.1186/s12889-021-11215-8 | Not EMF/health-related |
|  | Sudan M, Olsen J, Sigsgaard T, Kheifets L (2016): Trends in cell phone use among children in the Danish national birth cohort at ages 7 and 11 years. J Expo Sci Environ Epidemiol 26 (6): 606-612; doi:10.1038/jes.2016.17 | Not EMF/health-related |
|  | Tamura H, Nishida T, Tsuji A, Sakakibara H (2017): Association between Excessive Use of Mobile Phone and Insomnia and Depression among Japanese Adolescents. Int J Environ Res Public Health 14 (7): 701; doi: 10.3390/ijerph14070701 | Not EMF/health-related |
|  | Tan TC, Neo GH, Malhotra R, Allen JC, Lie D, Ostbye T (2014): Lifestyle Risk Factors Associated with Threatened Miscarriage: A Case-Control Study. JFIV Reprod Med Genet 2014, 2:2: 100123; doi:10.4172/jfiv.1000123 | Study on pregnancy outcomes/effects on fetus |
|  | Tashjian SM, Mullins JL, Galván A (2019): Bedtime Autonomy and Cellphone Use Influence Sleep Duration in Adolescents. J Adolesc Health 64 (1): 124-130; doi: 10.1016/j.jadohealth.2018.07.018 | Not EMF/health-related |
|  | Taskinen H, Kyyronen P, Hemminki K (1990): Effects of ultrasound, shortwaves, and physical exertion on pregnancy outcome in physiotherapists. J Epidemiol Community Health 44 (3): 196-201; doi:10.1136/jech.44.3.196 | No exposure to mobile communications |
|  | Tharner A, Mortensen AH, Holmsgaard EM, Væver MS (2022): Mothers' smartphone use and mother-infant interactive behavior in the postpartum period. Pediatr Res 91 (1): 8-11; doi: 10.1038/s41390-021-01451-4 | Not EMF/health-related |
|  | Tooth LR, Moss KM, Mishra GD (2021): Screen time and child behaviour and health-related quality of life: Effect of family context. Prev Med 153: 106795. doi: 10.1016/j.ypmed.2021.106795. | Not EMF/health-related |
|  | Trunk A, Stefanics G, Zentai N, Bacskay I, Felinger A, Thuroczy G, Hernadi I (2015): Effects of concurrent caffeine and mobile phone exposure on local target probability processing in the human brain. Sci Rep 5: 14434; doi:10.1038/srep14434 | Exposed or investigated age groups not relevant or unclear |
|  | Tynes T, Hannevik M, Andersen A, Vistnes AI, Haldorsen T (1996): Incidence of breast cancer in Norwegian female radio and telegraph operators. Cancer Causes Control 7 (2): 197-204; doi:10.1007/BF00051295 | No exposure to mobile communications, exposed or investigated age groups not relevant or unclear |
|  | Vaidyanathan S, Manohar H, Chandrasekaran V, Kandasamy P (2021): Screen Time Exposure in Preschool Children with ADHD: A Cross-Sectional Exploratory Study from South India. Indian J Psychol Med 43 (2): 125-129; doi: 10.1177/0253717620939782 | Not EMF/health-related |
|  | Van den Bulck J (2007): Adolescent use of mobile phones for calling and for sending text messages after lights out: results from a prospective cohort study with a one-year follow-up. Sleep 30 (9): 1220-1223. doi: 10.1093/sleep/30.9.1220 | Not EMF/health-related |
|  | Vecsei Z, Csatho A, Thuroczy G, Hernadi I (2013): Effect of a single 30 min UMTS mobile phone-like exposure on the thermal pain threshold of young healthy volunteers. Bioelectromagnetics 34 (7): 530-541; doi:10.1002/bem.21801 | Exposed or investigated age groups not relevant or unclear |
|  | Vernon L, Modecki KL, Barber BL (2018): Mobile Phones in the Bedroom: Trajectories of Sleep Habits and Subsequent Adolescent Psychosocial Development. Child Dev 89 (1): 66-77; doi: 10.1111/cdev.12836 | Not EMF/health-related |
|  | Voitl P (2021): Gesundheitliche Folgen exzessiver Smartphone-Verwendung bei Jugendlichen [Health consequences of excessive smartphone use in adolescents]. Monatsschr Kinderheilkd 15: 1-2; doi: 10.1007/s00112-021-01261-2 | Not EMF/health-related |
|  | Wada K, Yamakawa M, Konishi K, Goto Y, Mizuta F, Koda S, Uji T, Tamura T, Nakamura K, Tsuji M, Nagai H, Itakura N, Harada K, Takahara O, Yamanaka H, Nagata C (2019): Associations of Cell Phone Use and Screen Viewing with Overweight in Children. Child Obes 15 (7): 417-425; doi: 10.1089/chi.2018.0312 | Not EMF/health-related |
|  | Waisman I, Hidalgo E, Rossi ML (2018): Screen use among young children in a city of Argentina. Arch Argent Pediatr 116 (2): e186-e195; doi: 10.5546/aap.2018.eng.e186 | Not EMF/health-related |
|  | Waldmann P, Bohnenberger S, Greinert R, Hermann-Then B, Heselich A, Klug SJ, Koenig J, Kuhr K, Kuster N, Merker M, Murbach M, Pollet D, Schadenboeck W, Scheidemann-Wesp U, Schwab B, Volkmer B, Weyer V, Blettner M (2013): Influence of GSM Signals on Human Peripheral Lymphocytes: Study of Genotoxicity. Radiat Res 179 (2): 243-253; doi:10.1667/RR2914.1 | Other experimental study (in vitro) |
|  | Wang L, Liu X, Liu ZZ, Jia CX (2020): Digital media use and subsequent self-harm during a 1-year follow-up of Chinese adolescents. J Affect Disord 277: 279-286; doi: 10.1016/j.jad.2020.05.066 | Not EMF/health-related |
|  | Wilkins 3rd JR, Hundley VD (1990): Paternal occupational exposure to electromagnetic fields and neuroblastoma in offspring. Am J Epidemiol 131 (6): 995-1008; doi:10.1093/oxfordjournals.aje.a115620 | Exposed or investigated age groups not relevant or unclear |
|  | Wolf R, Wolf D (2004): Increased incidence of cancer near a cell-phone transmitter station. Int J Canc Prev 1 (2): 123-128 | Exposed or investigated age groups not relevant or unclear |
|  | Xie YJ, Cheung DS, Loke AY, Nogueira BL, Liu KM, Leung AY, Tsang AS, Leong CS, Molassiotis A (2020): Relationships Between the Usage of Televisions, Computers, and Mobile Phones and the Quality of Sleep in a Chinese Population: Community-Based Cross-Sectional Study. J Med Internet Res 22 (7): e18095; doi: 10.2196/18095 | Not EMF/health-related |
|  | Yang SY, Lin CY, Huang YC, Chang JH (2018): Gender differences in the association of smartphone use with the vitality and mental health of adolescent students. J Am Coll Health 66 (7): 693-701; doi: 10.1080/07448481.2018.1454930 | Not EMF/health-related |
|  | Yilmaz D, Yildiz M (2010): Analysis of the mobile phone effect on the heart rate variability by using the largest Lyapunov exponent. J Med Syst 34 (6): 1097-1103; doi:10.1007/s10916-009-9328-z | Exposed or investigated age groups not relevant or unclear |
|  | Yoon S, Choi JW, Lee E, An H, Kim HS, Choi HD, Kim N (2015): Mobile phone use and risk of glioma: a case-control study in Korea for 2002-2007. Environ Health Toxicol 30: e2015015; doi:10.5620/eht.e2015015 | Exposed or investigated age groups not relevant or unclear |
|  | Zarei S, Mortazavi SMJ, Mehdizadeh AR, Jalalipour M, Borzou S, Taeb S, Haghani M, Mortazavi SAR, Shojaei-fard MB, Nematollahi S, Alighanbari N, Jarideh S (2015): A Challenging Issue in the Etiology of Speech Problems: The Effect of Maternal Exposure to Electromagnetic Fields on Speech Problems in the Offspring. J Biomed Phys Eng 5 (3): 151-154 | Exposure condition unclear |
|  | Zarei S, Vahab M, Oryadi-Zanjani MM, Alighanbari N, Mortazavi SM (2019): Mother's Exposure to Electromagnetic Fields before and during Pregnancy is Associated with Risk of Speech Problems in Offspring. J Biomed Phys Eng 9 (1): 61-68; doi:10.31661/jbpe.v0i0.676 | Exposure condition unclear |
|  | Zeeni N, Doumit R, Abi Kharma J, Sanchez-Ruiz MJ (2018): Media, Technology Use, and Attitudes: Associations With Physical and Mental Well-Being in Youth With Implications for Evidence-Based Practice. Worldviews Evid Based Nurs 15 (4): 304-312; doi: 10.1111/wvn.12298 | Not EMF/health-related |
|  | Zhao D, Guo L, Zhang R, Zhu Q, Wang H, Liu R, Yan H, Dang S (2021): Risk of congenital heart disease due to exposure to common electrical appliances during early pregnancy: a case-control study. Environ Sci Pollut Res Int 2021; 28 (4): 4739-4748 | Study on pregnancy outcomes/effects on fetus |
|  | Zheng F, Gao P, He M, Li M, Wang C, Zeng Q, Zhou Z, Yu Z, Zhang L (2014): Association between mobile phone use and inattention in 7102 Chinese adolescents: a population-based cross-sectional study. BMC Public Health 14: 1022; doi:10.1186/1471-2458-14-1022 | Exposed or investigated age groups not relevant or unclear |
|  | Zhou LY, Zhang HX, Lan YL, Li Y, Liang Y, Yu L, Ma YM, Jia CW, Wang SY (2017): Epidemiological investigation of risk factors of the pregnant women with early spontaneous abortion in Beijing. Chin J Integr Med 23 (5): 345-349; doi:10.1007/s11655-015-2144-z | Study on pregnancy outcomes/effects on fetus, no exposure assessment for the individual |
