# Supplementary material for: Systematic review of the physiological and health-related effects of radiofrequency electromagnetic field exposure from wireless communication devices on children and adolescents in experimental and epidemiological human studies
Source: PLoS One. 2022 Jun 1;17(6):e0268641. doi: 10.1371/journal.pone.0268641 (PMC9159629; doi:10.1371/journal.pone.0268641)
Supplement: S3 Table — (DOCX) [file pone.0268641.s006.docx]

**S3 Table. Extracted Funds and Conflicts of Interests.**

| **Authors (Year)** | **Funds** | **Fund category (industry, NGO, public, mixed, no funds, not stated)** | **Conflict of Interest category (yes, no, not stated)** | **Effect category (effect/association found, limited effect/association, no effect/association)** |
| --- | --- | --- | --- | --- |
| Epidemiological studies | | | | |
| Abramson et al. (2009) | Australian Centre for Radiofrequency Bioeffects; National Health and Medical Research Council (NHMRC), Australia | public | not stated | limited association |
| Bhatt et al. (2017) | National Health and Medical Research Council (NHMRC), Australia | public | yes | limited association |
| Birks et al. (2017) | Augustinus Foundation, Denmark; Danish Epidemiology Science Centre, Denmark; Danish Medical Research Council (DMRC), Denmark; Department of Health, Basque Government, Spain; Egmont Foundation, Denmark; European Union (EU)/European Commission; Functional Genomics in Norway (FUGE) programme; Generalitat Catalunya, Spain; Generalitat Valenciana, Spain; ICT R&D program of MSIP/IITP (Ministry of Science, ICT and Future Planning/Institute for Information & Communications Technology Promotion), Korea; Instituto de Salud Carlos III., Ministerio de Sanidad y Consumo (Carlos III Health Institute, Ministry of Health and Consumption), Spain; Lundbeck Foundation, Denmark; March of Dimes, USA; Ministry of Environment (MOE), Korea; National Institute of Environmental Health Sciences (NIEHS), North Carolina, USA; National Institute of Environmental Research (NIER), Korea; National Institute of Neurological Disorders and Stroke (NINDS), USA; National Institutes of Health (NIH), Maryland, USA; Norwegian Ministry of Health and Care Services, Norway; Obra Social Cajastur, Spain; Provincial Government of Gipuzkoa, Spain; Research Council of Norway; University of Oviedo, Spain; ZonMw, The Netherlands | mixed | not stated | limited association |
| Brzozek et al. (2019) | National Health and Medical Research Council (NHMRC), Australia | public | yes | limited association |
| Byun et al. (2013) | Ministry of Environment (MOE), Korea; Ministry of Knowledge Economy (MKE), Korea | public | no | limited association |
| Cabré-Riera et al. (2020) | Stichting Volksbond Rotterdam, The Netherlands; Dutch Brain Foundation, The Netherlands; Brain & Behavior Research Foundation, The Netherlands; Netherlands Organization for Health Research and Development (ZonMW), The Netherlands; European Project GERONIMO (Generalised EMF Research using Novel Methods) funded under FP7-ENVIRONMEN; European Project ACTION (Aggression in Children: Unravelling gene-environment interplay to inform Treatment and InterventiON strategies); European Union’s Horizon 2020 research and innovation programme; Institute of Health Carlos III, Spain; French Agency for Food, Environmental and Occupational Health & Safety, France | public | no | limited association |
| Cabré-Riera et al. (2021) | European Project GERONIMO (Generalised EMF Research using Novel Methods) funded under FP7-ENVIRONMEN; Institute of Health Carlos III, Spain; Generalitat Valenciana, Spain; Generalitat de Catalunya, Spain; Ministry of Economy and Competitiveness, Spain; French Agency for Food, Environmental and Occupational Health & Safety, France; Department of Health of the Basque Government, Spain; Provincial Government of Gipuzkoa, Spain; Organisation for Health Research and Development, The Netherlands; Sarphati Amsterdam, The Netherlands; Ministry of Science and Innovation, Spain; State Research Agency, Spain | public | no | limited association |
| Cabré-Riera et al. (2022) | European Project GERONIMO (Generalised EMF Research using Novel Methods) funded under FP7-ENVIRONMEN; Institute of Health Carlos III, Spain; Generalitat de Catalunya, Spain; Fundacio ́ La Marato ́ de TV3; Ministry of Economy and Competitiveness, Spain; French Agency for Food, Environmental and Occupational Health & Safety, France; EU Commission; Ministry of Science and Innovation, Spain; State Research Agency, Spain; Department of Health of the Basque Government, Spain; Provincial Government of Gipuzkoa, Spain; Netherlands organization for Health Research and Development, The Netherlands | public | no | limited association |
| Castano-Vinyals et al. (2021) | European Community’s Seventh Framework Programme; Ministry of Science and Innovation, Spain; Health Research Fund (FIS) of the National Institute for Health Carlos III, Spain; Junta de Andalucía, Consejería de Salud, Spain; Generalitat de Catalunya, Spain; Australian National Health and Medical Research Council, Australia; Ministry of Science, Austria; university-industry partnership grant with Canadian Institutes of Health Research and Canadian Wireless Telecommunications Association; Canada; French National Agency for Sanitary Safety of Food, Environment and Labour, France; French National Cancer Institute, France; Pfizer Foundation; League against cancer, France; Federal Office for Radiation Protection, Germany; Hellenic Society for Social Pediatrics and Health Promotion, Greece; ELKE (Special Account for Research Grants of the National and Kapodistrian University of Athens), Greece; GGET (General Secretariat for Research and Technology), Greece; Board of Research in Nuclear Sciences, India; Ministry of Health, Italy; MSIT/IITP, Korea; Ministry of Internal Affairs and Communications, Japan; Health Research Council, New Zealand; Cure Kids, New Zealand; Organisation for Health Research and Development, The Netherlands; ODAS foundation, The Netherlands | mixed | no | no association |
| Chiu et al. (2015) | Bureau of Health Promotion, Department of Health, Taiwan | public | no | association found |
| Choi et al. (2017) | ICT R&D program of MSIP/IITP (Ministry of Science, ICT and Future Planning/Institute for Information & Communications Technology Promotion), Korea; Ministry of Environment (MOE), Korea; National Institute of Environmental Research (NIER), Korea | public | no | limited association |
| Çöl et al. (2021) | not stated | not stated | no | association found |
| Divan et al. (2008) | Danish Medical Research Council (DMRC), Denmark; Lundbeck Foundation, Denmark; Research Innovation Seed Grant; UCLA School of Public Health, USA | mixed | not stated | limited association |
| Divan et al. (2011) | Danish Medical Research Council (DMRC), Denmark; Lundbeck Foundation, Denmark; National Institute of Environmental Health Sciences/National Institute of Health (NIEHS/NIH), USA; UCLA School of Public Health, USA | public | not stated | no association |
| Divan et al. (2012) | Danish Medical Research Council (DMRC), Denmark; Lundbeck Foundation, Denmark; National Institute of Environmental Health Sciences/National Institute of Health (NIEHS/NIH), USA; UCLA School of Public Health, USA | mixed | no | limited association |
| Durusoy et al. (2017) | The Scientific and Technical Research Council of Turkey (TÜBITAK), Turkey | public | not stated | association found |
| Elliott et al. (2010) | Mobile Telecommunications and Health Research (MTHR), UK | public | yes | no association |
| Foerster et al. (2018) | Swiss National Science Foundation (SNF); European Project GERONIMO (Generalised EMF Research using Novel Methods) funded under FP7-ENVIRONMENT | public | no | association found |
| Guxens et al. (2013) | ZonMw, The Netherlands | public | no | no association |
| Guxens et al. (2016) | FIS (Fondo de Investigaciones Sanitarias, Health Research Fund) (Instituto de Salud Carlos III., Ministerio de Sanidad y Consumo) (Carlos III Health Institute, Ministry of Health and Consumption), Spain; ZonMw, The Netherlands | public | not stated | limited association |
| Guxens et al. (2019) | FIS (Fondo de Investigaciones Sanitarias, Health Research Fund) (Instituto de Salud Carlos III., Ministerio de Sanidad y Consumo) (Carlos III Health Institute, Ministry of Health and Consumption), Spain Programme "Electromagnetic Fields and Health Research", ZonMw, The Netherlands | public | no | limited association |
| Heinrich et al. (2010) | Deutsches Mobilfunk Forschungsprogramm (DMF; German Mobile Phone Research Programme) at Federal Office for Radiation Protection (BfS) | public | no | limited association |
| Heinrich et al. (2011) | Deutsches Mobilfunk Forschungsprogramm (DMF; German Mobile Phone Research Programme) at Federal Office for Radiation Protection (BfS) | public | not stated | no association |
| Huss et al. (2015) | FIS (Fondo de Investigaciones Sanitarias, Health Research Fund) (Instituto de Salud Carlos III., Ministerio de Sanidad y Consumo) (Carlos III Health Institute, Ministry of Health and Consumption), Spain; ZonMw, The Netherlands | public | no | no association |
| Lee et al. (2001) | not stated | not stated | not stated | association found |
| Milde-Busch et al. (2010) | Deutsches Mobilfunk Forschungsprogramm (DMF; German Mobile Phone Research Programme) at Federal Office for Radiation Protection (BfS) | public | no | no association |
| Mortazavi et al. (2011) | Center for Research in Radiation Science (CRRS), Iran | public | no | association found |
| Papadopoulou et al. (2017) | European Union (EU)/European Commission; National Institute of Environmental Health Sciences/National Institute of Health (NIEHS/NIH), USA; Norwegian Ministry of Health and Care Services, Norway | public | no | limited association |
| Redmayne et al. (2013) | not stated | no funds | yes | limited association |
| Redmayne et al. (2016) | National Health and Medical Research Council (NHMRC), Australia | public | yes | limited association |
| Roser et al. (2016) | Forschungsstiftung Mobilkommunikation (FSM; Research Foundation on Mobile Communication; at the Swiss Federal Institute of Technology Zurich (ETH)), Switzerland; Swiss National Science Foundation (SNF) | mixed | no | no association |
| Schoeni et al. (2015) | Swiss National Science Foundation (SNF) | public | not stated | association found |
| Schoeni et al. (2016) | Swiss National Science Foundation (SNF) | public | no | limited association |
| Schoeni et al. (2017) | Swiss National Science Foundation (SNF) | public | not stated | limited association |
| Sudan et al. (2012 | Danish Medical Research Council (DMRC), Denmark; Lundbeck Foundation, Denmark; National Institutes of Health (NIH), Maryland, USA; Netherlands Organisation for Scientific Research (NWO; Nederlandse organisatie voor Wetenschappelijk Onderzoek); The Netherlands | mixed | no | limited association |
| Sudan et al. (2013) | Danish Medical Research Council (DMRC), Denmark; Lundbeck Foundation, Denmark; National Institute of Environmental Health Sciences/National Institute of Health (NIEHS/NIH), USA; Netherlands Organisation for Scientific Research (NWO; Nederlandse organisatie voor Wetenschappelijk Onderzoek); The Netherlands | mixed | no | limited association |
| Sudan et al. (2016) | European Project GERONIMO (Generalised EMF Research using Novel Methods) funded under FP7-ENVIRONMENT | public | no | association found |
| Sudan et al. (2018) | GERoNiMO project: European Union. DNBC: Danish Epidemiology Science Centre; The Lundbeck Foundation (grant 195/04); Egmont Foundation; March of Dimes Birth Defect Foundation; Agustinus Foundation; and the Medical Research Council; Centers for Disease Control in Atlanta. INMA, European Union, Instituto de Salud Carlos III, Conselleria de Sanitat Generalitat Valenciana; Generalitat de Catalunya; Obra Social Cajastur; Universidad de Oviedo; Department of Health of the Basque Government; and the Provincial Government of Gipuzkoa. National Institute of Environmental Research, Korean Ministry of Environment and IT R& D program of MSIP/IITP | mixed | no | no association |
| Thomas et al. (2010a) | National Health and Medical Research Council (NHMRC), Australia | public | yes | limited association |
| Thomas et al. (2010b) | Deutsches Mobilfunk Forschungsprogramm (DMF; German Mobile Phone Research Programme) at Federal Office for Radiation Protection (BfS) | public | no | association found |
| Vrijheid et al. (2010) | not stated | not stated | not stated | limited association |
| Zheng et al. (2015) | National Basic Research Program (Program 973), China; National Natural Science Foundation (NSFC), China | public | no | association found |
| Experimental studies | | | | |
| Choi et al. (2014) | Ministry of Education, Science and Technology (MEST), Korea; National Research Foundation (NRF) of Korea | public | no | no effect |
| Croft et al. (2010) | GSM Association, UK/Ireland; National Health and Medical Research Council (NHMRC), Australia | mixed | not stated | no effect |
| Haarala et al. (2005) | Elisa Communications Corporation, Finland; Finnish 2G; Nokia; Sonera, Finland; Tekes (National Technology Agency), Finland; University of Turku, Finland | mixed | not stated | no effect |
| Krause et al. (2006) | Benefon, Finland; Elisa Communications Corporation, Finland; Finnish 2G; Nokia; Sonera, Finland; Tekes (National Technology Agency), Finland | mixed | not stated | effect found |
| Kwon et al. (2010) | Academy of Finland; Tekes (National Technology Agency), Finland; University of Helsinki, Finland | public | not stated | no effect |
| Leung et al. (2011) | GSM Association, UK/Ireland; National Health and Medical Research Council (NHMRC), Australia | mixed | not stated | effect found |
| Lindholm et al. (2011) | Tekes (National Technology Agency), Finland | public | not stated | no effect |
| Loughran et al. (2013) | Nationales Forschungsprogramm NFP 57 (National Research Programme NRP 57), Switzerland; Swiss National Science Foundation (SNF) | public | not stated | no effect |
| Movvahedi et al. (2014) | Shiraz University of Medical Sciences, Shiraz, Iran | public | no | effect found |
| Preece et al. (2005) | Charitable Trustees of the United Bristol Hospitals, Bristol, UK | NGO | not stated | no effect |
| Riddervold et al. (2008) | Danish Strategic Research Council, Denmark | public | not stated | no effect |
