# Supplementary material for: Systematic review of the physiological and health-related effects of radiofrequency electromagnetic field exposure from wireless communication devices on children and adolescents in experimental and epidemiological human studies
Source: PLoS One. 2022 Jun 1;17(6):e0268641. doi: 10.1371/journal.pone.0268641 (PMC9159629; doi:10.1371/journal.pone.0268641)
Supplement: S4 Table — (DOCX) [file pone.0268641.s007.docx]

### **S4 Table: Epidemiological studies on subjective symptoms in children and adolescents (n = 14).**

| Author (Year) (OHAT study-quality) | Study design Country Observation period | Study population Age  Number | Exposure  Assessment method  Exposure groups | Endpoints Assessment method | Results Conclusion according to authors  (Association categorization according to authors) |
| --- | --- | --- | --- | --- | --- |
| Cabré-Riera et al. (2022)  (1^st^ tier) | Cross-sectional study  The Netherlands and Spain  not stated | Preadolescents from the Generation R cohort and the INMA cohort  9–12 years  1,842 | Mobile phone and DECT (used for calls)  screen activities: mobile phones (used for internet browsing (other uses than calling), ect.)    tablet/laptop use  far-field sources (e.g., mobile phone base stations, radio/TV broadcast transmitters)  Whole-brain RF-EMF dose (whole day and evening): integrated RF-EMF exposure model based on objective and self-reported exposure variables as well as on various factors affecting near-field (e.g., WLAN, mobile phones, cordless phones) and far-field (e.g., mobile phone base stations, radio and TV broadcast transmitters, WiFi, mobile/cordless phones) RF EMF, SAR values, geospatial modeling, personal measurements of up to 72 hours in 56 Dutch adolescents of a previous study and in 148 Spanish preadolescents Calculation: mJ/kg per day | Sleep disturbances (problems with initiating and maintaining sleep, excessive somnolence, arousal problems, sleep quality and restfulness) and objective sleep measures (for 7 consecutive days): total sleep time, sleep efficiency, wake after sleep onset, sleep onset latency  Sleep Disturbance Scale for Children (filled in by mothers), wrist accelerometers | high evening whole-brain RF-EMF dose (>2.3 mJ/kg/evening) from phone calls: shorter total sleep time: -11.9 min (CI_95%_ -21.2; -2.5)  Conclusion: High evening whole-brain RF-EMF dose from phone calls had a shorter total sleep time. The associations might be due to the activities or reasons motivating the mobile phone calls rather the RF-EMF exposure itself or due to chance findings.  (Limited association) |
| Chiu et al. (2015)  (2^nd^ tier) | Cross-sectional study   Taiwan  2010 | Children   11–15 years   2,042 | Mobile phone:  CAPI (with parent): use (no (reference), yes), use in the last month,  use in 1 week,  average call duration/day | Subjective symptoms in the last month:  headache and migraine, excess daytime sleepiness, insomnia and skin itches  Worries about children’s health  CAPI (with parent) | Headache and migraine:  mobile phone use: OR 1.42 (CI_95%_ 1.12–1.81)  Skin itches:  mobile phone use: OR 1.84 (CI_95%_ 1.47–2.29)  Conclusion: Associations between the prevalence of headache and migraine as well as skin itches and the use of mobile phones.  (Association found) |
| Çöl et al. (2021)  (2^nd^ tier) | Cohort study  Turkey  not stated | Mother-child pairs  1 month – 5 years  400 | Mobile phone use during pregnancy (no (reference), ≥ 30 min/day)  WiFi use during pregnancy (no (reference), ≥ 1 hour/day)  Electronic media device use during pregnancy and presence in the sleeping environment of the children  *Only devices relevant for this review with quantifiable exposure characteristics were considered.*  Questionnaire (filled in by parents) | Sleep problems:  e.g., difficulties in falling asleep, bedtime resistance, nighttime waking, waking up crying, sleep duration  Questionnaire (filled in by parents) | Mobile phone use during pregnancy:  night waking: OR 7.48 (CI_95%_ 1.73–32.31),  waking up crying: OR 9.89 (CI_95%_ 1.87–52.35)  WiFi use during pregnancy: difficulties in falling asleep: OR 4.95 (CI_95%_ 2.00–12.22)  Conclusion: RF-EMF exposure caused by electronic media devices during pregnancy is associated with sleep problems in childhood.  (Association found) |
| Durusoy et al. (2017)  (2^nd^ tier) | Cross-sectional study  Turkey  2009–2011 | Adolescents  15.6 ± 1.3 years  2,150 | Mobile phone:  Questionnaire (filled in by adolescent): use (no (reference), yes),  number of calls/day (< 1 (reference), 1–4, 5–9 and ≥ 10), duration of calls (< 5 (reference), 5–9, 10–30 and > 30 min/day); control: number of text messages/day  further exposure related parameters determined   Mobile phone base station: Questionnaire (filled in by adolescent): base station nearby (no (reference), close to home, close to school, close to both home and school, unknown),  distance (none nearby or > 300 m (reference), ≤ 300 m)  Measurements (including radio waves, GSM, UMTS, DECT and WLAN) at school at spots with highest RF exposure in school building and school garden (1 year after the survey)  Data of mobile phone manufacturer: SAR value | Subjective symptoms in the last month:  fatigue, headache, nervousness/irritability, concentration problems, forgetfulness, sleep disturbances, depressive symptoms, sensitivity towards sounds, dizziness, blurred vision/visual disturbances, dryness of the throat, palpitation/change in cardiac rhythm, loss of appetite, difficulties in hearing, nausea, tremor and allergy  Symptoms during a call: warming of the ear, headache, discomfort during calls, numbness in the head or face, prickling in the ear and flushing  Questionnaire (filled in by adolescent) | Several significant results, e.g.:  Headache: mobile phone use: OR 1.90 (CI_95%_ 1.30–2.77)  Fatigue:  mobile phone use: OR 1.78 (CI_95%_ 1.21–2.63)   Sleep disturbances: mobile phone use: OR 1.53 (CI_95%_ 1.05–2.21)   Conclusion: Association between mobile phone use and specific symptoms.  (Association found) |
| Heinrich et al.  (2011)  (1^st^ tier) | Cross-sectional study   Germany  2006–2007 | Children and adolescents of the  MobilEe study   8–10 and 13–15 years   2,992 (1,484 children and 1,508 adolescents) | Mobile phone and DECT: CAPI (with child or adolescent): use less than nearly daily (reference), at least (nearly) daily  Mobile phone and base station (GSM 900, GSM 1800, UMTS 2100), DECT, WLAN 2400: Measurement (using a personal dosimeter) and calculation of the mean percentage of the ICNIRP reference levels during waking hours:  quartile 1: < 0.15%,  quartile 2: 0.15–0.17%,  quartile 3: 0.17–0.20% (children),  quartile 3: 0.17–0.21% (adolescents),  quartile 4: ≥ 0.20 resp. 0.21% | Chronic subjective symptoms in the last 6 months:  headache, irritation, nervousness, dizziness, fatigue, fear, and sleep problems  based on HBSC survey and assessed by Likert scale (filled in by participant) | Children  less sleep problems: quartile 3: OR 0.63 (CI_95%_ 0.41–0.96)  Adolescents  increased irritation:  at least daily mobile phone use: OR 1.48 (CI_95%_ 1.13–1.93), at least nearly daily cordless phone use: OR 1.30 (CI_95%_ 1.02–1.64)  Conclusion: No association between measured RF EMF exposure and chronic subjective symptoms in children and adolescents.  (No association) |
| Heinrich et al. (2010)  (1^st^ tier) | Cross-sectional study  Germany  2006–2008 (acc. to abstract; 2006–2007 acc. to study design section) | Children and adolescents of the  MobilEe study   8–10 and 13–15 years   2,992 (1,484 children and 1,508 adolescents) | Mobile phone: CAPI (with child or adolescent): call duration during morning and afternoon (≤ 5 min/day (reference), > 5 min/day)  Mobile phone and base station (GSM 900, GSM 1800, UMTS 2100), DECT, WLAN 2400:  Measurement (using a personal dosimeter) and calculation of the percentage of the ICNIRP reference levels during waking hours, morning and afternoon (in quartiles): no details provided for quartile categorization, but referenced | Acute subjective symptoms during 24-hours measurements:  headache, irritation, nervousness, dizziness, fatigue, and concentration problems  based on Zerssen complaint list and assessed by Likert scale (filled in by participant at noon and in the evening) | Children concentration problems in the evening:  quartile 4 in the afternoon: OR 1.55 (CI_95%_ 1.02–2.33)  Adolescents  headache at noon:  quartile 4 during morning hours: OR 1.50 (CI_95%_ 1.03–2.19), > 5 min mobile phone use during morning hours: OR 1.55 (CI_95%_ 1.05–2.29)  irritation in the evening:  quartile 4 during afternoon hours: OR 1.79 (CI_95%_ 1.23–2.61)  irritation at noon: quartile 4: OR 1.64 (CI 1.10–2.44), fatigue at noon: quartile 4: OR 1.76 (CI 1.22–2.56)  Conclusion: Few statistically significant results which were not consistent over the two time points (noon, evening) and might have rather occurred by chance.  (Limited association) |
| Huss et al. (2015)  (2^nd^ tier) | Cohort study  The Netherlands  2008–2009  follow up 2010–2011 | Children of the ABCD cohort  5–8 years  2,361 | Mobile phone, cordless phone, cordless phone base station, WiFi: Questionnaire (filled in by parent at child’s age 7 years):  At age 5 years: number of mobile phone calls/week (0 (reference), < 1, 1–2, ≥ 3, unknown), number of cordless phone calls/week (0 (reference), < 1, 1–2, ≥ 3, unknown), cordless phone base station and WiFi at home,  control: playing computer games and TV watching  At age 7 years: ownership of a mobile phone  Mobile phone base station at home at age 5 years: Calculation: exposure at home (low (< 50^th^ percentile), medium (50^th^–90^th^ percentile), high (> 90^th^ percentile))  Exposure at school:  Measurement and calculation | Sleep at the age of 7 years: sleep onset delay, sleep duration, night wakening, parasomnias, and daytime sleepiness  control parameters: bedtime resistance, sleep anxiety and sleep disordered breathing   CSHQ-questionnaire (filled in by parent) | Sleep duration:  mobile phone:  < 1 call/week: IRR 1.26 (CI_95%_ 1.08–1.46),  1–2 calls/week: IRR 1.42 (CI_95%_ 1.07–1.88),  ≥ 3 calls/week: IRR 1.43 (CI_95%_ 1.00–2.03)  Night wakening:  mobile phone: ≥ 3 calls/week: IRR 1.51 (CI_95%_ 1.02–2.23)  cordless phone: 1–2 calls/week: IRR 0.73 (CI_95%_ 0.53–0.99) (improvement)  Parasomnia: mobile phone: < 1 call/week: IRR 1.11 (CI_95%_ 1.01–1.23),  ≥ 3 calls/week: IRR 1.30 (CI_95%_ 1.04–1.63)  high exposure to mobile phone base stations: IRR 0.82 (CI_95%_ 0.69–0.97) (improvement)  Conclusion: Not RF EMF exposure is detrimental to sleep quality in 7-year old children, but potentially other factors that are related to mobile phone usage.  (No association) |
| Milde-Busch et al. (2010)  (2^nd^ tier) | Cross-sectional study  Germany  not stated | Adolescents of the MobilEe study  13–17 years  1,025 | Mobile phone:  CAPI (with adolescent): call duration (0 (reference), < 5, 6–15, 16–30, > 30 min/day)   Use of electronic media (e.g., computer/internet use, watching TV/videos):  CAPI (with adolescent) | Headache in the last 6 months: migraine, tension type headache and miscellaneous headache   ICHD-II criteria (filled in by adolescent) | Mobile phone: no statistically significant results (however, 77% of the participants reported little (< 5 min/day) or no mobile phone use)  Electronic media use:  few significant results   Conclusion: No consistent association between electronic media use and headache.  (No association) |
| Mortazavi et al. (2011)  (3^rd^ tier) | Cross-sectional study  Iran   not stated | Children and adolescents  6–16 years  452 | Mobile phone: Questionnaire (filled in by child/adolescent): duration of calls (no use (reference), < 10, > 11 min/day; (headache: additional categories 11–30, > 30 min/day)) | Subjective symptoms: headache, myalgia, palpitation, fatigue, tinnitus, concentration problems, attention problems, nervousness, vertigo and sleeping problems  Questionnaire (filled in by child/adolescent) | Some statistically significant, not adjusted results for all symptoms in association with mobile phone use as well as for headache, vertigo and sleeping problems in association with call duration.  Conclusion: Association between mobile phone use and some self-reported symptoms.  (Association found) |
| Redmayne et al. (2013)  (2^nd^ tier) | Cross-sectional study  New Zealand  2009 | Children  10–13 years  373 | Mobile phone, cordless phone, WLAN: Questionnaire: (filled in by child): mobile phone: number of calls (> 10 min/week: 0, 1–6, 7–35),  cordless phone: number of calls (> 10 min/week: 0, 1–2, 3–9, 10–120), call duration (0–4 (reference), 5–15, 16–240 min/day),  use and type of headset: no (reference), wired, wireless,  WLAN: WiFi at home (no, yes)  further exposure related parameters (cordless phone system and frequency range)  Questionnaire (filled in by parent): type of cordless phone, WiFi at home | Subjective symptoms in the last month: headache, tinnitus, feeling down/depressed,  depressive symptoms, wake in the night, trouble falling asleep, tired at school and painful texting thumb  HBSC checklist (filled in by child) | Several significant results, e.g.:  Headache:  > 6 mobile phone calls: OR 2.4 (CI_95%_ 1.2–4.8), > 15 min cordless phone use daily: OR 1.74 (CI_95%_ 1.1–2.9),  wireless headset: OR 2.2 (CI_95%_ 1.1–4.5) Tinnitus:  wired headset: OR 1.8 (CI_95%_ 1.0–3.3) Feeling down: wireless headset: OR 2.0 (CI_95%_ 1.1–3.8) Wake in the night:  wireless headset: OR 2.4 (CI_95%_ 1.2–4.8), WiFi at home: OR 0.7 (CI_95%_ 0.4–0.99) Tired at school: getting woken up at night by mobile phone: OR 3.49 (CI_95%_ 1.97–6.2)  Conclusion: There were more statistically significant associations (36%) than could be expected by chance (5%), whereby some associations may not be based on RF EMF exposure.  (Limited association) |
| Schoeni et al. (2016)  (1^st^ tier) | Cohort study  Switzerland  2012–2013, follow-up 2013–2014 | Adolescents of the  HERMES cohort  12–17 years (at baseline)  439,  425 in follow-up | Mobile phone base station at baseline: low (≤ 3.8 µW/m², ≤ 50^th^ percentile (reference)), medium (> 50^th^–≤ 75^th^ percentile),  high (> 11.0 µW/m², > 75^th^ percentile)   Radio and TV broadcast transmitters: low (≤ 1.0 µW/m², ≤ 50^th^ percentile (reference)), medium (> 50^th^–≤ 75^th^ percentile),  high (> 2.8 µW/m², > 75^th^ percentile)  Fixed site transmitters (all transmitters): low (≤ 5.8 µW/m², ≤ 50^th^ percentile (reference)), medium (> 50^th^–≤ 75^th^ percentile), high (> 13.4 µW/m², > 75^th^ percentile), total exposure (all groups)  Modelling using a geospatial propagation model | Subjective symptoms in the last 4 weeks: headache, tiredness, lack of energy, lack of concentration, exhaustibility, and physical well-being (physical activity, energy, fitness)  HIT-6 and Kidscreen-52 questionnaires, Likert scale (filled in by adolescent) | Change analysis: decreased exhaustibility: increase in total exposure to fixed site  transmitters OR 0.50 (CI_95%_ 0.27–0.93) (improvement)  decrease in lack of concentration: increase in base station exposure: OR 0.46 (CI_95%_ 0.24–0.88) (improvement)  Cohort (longitudinal) analysis:  tiredness:  base station: high exposure: OR 3.68 (CI_95%_ 1.76–7.66), high total exposure to fixed site transmitters: OR 2.94 (CI_95%_ 1.43–6.05)  lack of concentration: high exposure to broadcast transmitters: OR 2.78 (CI_95%_ 1.23–6.27)  Conclusion: No consistent association between self-reported symptoms and RF exposure from fixed site transmitters. The few observed associations have to be interpreted with caution and might represent chance findings.  (Limited association) |
| Schoeni et al. (2017)  (1^st^ tier) | Cohort study  Switzerland   2012–2013, follow-up 2013–2014 | Adolescents of the HERMES cohort  12–17 years (at baseline)  439,  425 in follow-up | Mobile phone and cordless phone:  Questionnaire (filled in by adolescent): duration of mobile and cordless phone calls, duration of data traffic on mobile phone; control: number of SMS sent per day, duration of gaming on computers and TV  Mobile phone: Data of mobile phone operators: duration of calls, network (GSM or UMTS), volume of data traffic; control: number of SMS sent per day  Cumulative brain and whole body dose (based on objective and self-reported exposure variables as well as on various factors affecting near-field (e.g., WLAN, mobile phones, cordless phones) and far-field (e.g., mobile phone base stations, radio and TV broadcast transmitters) RF EMF, SAR values from the literature and data of personal measurements) Calculation: mJ/kg per day | Subjective symptoms in the last 4 weeks: headache, tiredness, lack of energy, lack of concentration, exhaustibility and physical well-being/ill-being (physical activity, energy, fitness)  HIT-6 and Kidscreen-52 questionnaires, Likert scale (filled in by adolescent) | Cross-sectional analysis of baseline and follow-up data: few statistically significant results (e.g., associations with RF EMF and headache and exhaustibility; data only shown in figure)  Cohort analysis: several significant results, e.g.: tiredness: duration of data traffic on mobile phone OR 2.70 (CI_95%_ 1.52–4.80), duration of mobile phone calls OR 1.37; CI_95%_ 1.07–1.75)  lack of concentration: duration of data traffic on mobile phone OR 1.97 (CI_95%_ 1.14–3.40), duration of cordless phone calls OR 1.64 (CI_95%_ 1.27–2.12), duration of mobile phone calls OR 1.21 (CI_95%_ 1.03–1.44)  exhaustibility:  duration of data traffic on mobile phone OR 3.63 (CI_95%_ 2.09–6.31),  duration of cordless phone calls OR 1.35 (CI_95%_ 1.05–1.75),  cumulative whole body dose OR 1.34 (CI_95%_ 1.04–1.72)   lack of energy:  duration of data traffic on mobile phone OR 2.03 (CI_95%_ 1.23–3.35)   physical ill-being:  duration of data traffic on mobile phone OR 2.00 (CI_95%_ 1.20–3.36)  headache: cumulative whole body dose OR 1.37 (CI_95%_ 1.04–1.81)  Conclusion: Stronger associations between subjective symptoms and usage measures that are only marginally related to RF EMF exposure (e.g., tiredness and number of SMS) than for RF EMF exposure measures.  (Limited association) |
| Sudan et al. (2012)  (2^nd^ tier) | Cohort study  Denmark  1996–2002, follow-up 7 years later | mother-child pairs of the DNBC cohort  7 years   52,680 | Mobile phone:  Questionnaire (filled in by child at age of 7 years): use (postnatal: no (reference), yes)  Questionnaire (filled in by mother at child’s age 7 years): use during pregnancy (prenatal: no (reference), yes),  number of calls/day (0–1 (reference), 2–3, 4–6, ≥ 7)  Use of hands-free device during pregnancy (no exposure (reference), never use, rarely use, often use),  Percent of time mobile phone powered on (no exposure (reference), never or almost never, less than 50% of the time, 50-99% of the time, all the time) | Subjective symptoms:  migraine and headache-related symptoms (headache, stomach aches, sickness)  Questionnaire (filled in by mother at child’s age 7 years) | Migraine:  prenatal and postnatal exposure: OR 1.30 (CI_95%_ 1.01–1.68)  Headache-related symptoms:  prenatal and postnatal exposure: OR 1.32 (CI_95%_ 1.23–1.40),  only prenatal exposure: OR 1.16 (CI_95%_ 1.08–1.23),  only postnatal exposure: OR 1.28 (CI_95%_ 1.19–1.37)  similar significant results in analyses of number of calls/day, use of hands-free device and percent of time mobile phone powered on  Conclusion: Associations between prenatal and postnatal mobile phone use and migraine and headache-related symptoms, but results may not be causal.  (Limited association) |
| Zheng et al. (2015)  (2^nd^ tier) | Cross-sectional study   China   2011–2012 | Children  9–12 years  746 | Mobile phone:  Questionnaire (filled in by child): years of use (0 years (reference), 1 year, > 1 year),  daily duration of calls (1-year use and 0–10 min/day (reference), > 10 min/day;  > 1-year use and 0–10 min/day (reference), > 10 min/day) | Subjective symptoms in the past 6 months:  headache, dizziness, fatigue, sleeping problems, feeling low and heart beating fast  HBSC survey (filled in by child) | Fatigue: years of use > 1 year: OR 1.85 (CI_95%_ 1.07–3.22),  years of use > 1 year and daily use > 10 min: OR 2.98 (CI_95%_ 1.46–6.12)   Conclusion: There was a significant association between mobile phone use and fatigue in children.    (Association found) |

Note: If not stated otherwise, only statistically significant, adjusted results are provided.

Abbreviations: ABCD – Amsterdam Born Children and their Development Study, CAPI – Computer Assisted Personal Interview, CI_95%_ – 95%-Confidence Interval, CSHQ – Child Sleep Habits Questionnaire, DECT – Digital Enhanced Cordless Telecommunications, DNBC – Danish National Birth Cohort, EMF – Electromagnetic fields, GSM – Global System for Mobile Communications, HBSC – Health Behaviour in School-aged Children, HERMES – Health Effects Related to Mobile Phone Use in Adolescents, HIT-6 – Headache Impact Test, ICHD-II – International Classification of Headache Disorders, ICNIRP – International Commission on Non-Ionizing Radiation Protection, INMA – INfancia y Medio Ambiente (engl.: Environment and Childhood), IRR – Incidence Rate Ratio, mJ – Millijoule, MobilEe – Mobilfunk: Exposition und Befinden, OR – Odds Ratio, RF – Radiofrequency, SAR – Specific Absorption Rate, SMS – Short Message Service, UMTS – Universal Mobile Telecommunication System, WLAN – Wireless Local Area Network
