# Supplementary material for: Systematic review of the physiological and health-related effects of radiofrequency electromagnetic field exposure from wireless communication devices on children and adolescents in experimental and epidemiological human studies
Source: PLoS One. 2022 Jun 1;17(6):e0268641. doi: 10.1371/journal.pone.0268641 (PMC9159629; doi:10.1371/journal.pone.0268641)
Supplement: S5 Table — (DOCX) [file pone.0268641.s008.docx]

**S5 Table: Epidemiological studies on cognitive function in children and adolescents (n = 12).**

| Author (Year) (OHAT study quality) | Study design Country Observation period | Study population Age  Number | Exposure  Assessment method  Exposure groups | Endpoints Assessment method | Results Conclusion according to authors  (Association categorization according to authors) |
| --- | --- | --- | --- | --- | --- |
| Abramson et al. (2009)   (2^nd^ tier) | Cross-sectional study  Australia  2005–2006 | Children and adolescents of the MoRPhEUS study  11–14 years  317 | Mobile phone: Questionnaire (filled in by child or adolescent): number of calls per week (median 8, 25^th^ –75^th^ percentile: 4–15), number of text messages (SMS) per week (median 8, 25^th^–75^th^ percentile: 2.5–20) | Cognitive function:  signal detection, working memory, simple learning, associative learning and movement monitoring/estimation  CogHealth test battery and Stroop test | Working memory:  accuracy in one-back task: number of calls per week: regression coefficient -0.091 (CI_95%_ -0.170; -0.013), accuracy in two-back task: number of calls per week: regression coefficient -0.098 (CI_95%_ -0.169; -0.027)  Simple learning:  response time in one-card learning task: number of calls per week: regression coefficient -0.051 (CI_95%_ -0.083;  -0.020) (improvement)  Associative learning:  accuracy: number of calls per week: regression coefficient  -0.072 (CI_95%_ -0.108; -0.037),  response time: regression coefficient -0.028 (CI_95%_ -0.049;  -0.007) (improvement)  similar significant results for number of SMS messages per week  Conclusion: Mobile phone use was associated with faster and less accurate responding to higher level cognitive tasks, but these findings were unlikely due to RF exposure.  (Limited association) |
| Bhatt et al. (2017)  (2^nd^ tier) | Cohort study  Australia  2010–2012, follow-up 2012–2013   Update of Redmayne et al. (2016) | Children of the  ExPOSURE study  9–11 years  619, 412 in follow-up | Mobile phone and cordless phone:  Questionnaire (filled in by parent): number of calls per week (0 (reference), ≤ 2, > 2),  change in number of calls per week between baseline and follow-up (no change or decrease (reference), increase),  call duration per week, number of SMS per week  Calculation: change in number of mobile and cordless phone calls between baseline and follow-up (no change (reference), more calls per week)  Mobile phone: Questionnaire (filled in by child): owning and use, laterality of use and handedness | Cognitive function:  simple reaction time, choice reaction, working memory, visual attention, response inhibition, spatial and executive ability  CogHealth test battery and Stroop test | Mobile phone:  response time in response inhibition task: change in number of calls per week between baseline and follow-up: regression coefficient -0.030 (CI_95%_ -0.054; -0.006) (improvement),  accuracy in spatial and executive ability: change in number of calls per week between baseline and follow-up: regression coefficient 6.22 (CI_95%_ 2.13; 10.31),  response time in Stroop test: change in number of calls per week between baseline and follow-up: regression coefficient 0.056 (CI_95%_ 0.021; 0.090)  Cordless phone:  accuracy in detection task: change in number of calls per week between baseline and follow-up: regression coefficient  -0.069 (CI_95%_ -0.127; -0.012)  Conclusion: There was limited evidence that change in the use of mobile or cordless phones in children was associated with change in cognitive function. Due to the small numbers of mobile and cordless phone calls, the observed changes in cognitive tasks could be pure chance findings.  (Limited association) |
| Brzozek et al. (2019)  (2^nd^ tier) | Cohort study  Australia  2011-2013  Re-analysis of Bhatt et al. (2017) | Children of the  ExPOSURE study  9–11 years  619, 412 in follow-up | Mobile phone:  Questionnaires (filled in by parent): average number of calls per week after Monte Carlo simulation (0 (reference), ≤ 2 (low), > 2 (high))  Questionnaire (filled in by child): owning and use of a mobile phone, laterality of use and handedness | Cognitive function: simple reaction time, choice reaction, working memory, visual recognition and attention, response inhibition, spatial and executive ability  CogHealth test battery and Stroop test | Response time in response inhibition task:  low exposure: regression coefficient -0.023 (CI_95%_ -0.042; -0.004) (improvement), high exposure: regression coefficient -0.024 (CI_95%_ -0.046; -0.001) (improvement), Accuracy in spatial and executive ability:  low exposure: regression coefficient 4.53 (CI_95%_ 1.10; 7.96)  Response time in Stroop test:  low exposure: regression coefficient 0.034 (CI_95%_ 0.001; 0.068)  Conclusion: Limited evidence of an association between the use of mobile phones and cognitive function outcomes. Results consistent with Bhatt et al. (2017) but closer to null hypothesis.  (Limited association) |
| Cabré-Riera et al. (2021)  (1^st^ tier) | Cross-sectional study  The Netherlands and Spain  not stated | Preadolescents from the ABCD cohort and preadolescents/ adolescents from the INMA cohort  9–11 years and 17–18 years  2952 preadoslescents and 261 adolescents | Mobile phone and DECT (used for calls)  screen activities: mobile phones (used for internet browsing (other uses than calling), ect.)  tablet/laptop use  far-field sources (e.g., mobile phone base stations, radio/TV broadcast transmitters)  Whole-brain RF-EMF dose: integrated RF-EMF exposure model based on objective and self-reported exposure variables as well as on various factors affecting near-field (e.g., WLAN, mobile phones, cordless phones) and far-field (e.g., mobile phone base stations, radio and TV broadcast transmitters, WiFi, mobile/cordless phones) RF EMF, SAR values estimated in a previous study, geospatial modeling, personal measurements of up to 72 hours in 56 preadolescents of the Dutch cohort and in 191 preadolescents and 53 adolescents from the Spanish cohort Calculation: mJ/kg per day | Cognitive function: non-verbal intelligence, information processing, attention, cognitive flexibility, working memory, semantic verbal fluency  validated neurocognitive tests (Raven (-like) test, Wechsler Intelligence Scale for Children IV, Attention Network Task, Trail Making test part A and part B, N-back test, Semantic Verbal Fluency test) | Non-verbal intelligence: whole-brain RF-EMF dose: beta coefficient − 0.10 (CI_95%_ -0.19; − 0.02) per 100 mJ/kg/day increase  specific RF-EMF dose from mobile and cordless phone calls: beta coefficient − 0.10 (CI_95%_ -0.19; − 0.02) per 100 mJ/kg/day increase  Conclusion: The results suggest that higher brain exposure to RF-EMF is related to lower non-verbal intelligence but not to other cognitive function outcomes. The authors cannot discard chance finding, or reverse causality.  (Limited association) |
| Foerster et al. (2018)  (1^st^ tier) | Cohort study  Switzerland  2012–2014, follow-up 2014–2016   Update of Schoeni et al. (2015) | Adolescents of the HERMES cohort  12–17 years (at baseline)  895, 843 in follow-up | Mobile phone and cordless phone: Questionnaire (filled in by adolescent): duration of mobile and cordless phone calls (min/day), duration of data traffic on mobile phone (min/day); preferential side of head for phone use; cumulative usage per day; control: number of SMS sent per day, duration of gaming on computers and TV  Mobile phone: Data of mobile phone operators (for n = 234 participants): number and duration of calls, network (GSM or UMTS), volume of data traffic; control: number of SMS sent per day  Cumulative brain RF EMF dose based on objective (operator data) and self-reported exposure variables as well as on various factors affecting near-field (e.g., WLAN, mobile phones, cordless phones) and far-field (e.g., mobile phone base stations, radio and TV broadcast transmitters) RF EMF, SAR simulations (for different exposure scenarios), geospatial modeling and data of personal measurements (for n = 148 participants for 3 days). Additionally, self-reported call duration was calibrated by objective operator-recorded call duration:  Calculation: mJ/kg per day | Memory performance Verbal and figural memory  Intelligenz-Struktur test | Figural memory: several significant results, e.g.:  cumulative RF EMF brain dose (whole sample):  coefficient (change in score per IQR): -0.22 (CI_95%_ -0.47; 0.03; IQR 953 mJ/kg/day)  cumulative RF EMF brain dose (based on operator data): coefficient (change in score per IQR):  -0.26 (CI_95%_ -0.42; -0.10, IQR 341 mJ/kg/day)   cumulative RE-EMF brain dose for users with right-side preference: coefficient (change in score per IQR): -0.39 (CI_95%_ -0.67; -0.10, IQR 953 mJ/kg/day)  Conclusion: The results suggest a potential adverse effect of RF EMF brain dose on cognitive functions that involve brain regions mostly exposed during mobile phone use.  (Association found) |
| Guxens et al. (2016)  (2^nd^ tier) | Cross-sectional study  The Netherlands  2008–2010 | Children of the ABCD cohort  5–6 years  2,354 | Mobile phone, cordless phone, cordless phone base station, WiFi at age 5 years: number of mobile phone calls per week (0 (reference), < 1, 1–2, ≥ 3),  number of cordless phone calls per week (0 (reference), < 1, 1–2, ≥ 3),  cordless phone base station and WiFi at home,  control: gaming (computer or video games) Questionnaire (filled in by mother at child’s age 7 years)  Residential mobile phone base station at age 5 years: residential exposure low (< 50^th^ percentile) (reference), medium (50^th^–90^th^ percentile), high (> 90^th^ percentile) Calculation using a 3D geospatial radio wave propagation model  RF EMF exposure at school:  Measurements for 478 children   Mobile phone base station at school: Calculation for 349 children | Cognitive function:  speed of information processing, inhibitory control, cognitive flexibility and visuomotor coordination   Amsterdam Neuro-psychological Tasks program | Presence of cordless phone base station and WiFi at home:  improved speed of information processing,  improved inhibitory control, improved cognitive flexibility, improved visuomotor coordination   Residential exposure to mobile phone base stations ≥ 50^th^ percentile:  improved inhibitory control, improved cognitive flexibility, reduced visuomotor coordination   Number of cordless phone calls per week > 0:  reduced inhibitory control,  reduced cognitive flexibility  Number of mobile phone calls per week > 0: improved visuomotor coordination  *Remark: coefficients and CI_95%_ are displayed in figures, numbers are not provided*   Conclusion: There were inconsistent associations between different sources of RF EMF exposure and cognitive functions.  (Limited association) |
| Lee et al. (2001)  (2^nd^ tier) | Cross-sectional study   China  not stated | Adolescents  15–16 years  72 | Mobile phone (GSM): no use (reference), use (175–27,240 min in total), calculated (based on the average duration of phone use per day multiplied by the number of days that the GSM phone system has been installed) Questionnaire (filled in by adolescent) | Attention   Symbol Digit Modalities test, Stroop test, Trail Making test | Attention:  mobile phone users: improved attention in Trail Making test (p< 0.05 in ANOVA test)  Conclusion: RF EMF exposure emitted by mobile phones may have a mild facilitating effect on attention.  (Association found) |
| Redmayne et al. (2016)   (2^nd^ tier) | Cross-sectional study  Australia  2011 | Children of the  ExPOSURE study  8–11 years   619 | Mobile phone: Questionnaire (filled in by parent): number of calls per week (0 (reference 1),  ≤ 2.5 (reference 2), > 2.5), number of SMS per week  Questionnaire (filled in by child): owning and use  Cordless phone:  Questionnaire (filled in by parent): number of calls per week (0 (reference 1), ≤ 2 (reference 2), > 2) | Cognitive function:  simple reaction time and psychomotor speed, choice reaction and visual attention, working memory, response inhibition, visual recognition and episodic memory  CogHealth test battery and Stroop test | Mobile phone: response time in response inhibition: > 2.5 calls per week (reference 1): regression coefficient 0.029 (CI_95%_ 0.003; 0.054),  (reference 2): regression coefficient 0.03 (CI_95%_ n.a., p = 0.01)  Cordless phone: response time in Stroop test: > 2 calls per week (reference 2): regression coefficient 0.035 (CI_95%_ n.a., p = 0.007), accuracy in visual recognition and episodic memory: > 2 calls per week (reference 2): regression coefficient -0.03 (CI_95%_ n.a., p = 0.02), accuracy in choice reaction and visual attention: > 2 calls per week (reference 2): regression coefficient -0.06 (CI_95%_ n.a., p = 0.05)  Conclusion: There was little evidence that cognitive function was associated with mobile and cordless phone use.  (Limited association) |
| Roser et al. (2016)  (same study as in S6 Table, but different endpoint!)  (1^st^ tier) | Cohort study   Switzerland  2012–2013, follow-up 2013–2014 | Adolescents of the  HERMES cohort  12–17 years (at baseline)  439,  425 in follow-up | Mobile phone and cordless phone: Questionnaire (filled in by adolescent): duration of mobile and cordless phone calls (min/day), duration of data traffic on mobile phone (min/day); control: number of SMS sent per day, duration of gaming on computer, laptops, tablets and TV  Mobile phone: Data of mobile phone operators (for n = 234 participants): number and duration of calls, network (GSM or UMTS), volume of data traffic; control: number of SMS sent per day  Mobile phone base stations, television broadcasting, total RF EMF exposure, total RF EMF exposure without mobile phone:  Measurements (using personal dosimeters in 91 participants for 3 days)  Cumulative brain and whole body RF EMF dose based on objective and self-reported exposure variables as well as on various factors affecting near-field (e.g., WLAN, mobile phones, cordless phones) and far-field (e.g., mobile phone base stations, radio and TV broadcast transmitters) RF EMF, SAR values from the literature, geospatial modeling, personal measurements Calculation: mJ/kg per day | Concentration capacity: homogeneity, power and accuracy of concentration   Cognitive test battery FACT-II   Behavior *see S6 Table* | Cross-sectional analysis:  several significant results, e.g.: homogeneity of concentration:  total exposure without mobile phone: coefficient (change in score per IQR) -3.50 (CI_95%_ -6.79; -0.20)  power of concentration:  total exposure without mobile phone: coefficient (change in score per IQR) -6.00 (CI_95%_ -10.30; -1.71)  Longitudinal analysis:  no statistically significant results  Conclusion: The lack of consistent exposure-response patterns in the longitudinal analyses suggests that concentration capacity is not affected by the use of wireless communication devices or RF EMF exposures. Information bias and reverse causality are likely explanations for the observed cross-sectional findings.  (No association) |
| Schoeni et al. (2015)   (1^st^ tier) | Cohort study  Switzerland   2012–2013, follow-up 2013–2014 | Adolescents of the HERMES cohort  12–17 years (at baseline)  439, 425 in follow-up | Mobile phone and cordless phone: Questionnaire (filled in by adolescent): duration of mobile and cordless phone calls (min/day), duration of data traffic on mobile phone (min/day); control: number of SMS sent per day, duration of gaming on computers and TV  Mobile phone: Data of mobile phone operators (for n = 234 participants): duration of calls, network (GSM or UMTS), volume of data traffic; control: number of SMS sent per day  Cumulative brain and whole body RF EMF dose based on objective (operator data) and self-reported exposure variables as well as on various factors affecting near-field (e.g., WLAN, mobile phones, cordless phones) and far-field (e.g., mobile phone base stations, radio and TV broadcast transmitters) RF EMF, SAR values from the literature and data of personal measurements (for n = 95 participants for 3 days):  Calculation: mJ/kg per day | Memory performance Verbal and figural memory   Intelligenz-Struktur test | Figural memory:  several significant results, e.g.: cumulative RF EMF brain dose (based on operator data): regression coefficient (change in score per IQR)  -0.26 (CI_95%_ -0.42; -0.10),  cumulative RF EMF whole body dose (based on operator data): regression coefficient (change in score per IQR)  -0.40 (CI_95%_ -0.79; -0.01)  Conclusion: A change in memory performance over one year was negatively associated with cumulative duration of wireless phone use and more strongly with RF EMF dose. This may indicate that RF EMF impair the memory performance in adolescents.  (Association found) |
| Sudan et al. (2018)  (2^nd^ tier) | Pooled analysis of 3 birth cohorts   Denmark, Spain, and Korea  1996–2011 | Mother-child-pairs of the GERoNiMO Project including the DNBC, INMA and MOCEH cohorts  4–6 years  3,089 | Mobile phone: Questionnaire (filled in by mother: during pregnancy in INMA and MOCEH cohorts, at child’s age 7 years in DNBC cohort): use during pregnancy:  number of calls/day (0, low (0–1) reference, medium (2–3), high (≥ 4) | General, verbal and non-verbal cognition at age 4–6 years   WPPSI-R in Denmark and Korea, MSCA in Spain | No statistically significant results  Conclusion: A general pattern of lower cognition scores in association to high frequency use compared to low frequency use across all cognitive dimensions were observed, but the findings were imprecise.  (No association) |
| Thomas et al. (2010a)  (2^nd^ tier) | Cohort study  Australia  2005–2006, follow-up 2006–2007   Update of Abramson et al. (2009) | Adolescents of the  MoRPhEUS study  12–15 years   317,  232 in follow-up | Mobile phone: Questionnaire (filled in by adolescent): number of calls per week (median 10, 25^th^–75^th^ percentile: 5–21), number of text messages (SMS) per week (median 10, 25^th^—75^th^ percentile: 4–20) | Cognitive function:  signal detection, working memory, learning, associative learning, and movement monitoring  CogHealth test battery and Stroop test | Signal detection: response time in simple reaction task: difference in number of SMS per week between baseline and follow-up: 0.024 (CI_95%_ 0.007; 0.042)  Working memory:  response time two-back task: number of calls per week at baseline: regression coefficient 0.042 (CI_95%_ 0.004; 0.079) (similar result for number of SMS per week)  response time in two-back task: difference in number of calls per week between baseline and follow-up: regression coefficient -0.079 (CI_95%_ -1.156; -0.003) (improvement)  Learning:  response time in one-card learning task: number of calls per week at baseline: regression coefficient 0.045 (CI_95%_ 0.009; 0.081)  Conclusion: This study showed changes in response times over a 1-year period in some of the cognitive tasks but no change in accuracy. This may relate to statistical regression to the mean and not be the effect of mobile phone exposure.  (Limited association) |

Note: If not stated otherwise, only statistically significant, adjusted results are provided.

Abbreviations: ABCD – Amsterdam Born Children and their Development Study, CI_95%_ – 95%-Confidence Interval, DNBC – Danish National Birth Cohort, DECT – Digital Enhanced Cordless Telecommunications, EMF – Electromagnetic fields, ExPOSURE – Examination of Psychological Outcomes in Students using Radiofrequency Devices, FACT-II – Frankfurt Adaptive Concentration Test, GERoNiMO – Generalized EMF research using novel methods, GSM – Global System for Mobile Communications, HERMES – Health Effects Related to Mobile Phone Use in Adolescents, INMA – Infancia y Medio Ambiente, IQR – Interquartile range, mJ – Millijoule, MSCA – McCarthy Scales of Children's Abilities, MOCEH – Korean Mothers and Children's Environmental Health Study, MoRPhEUS – Mobile Radiofrequency Phone Exposed Users’ Study, n.a. – not available, OR – Odds Ratio, RF – Radiofrequency, SAR – Specific Absorption Rate, SMS – Short Message Service, UMTS – Universal Mobile Telecommunication System, WLAN – Wireless Local Area Network, WPPSI-R –Welchsler Preschool and Primary Scale of Intelligence – Revised
