# Supplementary material for: Systematic review of the physiological and health-related effects of radiofrequency electromagnetic field exposure from wireless communication devices on children and adolescents in experimental and epidemiological human studies
Source: PLoS One. 2022 Jun 1;17(6):e0268641. doi: 10.1371/journal.pone.0268641 (PMC9159629; doi:10.1371/journal.pone.0268641)
Supplement: S6 Table — (DOCX) [file pone.0268641.s009.docx]

**S6 Table. Epidemiological studies on behavior in children and adolescents (n = 9).**

| Author (Year) (OHAT study quality) | Study design Country Observation period | Study population Age  Number | Exposure  Assessment method  Exposure groups | Endpoints Assessment method | Results Conclusion according to authors  (Association categorization according to authors) |
| --- | --- | --- | --- | --- | --- |
| Birks et al. (2017)  (2^nd^ tier) | multinational analysis of 5 birth cohorts from Denmark, Korea, the Netherlands, Norway, and Spain   1996–2011 | Mother-child pairs of the ABCD, DNBC, INMA, MoBa and MOCEH cohorts (GERoNiMO Project)   5–7 years  83,884 | Mobile phone and cordless phone (only in DNBC cohort): Questionnaire (filled in by mother: prospectively during pregnancy in 3 cohorts, retrospectively at child’s age 7 years in 2 cohorts):  use during pregnancy:  number of calls/day (0, 0–1 (reference), 2–3, ≥ 4) | Behavioral problems:  overall behavioral problems, hyperactivity/inattention problems and emotional problems and peer relationship/social problems   SDQ in 3 cohorts, CBCL in 2 cohorts (filled in by parents) | Hyperactivity/inattention problems:  2–3 mobile phone calls/day: OR 1.11 (CI_95%_ 1.01–1.22),  ≥ 4 mobile phone calls/day: OR 1.28 (CI_95%_ 1.12–1.48)   Conclusion: Maternal mobile phone use during pregnancy may be associated with an increased risk for behavioral problems, particularly hyperactivity/inattention problems, in the offspring. The interpretation of these results is unclear as uncontrolled confounding may have influenced both maternal mobile phone use and child behavioral problems.  (Limited association) |
| Byun et al. (2013)  (2^nd^ tier) | Cohort study  Korea  2008, follow-up 2 years later | Children of the CHEER study  approx. 7–12 years  2,422 | Mobile phone: e.g., age when first owned a mobile phone, number of calls/day,  average call duration (< 30 s (reference), 30–60 s, ≥ 1 min),  number of text messages per day,  use of Internet on a mobile phone, cumulative time spent for voice calls using own mobile phone based on number of calls per day, average call duration and years of own mobile phone use (0 hours (reference), < 30, 30–< 70, ≥ 70 hours)  Questionnaire (filled in by parent or guardian)  Blood lead level: Measurement (low 2.35 μg/dL, high: ≥ 2.35 μg/dL) | ADHD:  K-ARS rating scale (filled in by parents or guardians) | ADHD in the group of high blood lead level:  average call duration 30-60 s: OR 5.7 (CI_95%_ 1.31–24.51)  average call duration ≥ 1 min: OR 7.2 (CI_95%_ 1.37–37.91)  Conclusion: The ADHD symptom risk was associated with mobile phone use for voice calls but the association was limited to children with relatively high blood lead levels.  (Limited association) |
| Divan et al. (2008)  (2^nd^ tier) | Cohort study   Denmark  1997–1999, follow-up 2006 | Mother-child pairs of the DNBC cohort   7 years   13,159 | Mobile phone and cordless phone:  Questionnaire (filled in by mother at child’s age 7 years): use during pregnancy (prenatal: no (reference), yes),  number of calls/day (0–1 (reference), 2–3, ≥ 4), percentage of time turned on   child: use at age 7 years (postnatal: no (reference), yes) | Behavioral problems:  overall behavioral problems, emotional symptoms, conduct problems, hyperactivity and peer relationship problems  SDQ (filled in by mother) | Several significant results, e.g.:  Overall behavioral problems:  prenatal and postnatal exposure: OR 1.80 (CI_95%_ 1.45–2.23),  only prenatal exposure: OR 1.54 (CI_95%_ 1.32–1.81),  only postnatal exposure: OR 1.18 (CI_95%_ 1.01–1.38)   Conclusion: Prenatal and, to a lesser degree, postnatal exposure to mobile phones was associated with behavioral problems in children at the age of 7 years. These associations may be non-causal and may be due to unmeasured confounding.  (Limited association) |
| Divan et al. (2012)  (2^nd^ tier) | Cohort study  Denmark  1997–2002 follow-up 7 years later   Update of Divan et al. (2008) | Mother-child pairs of the DNBC cohort   7 years   41,541 | Mobile phone:  Questionnaire (filled in by mother at child’s age 7 years): use during pregnancy (prenatal: no (reference), yes) number of calls/day (0–1 (reference),  2–3, ≥ 4),  percentage of time turned on  child: use at age 7 years (postnatal: no (reference), yes) | Behavioral problems:  overall behavioral problems (no further details provided)   SDQ (filled in by mother) | Several significant results, e.g.:  Overall behavioral problems:  prenatal and postnatal exposure: OR 1.5 (CI_95%_ 1.4–1.7), only prenatal exposure: OR 1.4 (CI_95%_ 1.2–1.5), only postnatal exposure: OR 1.2 (CI_95%_ 1.0–1.3)  Conclusion: The findings of the previous publication (Divan et al. , 2008) were replicated in this expanded group of participants and confirmed.  (Limited association) |
| Guxens et al. (2013)  (2^nd^ tier) | Cohort study  The Netherlands  2003–2004, follow-up at age 5 years | Mother-child pairs of the ABCD cohort   5 years  2,618 | Mobile phone and cordless phone: Questionnaire (filled in by mother at child’s age 7 years): use during pregnancy: number of calls/day (0 (reference), < 1, 1–4, ≥ 5) | Behavior problems at age 5 years:  total difficulties (emotional symptoms, conduct problems, hyperactivity/inattention problems and peer relationship problems)  Strengths (prosocial behavior)  SDQ (filled in by mother and teacher) | No statistically significant results  Conclusion: The results do not suggest that maternal mobile phone or cordless phone use during pregnancy increases the odds of behavior problems in their children.  (No association) |
| Guxens et al. (2019)  (2^nd^ tier) | Cross-sectional study   The Netherlands  2008–2009 | Children of the ABCD cohort   5 years  3,102 | Mobile phone, cordless phone, cordless phone base station, WiFi: number of calls/week (0 (reference), < 1, 1–2, ≥ 3),  cordless phone base station and WiFi at home (yes/no) Questionnaire (filled in by mother at child’s age 7 years)  Computer/video games use and television watching (control) Questionnaire (filled in by mother at child’s age 5 years)  Residential RF EMF exposure to mobile phone base stations (at age of 5 years) low (< 50^th^ percentile), medium (50^th^–90^th^ percentile), high (> 90^th^ percentile) Calculation using 3D geospatial radio wave propagation model | Behavior at age 5 years: overall problems (emotional symptoms, conduct problems, hyperactivity/inattention and peer relationship problems)   Strengths (prosocial behavior)  SDQ (filled in by mother and teacher) | Several significant results, e.g.:  Behavior reported by mother: emotional symptoms: high RF EMF level from mobile phone base stations: OR 1.82 (CI_95%_ 1.07–3.09),  peer relationship problems:  < 1 mobile phone call/week: OR 0.61 (CI_95%_ 0.42–0.91), cordless phone base station: OR 0.61 (CI_95%_ 0.39–0.96) (improvement)  Behavior reported by teacher: conduct problems:  medium RF EMF level from mobile phone base stations: OR 1.37 (CI_95%_ 1.02–1.83), prosocial behavior: cordless phone base station: OR 0.68 (CI_95%_ 0.48–0.97) (improvement), overall behavioral problems: < 1 mobile phone call/week: OR 0.67 (CI_95%_ 0.47–0.95) (improvement)  Conclusion: Mobile phone and cordless phone calls were not associated with behavioral problems in children at age of 5 years. Environmental RF EMF exposure from mobile phone base stations and television watching, which both contribute very little to RF EMF exposure, were associated with specific emotional and behavioral problems. The authors cannot discard residual confounding or reverse causality.  (Limited association) |
| Roser et al. (2016)  (same study as in S5 Table, but different endpoint!)   (1^st^ tier) | Cohort study  Switzerland  2012–2013, follow-up 2013–2014 | Adolescents of the HERMES study  12–17 years (at baseline)  439,  425 in follow-up | Mobile phone and cordless phone: Questionnaire (filled in by adolescent): duration of mobile and cordless phone calls (min/day), duration of data traffic on mobile phone (min/day); control: number of SMS sent per day, duration of gaming on computer, laptops, tablets and TV  Mobile phone: Data of mobile phone operators (for n = 234 participants): number and duration of calls, network (GSM or UMTS), volume of data traffic; control: number of SMS sent per day  Mobile phone base stations, television broadcasting, total RF EMF exposure, total RF EMF exposure without mobile phone:  Measurements (using personal dosimeters in 91 participants for 3 days)  Cumulative brain and whole body RF EMF dose based on objective and self-reported exposure variables as well as on various factors affecting near-field (e.g., WLAN, mobile phones, cordless phones) and far-field (e.g., mobile phone base stations, radio and TV broadcast transmitters) RF EMF, SAR values from the literature, geospatial modeling, personal measurements Calculation: mJ/kg per day | Behavior:  total difficulties (emotional symptoms, conduct problems, hyperactivity/inattention and peer relationship problems)  Strengths (prosocial behavior)  SDQ (filled in by adolescent and parent)  Cognitive function: *see S5 Table* | Cross-sectional analysis:  several significant results, e.g.: total difficulties (self-reported by adolescent):  duration data traffic mobile phone: coefficient 0.98 (CI_95%_ 0.41; 1.55),  duration of mobile phone calls: coefficient 0.28 (CI_95%_ 0.13; 0.43), duration of cordless phone calls: coefficient 0.36 (CI_95%_ 0.12; 0.59) RF EMF brain dose: coefficient 0.33 (CI_95%_ 0.15; 0.50),  RF EMF whole body dose: coefficient 0.41 (CI_95%_ 0.25; 0.57) RF EMF whole body dose (operator sample): coefficient 1.19 (CI_95%_ 0.70; 1.68)  Longitudinal analysis:  total difficulties (self-reported by adolescent):  duration of mobile phone calls: coefficient -0.34 (CI_95%_ -0.59;  -0.08) (improvement) RF EMF brain dose: coefficient -0.61 (CI_95%_ -0.93; -0.28) (improvement), RF EMF whole body dose: coefficient -0.39 (CI_95%_ -0.63; -0.14) (improvement)  Conclusion: The lack of consistent exposure-response patterns in the longitudinal analyses suggests that behavioral problems are not affected by the use of wireless communication devices or RF EMF exposure. Information bias and reverse causality are likely explanations for the observed cross-sectional findings.  (No association) |
| Sudan et al. (2016)  (2^nd^ tier) | Cohort study  Denmark  1996–2002, follow-up at age 7 and 11 years   Update of Divan et al. (2008) and Divan et al. (2012) | Mother-child pairs of the DNBC cohort  7–11 years   47,721 | Mobile phone: Questionnaire (filled in by mother at child’s age 7 years): use during pregnancy (prenatal: no (reference), yes)  number of calls/day (0–1 (reference),  2–3, ≥ 4), percentage of time turned on,  use of hands-free equipment   child: use at age 7 years (postnatal: no (reference), yes), duration (no use (reference), < 1, > 1 hour/week) | Emotional and behavioral difficulties   SDQ (filled in by mother) | Several significant results, e.g.:  Emotional and behavioral difficulties at age 11 years (in children with no difficulties at age 7 years): prenatal and postnatal exposure: OR 1.58 (CI_95%_ 1.34–1.86),  only prenatal exposure: OR 1.41 (CI_95%_ 1.20–1.66),  only postnatal exposure: OR 1.36 (CI_95%_ 1.14–1.63)   Conclusion: The findings are consistent with pattern seen in earlier studies and suggest that both prenatal and postnatal exposures may be associated with increased risks of emotional and behavioral difficulties in children.  (Association found) |
| Thomas et al. (2010b)  (1^st^ tier) | Cross-sectional study   Germany  2006–2008 | Children and adolescents of the MobilEe study   8–12 and 13–17 years   2,992  (1,484 children and 1,508 adolescents) | Mobile phone and mobile phone base station (GSM 900, GSM 1800, UMTS 2100, DECT, WLAN 2400: Measurement using a personal dosimeter  Calculation of the percentage of the ICNIRP reference levels based on the field strength during waking hours:  quartile 1: ≤ 0.15%,  quartile 2: > 0.15–0.17%,  quartile 3: > 0.17–0.20% (children),  quartile 3: > 0.17–0.21% (adolescents),  quartile 4: > 0.20 resp. 0.21% | Behavioral problems:  emotional symptoms, conduct problems, hyperactivity/inattention and peer relationship problems   SDQ (filled in by child and parent or adolescent) | Children  conduct problems:  quartile 4: OR 2.9 (CI_95%_ 1.4–5.9)   Adolescents total behavioral problems: quartile 4: OR 2.2 (CI_95%_ 1.1–4.5),  conduct problems:  quartile 4: OR 3.7 (CI_95%_ 1.6–8.4)   Conclusion: An association between measured exposure to RF EMF and behavioral problems in children and adolescents in the highest exposure groups was observed.  (Association found) |

Note: If not stated otherwise, only statistically significant, adjusted results are provided.

Abbreviations: ABCD – Amsterdam Born Children and their Development Study, ADHD – Attention Deficit Hyperactivity Disorder, CBCL – Child Behavior Checklist, CHEER – Children’s Health and Environment Research, CI_95%_ – 95%-confidence Interval, DECT – Digital Enhanced Cordless Telecommunications, DNBC – Danish National Birth Cohort, EMF – Electromagnetic Field, GERoNiMO – Generalized EMF research using novel methods, GSM – Global System for Mobile Communications, HERMES – Health Effects Related to Mobile Phone Use in Adolescents, ICNIRP – International Commission on Non-Ionizing Radiation Protection, INMA – Infancia y Medio Ambiente, K-ARS – Korean version of ADHD rating scale, mJ – Millijoule, MobilEe – Mobilfunk: Exposition und Befinden, MoBa – The Norwegian Mother and Child Cohort Study, MOCEH – Korean Mothers and Children's Environmental Health Study, OR – Odds Ratio, RF – Radiofrequency, SAR – Specific Absorption Rate, SDQ – Strengths and Difficulties Questionnaire, SMS – Short Message Service, UMTS – Universal Mobile Telecommunication System, WLAN – Wireless Local Area Network
