# Supplementary material for: Systematic review of the physiological and health-related effects of radiofrequency electromagnetic field exposure from wireless communication devices on children and adolescents in experimental and epidemiological human studies
Source: PLoS One. 2022 Jun 1;17(6):e0268641. doi: 10.1371/journal.pone.0268641 (PMC9159629; doi:10.1371/journal.pone.0268641)
Supplement: S7 Table — (DOCX) [file pone.0268641.s010.docx]

**S7 Table. Epidemiological studies on infant development (n = 4).**

| Author (Year) (OHAT study-quality) | Study design Country Observation period | Study population Age  Number | Exposure  Assessment method  Exposure groups | Endpoints Assessment method | Results Conclusion according to authors  (Association categorization according to authors) |
| --- | --- | --- | --- | --- | --- |
| Choi et al. (2017)  (2^nd^ tier) | Cohort study  Korea  2006–2010, follow-up at age 6, 12, 24 and 36 months | Mother-child pairs of the MOCEH cohort   6–36 months  1,198 | Mobile phone:  Questionnaire (filled in by mother during pregnancy): use during pregnancy: number of calls/day (≤ 2, 3–5, ≥ 6), calling time (< 3, 3–< 10, 10–< 30, ≥ 30 min/day)  RF EMF (e.g., FM, TV, TETRA, PCS, IMT):  Measurement with personal exposimeters (in 210 mothers)  Blood lead level of the mother during pregnancy:  Measurement: low: < 1.69 μg/dL, high: ≥ 1.69 μg/dL | Neurodevelopment:  mental and psychomotor development at age 6, 12, 24 and 36 months   Korean version of Bayley Scale | Several significant results, e.g.:  Mental Development:  high blood lead level in relation to increasing number of calls/day (*p-trend 0.007*) (decrease)  high blood lead level in relation to increasing calling time (*p-trend 0.05*) (decrease)  Psychomotor development:  high blood lead level in relation to increasing calling time (*p-trend 0.008*) (decrease)  Conclusion: No association between prenatal mobile phone or RF EMF exposure and child neurodevelopment during the first 3 years. However, a potential combined effect of prenatal exposure to lead and mobile phone exposure was suggested.  (Limited association) |
| Divan et al. (2011)  (2^nd^ tier) | Cohort study  Denmark  1996–2002, follow-up at age 6 and 18 months | Mother-child pairs of the DNBC cohort  6 and 18 months   41,541 | Mobile phone: Questionnaire (filled in by mother at child’s age 7 years): use during pregnancy: number of calls/day (0–1 (reference), 2–3, ≥ 4),  Percentage of time turned on | Motor, cognitive and language development  Interview (with mother at child’s age 6 und 18 months) | No statistically significant results   Conclusion: No evidence of an association between prenatal mobile phone exposure and motor, cognitive and language delays among infants at age 6 and 18 months.  (No association) |
| Papado-poulou et al.  (2017)  (2^nd^ tier) | Cohort study  Norway  1999–2008 | Mother-child pairs of the MoBa cohort   3–5 years  45,389 (3 years) and 17,310 (5 years) | Mobile phone: Questionnaire (filled in by mother in 17^th^ and 30^th^ week of pregnancy): use during pregnancy (no (reference), yes; no/seldom (reference), few times a week, daily, > 1 hour/day) | Language development (at 3 years), communication and motor skills (at 3 and 5 years)   Dale and Bishop Grammar rating, Ages and Stages questionnaire and Child Development Inventory (assessed by mother) | Language development, sentence complexity:  mobile phone use (yes): OR 0.83 (KI_95%_ 0.77–0.89) (improvement)  Motor skills: mobile phone use (yes): OR 0.82 (KI_95%_ 0.76–0.87) (improvement)  similar significant results in subgroups  Conclusion: No evidence of adverse neurodevelopmental effects of prenatal mobile phone exposure was observed. An improved language and motor development at age of 3 years were found.  (Limited association) |
| Vrijheid et al. (2010)  (2^nd^ tier) | Cohort study  Spain   2004–2006 | Mother-child pairs of the INMA cohort   14 months   530 | Mobile phone: Questionnaire (filled in by mother in week 32 of pregnancy): use during pregnancy:  number of calls/day (0 (reference), 1, 2–4, ≥ 5) | Neurodevelopment: mental and psychomotor development   Bayley Scales | Psychomotor development:  ≥ 5 calls/day: regression coefficient -5.6 (CI_95%_ -10.7; -0.5)  Conclusion: The study gives little evidence for an adverse effect of maternal mobile phone use during pregnancy on the early development of offspring.  (Limited association) |

Note: If not stated otherwise, only statistically significant, adjusted results are provided.

Abbreviations: DNBC – Danish National Birth Cohort, IMT – International Mobile Telecommunications, INMA – Infancia y Medio Ambiente, CI_95%_ – 95%-Confidence Interval, MoBa – The Norwegian Mother and Child Cohort Study, MOCEH – Korean Mothers and Children's Environmental Health Study, OR – Odds Ratio, PCS – Personal Communications System, TETRA – Terrestrial Trunked Radio
