# Supplementary material for: Systematic review of the physiological and health-related effects of radiofrequency electromagnetic field exposure from wireless communication devices on children and adolescents in experimental and epidemiological human studies
Source: PLoS One. 2022 Jun 1;17(6):e0268641. doi: 10.1371/journal.pone.0268641 (PMC9159629; doi:10.1371/journal.pone.0268641)
Supplement: S8 Table — (DOCX) [file pone.0268641.s011.docx]

**S8 Table. Epidemiological studies on other endpoints of children and adolescents (n = 4).**

| Author (Year) (OHAT study quality | Study design Country Observation period | Study population  Age  Number | Exposure  Assessment method  Exposure groups | Endpoints Assessment method | Results Conclusion according to authors  (Association categorization according to authors) |
| --- | --- | --- | --- | --- | --- |
| Cabré-Riera et al. (2020)  (1^st^ tier) | Cross-sectional study  The Netherlands  2013-2015 | Preadolescents from the Generation R cohort  9–12 years  2,592 | Mobile phone and DECT (used for calls)  screen activities: mobile phones (used for internet browsing (other uses than calling), ect.)  tablet/laptop use  far-field sources (e.g. mobile phone base stations, radio/TV broadcast transmitters)  Whole-brain and lobe-specific RF-EMF dose: integrated RF-EMF exposure model based on objective and self-reported exposure variables as well as on various factors affecting near-field (e.g., WLAN, mobile phones, cordless phones) and far-field (e.g., mobile phone base stations, radio and TV broadcast transmitters, WiFi, mobile/cordless phones) RF EMF, SAR values, geospatial modeling, personal measurements of up to 72 hours in 56 preadolescents of a previous study Calculation: mJ/kg per day | Brain volume: total brain, cortical gray matter, cortical white matter, cerebellar gray matter, and cerebellar white matter, frontal, parietal, temporal, and occipital lobes, hippocampus, amygdala, thalamus, putamen, caudate, nucleus accumbens, and pallidum  Magnetic resonance imaging (MRI) | Whole-brain RF-EMF dose from screen activities associated with a smaller caudate volume: beta coefficient −5.02 mm^3^ (CI_95%_ −7.78; −2.25)  Conclusion: Effects may reflect social or individual factors related to the specific uses of devices rather than effects of RF-EMF exposure. Residual confounding, chance finding or reverse causality cannot be discarded.  (Limited association) |
| Castano-Vinyals et al. (2021)  (2^nd^ tier) | Case-control study  Australia, Austria, Canada, France, Germany, Greece, India, Israel, Italy, Japan, Korea, the Netherlands, New Zealand, Spain  2010-2015 | Children from the MOBI-Kids study  10-14 years  378 cases and 783 controls  Additionally, young people aged 15-19 years (217 cases and 616 controls) and aged 20-24 years (167 cases and 468 controls) were investigated | Mobile communication devices use (mobile phone, cordless (DECT) phones) and WiFi, exposure to domestic sources of EMF such as induction cookers and chargers  regular phone use 1 year before diagnosis, time since start of use, cumulative number of calls, cumulative call time, cumulative RF specific energy and ELF induced current density  Interviews mainly with parents in age group 10-14 years (performed by trained interviewers); data partly validated by operator data and an app installed on the mobile phones | Brain tumors (benign or malignant)  Histopathology or diagnostic imaging | No statistically significant results.  Conclusion: No evidence of an association between wireless phone use and brain tumors in children was found.  (No association) |
| Elliott et al. (2010)  (1^st^ tier) | Case-control study   United Kingdom  1999–2001 | Children  4 years   1,397 cases and 5,588 controls | Mobile phone base station (GSM 900 and GSM 1800): Measurements and modelling based on operator data: distance between birth address and nearest mobile phone base station,  total power output from summation across all base stations within 700 m, modelled power density at each birth address for base stations within 1400 m | Early childhood cancers: brain and CNS tumors, leukemia and non-Hodgkin’s lymphoma  Cancer registry | No statistically significant results  Conclusion: There was no association between risk for early childhood cancers and estimates of the mother’s exposure to mobile phone base stations during pregnancy.  (No association) |
| Sudan et al. (2013)  (2^nd^ tier) | Cross-sectional study  Denmark  2003–2009 | Children of the DNBC cohort  7 years  52,680 | Mobile phone: Questionnaire (filled in by mother at child’s age 7 years): use at age 7 years (no (reference), yes,  duration (no use (reference), < 1, > 1 hour/week) | Hearing loss at age 7 years   Questionnaire (filled in by mother) | Hearing loss:  marginal structure model: use (yes): OR 1.23 (CI_95%_ 1.01–1.49)  Conclusion: A weak association between mobile phone use  and hearing loss at age 7 was found, but this could have been affected by various biases and is not sufficient to conclude that mobile phone exposure has an effect on hearing.  (Limited association) |

Note: If not stated otherwise, only statistically significant, adjusted results are provided.

Abbreviations: CI_95%_ – 95%-Confidence Interval, CNS – Central nervous system, DECT – Digital Enhanced Cordless Telecommunications, DNBC – Danish National Birth Cohort, GSM – Global System for Mobile Communications, OR – Odds Ratio
