# Supplementary material for: Systematic review of the physiological and health-related effects of radiofrequency electromagnetic field exposure from wireless communication devices on children and adolescents in experimental and epidemiological human studies
Source: PLoS One. 2022 Jun 1;17(6):e0268641. doi: 10.1371/journal.pone.0268641 (PMC9159629; doi:10.1371/journal.pone.0268641)
Supplement: S9 Table — (DOCX) [file pone.0268641.s012.docx]

**S9 Table. Experimental studies on brain activity and cognition in children and adolescents (n = 9).**

| Author (Year)  (OHAT study quality) | Study type  Study population  Age | Exposure source  Assessment method  Exposure conditions | Endpoints Assessment method | Results  Conclusion according to authors  (Effect categorization according to authors) |
| --- | --- | --- | --- | --- |
| Croft et al. (2010)  (2^nd^ tier) | Double-blind cross-over study  Adolescents (male and female, n = 41)  13–15 years  Additionally, young adults (n = 42), 19–40 years, and elderly (n = 20), 55–70 years, were investigated | Mobile phone, GSM 894 MHz (2G) and WCDMA 1,900 MHz (3G):  Measurements and calculation: 3 conditions: sham exposure, 2G (peak SAR_10g_: 0.7 W/kg) and 3G (peak SAR_10g_: 1.7 W/kg)  exposure of the head  Exposure duration: 3 x 55 min, separated by at least 4 days | Brain activity (EEG):  resting alpha activity | No statistically significant effects  Conclusion: No evidence was found for an effect of mobile phone exposure on resting alpha activity in adolescents.  (No effect) |
| Haarala et al. (2005)  (1^st^ tier) | Double-blind cross-over study  Children (boys and girls, n = 32)  10–14 years | Mobile phone, GSM 902 MHz:  Phantom measurement:  2 conditions: sham exposure and exposure (mean power: 0.25 W, SAR_10g_: 0.990 W/kg, SAR_1g_: 1.44 W/kg, peak SAR: 2.07 W/kg)  exposure of the head  Exposure duration: 2 x approximately 50 min, separated by 24 h (± 1 h) | Cognitive functions:  reaction time and accuracy, vigilance and short-term memory  Reaction time software and N-back test | No statistically significant effects  Conclusion: Mobile phone has no effect on children’s cognitive functions.  (No effect) |
| Krause et al. (2006)  (2^nd^ tier) | Double-blind cross-over study  Children (boys and girls, n = 15)  10–14 years | Mobile phone, GSM 902 MHz:  Measurement in an adult phantom:  2 conditions: sham exposure and exposure (SAR_1g_: 1.40 W/kg, peak SAR: 1.98 W/kg)  exposure of the head  Exposure duration: 2 x 30 min, separated by a short break | Brain activity (EEG):  Event-related desynchronization/synchronization  during performing an auditory memory task | Effects on brain oscillatory responses in the EEG frequencies approximately 4–8 Hz and approximately 15 Hz during cognitive processing.  Conclusion: EMF emitted by mobile phones has effects on brain oscillatory responses during cognitive processing in children.  (Effect found) |
| Kwon et al. (2010)  (2^nd^ tier) | Single-blind cross-over study  Children (boys and girls, n = 17)  11–12 years | Mobile phone, GSM 902 MHz:  Measurement in an adult phantom: 2 conditions: sham exposure and exposure (SAR_1g_: 1.14 W/kg, SAR_10g_: 0.82 W/kg, peak SAR: 1.21 W/kg, mean power: 0.25 W);  exposure of left and right ear  Exposure duration: 2 x 6 min exposure, 1 x 6 min sham exposure per ear | Brain activity (EEG):  Auditory event-related potentials P1, N2, mismatch negativity and P3a | No statistically significant effects  Conclusion: Short exposure to mobile phone EMF has no statistically significant effects on the neural change-detection profile.  (No effect) |
| Leung et al. (2011)  (1^st^ tier) | Double-blind cross-over study  Adolescents (male and female, n = 41)  13–15 years  Additionally, young adults (n = 42), 19–40 years, and elderly (n = 20), 55–70 years, were investigated | Mobile phone, GSM 894,6 MHz (2G) and WCDMA 1,900 MHz (3G):  Measurements and calculation:  3 conditions: sham exposure, 2G (peak SAR_10g_: 0.7 W/kg) and 3G (peak SAR_10g_: 1.7 W/kg);  exposure of the head  Exposure duration: 3 x 55 min, separated by at least 4 days | Brain activity (EEG)  Cognitive functions:  cognitive processing, reaction time and accuracy  Auditory event-related potentials  during Oddball task and N-back task | Auditory 3-stimulus oddball task: augmented N1 amplitude in 2G condition,  reduced accuracy in N-back task in 3G condition,  delayed event-related desynchronization/ synchronization responses of the alpha power in both 2G and 3G conditions.  Conclusion: This study provides support for an  effect of acute 2G and 3G exposure on human cognitive function.  (Effect found) |
| Loughran et al. (2013)  (2^nd^ tier) | Double-blind, randomized cross-over study  Adolescents (male and female, n = 22)  11–13 years | Planar antenna, GSM 900 MHz, mobile phone-like modulation:  Calculation: 3 conditions: sham exposure, high SAR (psSAR: 1.4 W/kg), low SAR (psSAR: 0.35 W/kg);  exposure of the head  Exposure duration: 3 x 30 min, at weekly intervals at the same time of the day | Brain activity (EEG)  Cognitive functions:  reaction time and accuracy  Simple reaction time task, 2-choice reaction time task and N-back task | No statistically significant effects  Conclusion: This study provides support for a lack of an influence of mobile phone exposure on cognitive performance.  (No effect) |
| Movvahedi et al. (2014)  (3^rd^ tier) | Single-blind, randomized cross-over study  Children (boys, n = 60),  7–10 years or 8–10 years (contradictory data) | Mobile phone, GSM 900 MHz:  Manufacturer's specification:  2 conditions: sham exposure and exposure (highest SAR: 1.45 W/kg;  exposure of the head  Exposure duration: 2 x 10 min, separated by 30 min | Cognitive functions:  visual reaction time and short-term memory  Standardized computer-based tests | Statistically significant better performance of the short-term memory *(according to the authors; statistical significance of the results is not comprehensible)*  Conclusion: Short-term exposure to RF EMF might lead to a better performance of the short-term memory in children.  (Effect found) |
| Preece et al. (2005)  (1^st^ tier) | Randomized cross-over study  Children (boys and girls, n = 18)  10–12 years | Mobile phone, GSM 902 MHz:  Measurement in a phantom: 3 conditions: sham exposure, low power (peak: 0.2 W, mean: 0.025 W) and high power (peak: 2 W, mean: 0.25 W, maximum brain SAR: approximately 0.28 W/kg)  exposure of the head  Exposure duration:  3 x about 30–35 min on sequential days at approximately the same time of the day | Cognitive functions:  reaction time, accuracy and sensitivity index  Computerized cognitive tests from Cognitive Drug Research (CDR) | No statistically significant effects  Conclusion: No effect of mobile phone exposure on reaction time in children.  (No effect) |
| Riddervold et al. (2008)  (1^st^ tier) | Double-blind, randomized cross-over study  Adolescents (male and female, n = 40)  15–16 years  Additionally, adults (n = 40), 25–40 years, were investigated | UMTS mobile phone base station, 2,140 MHz:  Measurement: 4 conditions: sham exposure, continuous wave (CW), a signal modulated as UMTS and UMTS including all control features (electric field strength: 0.9–2.2 V/m);  far-field condition  Exposure duration:  4 x 45 min, separated by at least 24 h at the same time of the day | Cognitive functions:  simple and complex reaction time, vigilance, attention and memory  CANTAB software  Subjective symptoms:  e.g., headache, concentration difficulties, dizziness, nausea  Questionnaire | No statistically significant effects  Conclusion: UMTS EMF do not reduce general performance in cognitive tests.  (No effect) |

Note: If not stated otherwise, only statistically significant results are provided. In the column „Exposure“ the sham exposure duration is also listed in „Exposure duration“. (Example: If there was an exposure condition and a sham exposure and both with a duration of 30 min, this is noted as „2 x 30 min“ in „Exposure duration“).

Abbreviations: 2G/3G – 2^nd^ resp. 3^rd^ generation of mobile communication systems, CW – Continuous Wave, EEG – Electroencephalography, GSM – Global System for Mobile Communications, psSAR – peak spatial SAR, SAR – Specific Absorption Rate, UMTS – Universal Mobile Telecommunications System, WCDMA – Wideband Code Division Multiple Access
