# Supplementary material for: Systematic review of the physiological and health-related effects of radiofrequency electromagnetic field exposure from wireless communication devices on children and adolescents in experimental and epidemiological human studies
Source: PLoS One. 2022 Jun 1;17(6):e0268641. doi: 10.1371/journal.pone.0268641 (PMC9159629; doi:10.1371/journal.pone.0268641)
Supplement: S10 Table — (DOCX) [file pone.0268641.s013.docx]

**S10 Table. Experimental studies on physiological parameters in children and adolescents (n = 2).**

| Author (Year)  (OHAT study quality) | Study type  Study population  Age | Exposure source Assessment method  Exposure conditions | Endpoints Assessment method | Results  Conclusion according to authors  (Effect categorization according to authors) |
| --- | --- | --- | --- | --- |
| Choi et al. (2014)  (1^st^ tier) | Double-blind, randomized cross-over study  Adolescents (male and female, n = 26)  14–17 years  Additionally, adults (n = 26), 28.4 ± 5.1 years, were investigated | WCDMA module, 1,950 MHz:  Measurement in a phantom:  2 conditions: sham exposure and exposure (psSAR_1g_: 1.57 W/kg);  exposure of the head  Exposure duration: 2 x 32 min, separated by 1–10 days at the same time of the day | Physiological parameters:  heart rate, respiratory rate and heart rate variability  Computerized polygraph  Subjective symptoms:  throbbing, itching, warmth, fatigue, headache, dizziness, nausea, and palpitation  Oral survey | No statistically significant effects  Conclusion: Short-term WCDMA RF EMF generated no significant changes in physiological parameters or subjective symptoms.  (No effect) |
| Lindholm et al. (2011)  (1^st^ tier) | Double-blind cross-over study  Adolescents (male, n = 26)  14–15 years | Mobile phone, GSM 902 MHz:  Calculated and modelled:  2 conditions: sham exposure and exposure (SAR_10g_: 2 W/kg);  exposure of the head  Exposure duration: 2 x 15 min, separated by 5 min | Thermal and local blood flow responses:  ear canal temperature and skin temperature, local cerebral blood flow, peripheral blood circulation and continuous blood pressure  Infrared camera, near-infrared spectroscopy, ECG, digital blood pressure measurement | No statistically significant effects  Conclusion: Short-term exposure to RF EMF produced by a GSM mobile phone does not  cause any significant increase in local ear canal temperatures or superficial cerebral blood flow among adolescents.  (No effect) |

Note: Only statistically significant results are provided. In the column „Exposure“ the sham exposure duration is also listed in „Exposure duration“. (Example: If there was an exposure condition and a sham exposure and both with a duration of 30 min, this is noted as „2 x 30 min“ in „Exposure duration“).

Abbreviations: ECG – Electrocardiography, GSM – Global System for Mobile Communications, psSAR – peak spatial SAR, SAR – Specific Absorption Rate, WCDMA – Wideband Code Division Multiple Access
