# Supplementary material for: Systematic review of the physiological and health-related effects of radiofrequency electromagnetic field exposure from wireless communication devices on children and adolescents in experimental and epidemiological human studies
Source: PLoS One. 2022 Jun 1;17(6):e0268641. doi: 10.1371/journal.pone.0268641 (PMC9159629; doi:10.1371/journal.pone.0268641)
Supplement: S11 Table — (DOCX) [file pone.0268641.s014.docx]

| **Endpoint** | **Study design** | **Initial confidence** | **Factors decreasing confidence** | **Factors increasing confidence** | **Confidence in the body of evidence** | **Direction**  **(effect or no effect)** | **Level of evidence for health effect** |
| --- | --- | --- | --- | --- | --- | --- | --- |
| Subjective symptoms | Cohort study | Moderate | Risk of bias | - | Low | Effect | Low |
| Subjective symptoms | Cross-sectional study | Low | Risk of bias | - | Very low | Effect | Inadequate |
| Cognition | Cohort study | Moderate | Unexplained inconsistency | - | Low | Effect | Low |
| Cognition | Cross-sectional study | Low | Risk of bias | - | Very low | Effect | Inadequate |
| Behavior | Cohort study | Moderate | Risk of Bias, unexplained inconsistency | - | Very low | Effect | Inadequate |
| Behavior | Cross-sectional study | Low | - | - | Low | Effect | Low |
| Infant development | Cohort study | Moderate | Risk of bias, unexplained inconsistency | - | Very Low | Effect | Inadequate |
| Childhood cancer | Case-control study | Moderate | Risk of bias | - | Low | No effect | Inadequate |
