# Supplementary material for: Systematic review of the physiological and health-related effects of radiofrequency electromagnetic field exposure from wireless communication devices on children and adolescents in experimental and epidemiological human studies
Source: PLoS One. 2022 Jun 1;17(6):e0268641. doi: 10.1371/journal.pone.0268641 (PMC9159629; doi:10.1371/journal.pone.0268641)
Supplement: S12 Table — (DOCX) [file pone.0268641.s015.docx]

| **Endpoint** | **Study design** | **Initial confidence** | **Factors decreasing confidence** | **Factors increasing confidence** | **Confidence in the body of evidence** | **Direction**  **(effect or no effect)** | **Level of evidence for health effect** |
| --- | --- | --- | --- | --- | --- | --- | --- |
| Cognition | Human controlled trial | High | Unexplained inconsistency, small study groups | - | Low | No Effect | Inadequate |
| Brain activity | Human controlled trial | High | Risk of Bias, unexplained inconsistency, small study groups | - | Very low | Unclear | Inadequate |
| Physiological parameters | Human controlled trial | High | Small study groups, small number of studies | - | Low | No Effect | Inadequate |
